# Hexafluoroisopropanol Mediated Ring-Opening Reactions of Epoxides with Indoles Under Catalyst-Free Conditions

**DOI:** 10.3390/molecules31173108

**Published:** 2026-09-04

**Authors:** Yangmin Ma, Ding Ma, Zhichao Wang, Murong Zhan, Qian Jiao, Sen Yang

**Affiliations:** Key Laboratory of Chemical Additives, China National Light Industry, College of Chemistry and Chemical Engineering, Shaanxi University of Science and Technology, Xi’an 710021, China; 240811030@sust.edu.cn (D.M.); bs240811027@sust.edu.cn (M.Z.); 240811015@sust.edu.cn (Q.J.); 240812199@sust.edu.cn (S.Y.)

**Keywords:** indole derivatives, HFIP, catalyst-free, ring-opening, epoxides

## Abstract

An efficient, catalyst-free, and room-temperature protocol has been developed for the ring-opening reactions of ring cyclic ethers with indoles in Hexafluoroisopropanol (HFIP). Under these mild conditions, a diverse range of indoles smoothly reacted with oxirane within 6 h, affording the corresponding C3-alkylated indoles in good-to-excellent yields. Notably, this method also shows promising reactivity toward four-membered oxetanes. Furthermore, stereospecific investigations employing enantiopure (R)-styrene oxide afforded the corresponding products with high enantiomeric excess, suggesting an S_N_2 pathway with inversion of configuration. The unique hydrogen-bonding ability and acidity of HFIP are crucial for driving this facile transformation. This protocol offers a green, atom-economical, and sustainable approach for constructing structurally diverse indole derivatives, showcasing high practical utility for organic synthesis.

## 1. Introduction

Indole and its derivatives are widely found in various natural products, pharmaceuticals, and agrochemicals [1,2,3,4]. Because of their practical importance, the chemical modification of indoles, particularly at the C-3 position, is a significant topic in organic synthesis [5,6,7,8]. Among these derivatives, 3-alkylindoles exhibit a wide range of biological and pharmacological activities, and they also serve as useful intermediates for further chemical transformations [9,10,11,12]. Therefore, developing simple, efficient, and mild methods to synthesize 3-alkylindole derivatives is highly desirable.

The ring-opening reactions of epoxides with heteroatom nucleophiles (N, O, S) are well established [13,14]. However, since the central aim of synthetic chemistry is to construct complex molecular skeletons, the formation of C–C bonds is fundamentally more important than that of C–heteroatom bonds. Regrettably, the progress in ring-opening reactions employing carbon nucleophiles has been rather slow. For the reaction of indoles with styrene oxide, several Brønsted or Lewis acid catalysts, such as InBr_3_ and InCl_3_, have been employed to achieve good yields [15,16]. Additionally, a polymer-supported indium catalyst was reported for the reaction of 1-methylindole with styrene oxide, but it gave a relatively low yield [17]. Other catalytic systems, including nano-metal oxides, lanthanide triflates, and silica gel under high pressure, have also been investigated, but they generally require elevated temperatures or specialized equipment [18,19,20]. Recently, Guerra and co-workers developed a solvent-free protocol using exfoliated graphite oxide (eGO) as a catalyst [21]. However, this method suffers from an excessively long reaction time of 96 h. Therefore, most existing procedures for the ring-opening of styrene oxide with indoles still rely on the use of catalysts and often require harsh reaction conditions or prolonged reaction times.

Fluoroalcohols are well known for their strong ionizing power, high hydrogen-bonding ability, and distinctive solvent properties, and they have been widely employed to promote challenging organic transformations [22,23]. Fluorinated compounds, particularly per and polyfluoroalkyl substances, have attracted increasing attention because of their persistence and environmental concerns, and the broader use and regulation of fluorinated chemicals have therefore become important issues in modern chemistry. As emphasized in the studies of Ameduri, fluorinated compounds and fluoropolymers possess distinctive physicochemical properties arising from the strong C–F bond, which can provide unique performance advantages in applications where conventional materials or chemicals may be inadequate [24]. Thus, although HFIP should not be regarded as an intrinsically green solvent solely on the basis of its performance in synthesis, its use can be justified when its distinctive properties enable efficient transformations under mild conditions and eliminate the need for additional catalysts or harsher reaction conditions. Previous studies demonstrated that the combination of HFIP and Brønsted acid (TfOH) enables the rapid reductive ring-opening of epoxides and unlocks the Friedel–Crafts arylation of epoxides with a diverse array of arenes [25,26]. More recently, HFIP has also been shown to promote ring-opening transformations under catalyst-free conditions, as demonstrated by the ring-opening cyclization of donor acceptor cyclopropanes with primary amines [27].

**Scheme 1:**
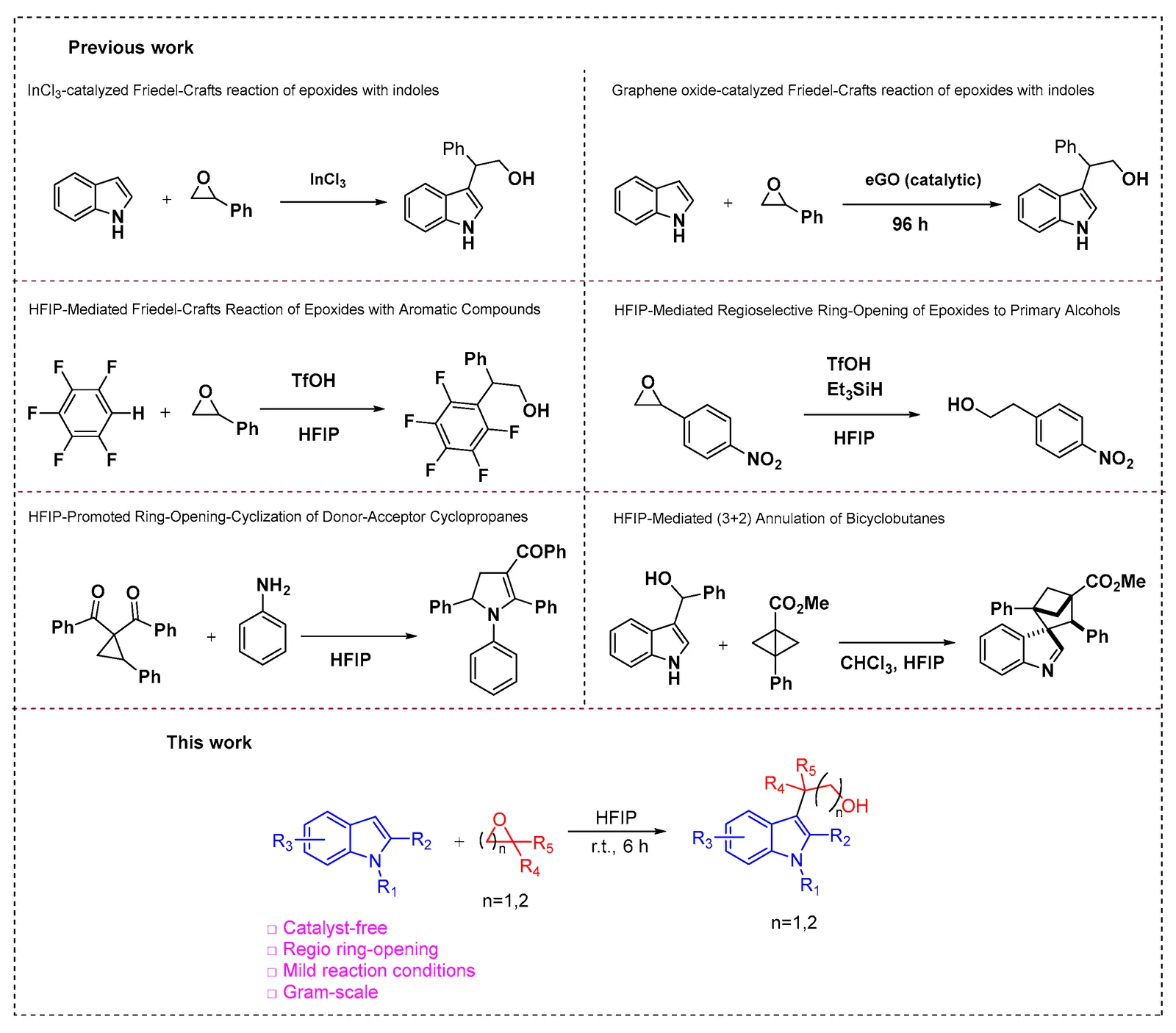
Previous strategies for the ring-opening of cyclic ethers and HFIP-mediated transformations and this work [15,21,25,26,27,28].

In addition, recent studies have further demonstrated the utility of HFIP in catalyst-free protocols without the addition of external Lewis or Brønsted acid catalysts. For instance, HFIP has been shown to effectively mediate the formal (3+2) annulation of bicyclobutanes with indolyl alcohols, as well as promote Friedel–Crafts alkylation reactions, demonstrating its ability to function as both an activator and solvent without the need for external catalysts [28]. These studies highlight the broader potential of HFIP-assisted catalyst-free processes, in which its distinctive hydrogen-bonding and ionizing properties can facilitate substrate activation and stabilize reactive intermediates. Given the robust nucleophilicity of indoles, it was hypothesized that the dense hydrogen-bonding network of HFIP alone could provide sufficient electrophilic activation without the need for exogenous acids. In pursuit of more sustainable and operationally simple methodologies, it is herein demonstrated that HFIP alone can function as a highly efficient solvent and activator for the catalyst-free ring-opening reactions of epoxides with indoles at room temperature. A comparison of these previous strategies and our present work is visually summarized in Scheme 1.

## 2. Results and Discussion

### 2.1. Condition Optimization and Solvent Screening

The HFIP-mediated Friedel–Crafts reaction between indoles and epoxides was investigated as the initial step, and the influence of various additives on this transformation was evaluated (Table 1).

Initially, drawing inspiration from Moran’s pioneering work [25], Brønsted acids such as TFA, HCl, and TfOH were employed as catalysts. Delightfully, the model reaction of indole (**1a**) and styrene oxide (**2a**) proceeded smoothly to afford the desired product **3a** in excellent isolated yields of 81%, 69%, and 88%, respectively (Table 1, entries 1–3). Other metal triflates, such as In(OTf)_3_ and Sc(OTf)_3_, were also screened, delivering the product in similarly high yields of over 80% (Table 1, entries 4 and 5). Intriguingly, when the reaction was performed in the complete absence of any catalyst, the ring-opening process still occurred efficiently, yielding **3a** in an even higher yield of 84% (Table 1, entry 6). This pivotal result clearly demonstrated that the unique property of HFIP alone is sufficient to drive this transformation without requiring any external catalytic activation.

With this catalyst-free protocol as an encouraging starting point, the stoichiometry of the reactants was subsequently optimized (Table 1, entries 7–10). It was observed that increasing the amount of indole (**1a**) significantly benefited the efficiency; when 4.0 equivalents of **1a** were reacted with 1.0 equivalent of styrene oxide (**2a**), the yield of **3a** was successfully enhanced to 89% (Table 1, entry 10). In addition, to confirm the optimal reaction time, shorter reaction intervals were evaluated. Reducing the reaction time to 5 h or 3 h resulted in decreased yields of 78% and 52%, respectively (Table 1, entries 11 and 12), indicating that 6 h is essential for maximum conversion. Subsequently, the impact of concentration and solvent systems was systemically investigated. Altering the concentration of HFIP to 1.0 M or 0.25 M led to a noticeable decrease in product yields (Table 1, entries 13 and 14). Furthermore, when HFIP was replaced with conventional organic solvents, such as EtOH, MeCN, toluene, or *i*-PrOH, the reaction was completely suppressed, and no desired product was detected (Table 1, entries 15–18). To determine whether this activation is generally applicable to highly fluorinated alcohols or specific to HFIP, 2,2,2-trifluoroethanol (TFE) was subsequently evaluated. Interestingly, no reaction occurred in TFE after 6 h (Table 1, entry 19), and extending the reaction time to 24 h afforded only a trace amount of product (Table 1, entry 20). To gain deeper mechanistic insights into the role of HFIP as an activator, dilution experiments using an inert halogenated solvent were conducted. When HFIP was diluted with dichloromethane (DCM) to concentrations of 5% (1:19 *v*/*v*) and 10% (1:9 *v*/*v*), the reaction was completely suppressed (Table 1, entries 21 and 22). Increasing the proportion of HFIP to approximately 20% (1:4 *v*/*v*) afforded only a trace amount of the product (Table 1, entry 23). Utilizing a mixed solvent system of EtOH and HFIP also resulted in a significantly diminished yield of 63% (Table 1, entry 24). Based on these systematic screenings, the optimized reaction conditions were established as **1a** (4.0 equiv.) and **2a** (1.0 equiv.) in HFIP (0.5 M) at room temperature for 6 h.

### 2.2. Scope of Reaction Substrates

With the optimal reaction conditions established, the substrate scope of diverse indoles was subsequently investigated to evaluate the generality of this catalyst-free protocol (Table 2).

First, N-protected indoles, including N-methyl and N-phenyl derivatives, reacted smoothly with styrene oxide (**2a**) to afford the desired 3-alkylindoles in good yields (**3b**, 84%; **3e**, 75%). Similarly, C2-substituted indoles bearing methyl or phenyl groups were well-tolerated, providing the corresponding products in high yields (**3c**, 82%; **3d**, 85%).

Next, the influence of substituents on the benzenoid ring of indole was systematically examined. Indoles bearing electron-donating methyl groups at the C-4 to C-7 positions reacted with **2a** to furnish the desired products in moderate-to-good yields (42–73%, **3f**–**3i**). Other electron-donating groups, such as methoxy, as well as various halogens (F, Cl, Br, I), were also compatible with this mild protocol, delivering the target compounds in 41–82% yields (**3j**–**3q**). Furthermore, electron-withdrawing indoles substituted with strongly deactivating groups, including cyano, nitro, and ester functionalities, successfully participated in the transformation, affording the corresponding products in moderate yields (40–60%, **3r**–**3v**).

Notably, a clear steric effect was observed during the evaluation of the substituent positions. When substituents were located at the C-4 position of the indole core (e.g., **3f**, **3m**, **3o**, and **3t**), the isolated yields (41–50%) were generally lower compared to those with substituents at other positions. This decrease in reaction efficiency is presumably attributed to the increased steric hindrance around the nucleophilic C-3 center, although synthetically useful yields could still be secured.

To further demonstrate the versatility of this protocol, the substrate scope with respect to various epoxides was subsequently evaluated (Table 3).

First, several substituted styrene oxide derivatives were examined. Styrene oxide with a 4-bromo group was found to work well with unsubstituted indole, affording product **3aa** in 88% yield. 4-Bromostyrene oxide was also tested with 7-methylindole and 5-nitroindole, and both reactions gave the products **3ab** and **3ac** in 84% and 40% yields, respectively. Furthermore, styrene oxides substituted at the meta-position with either an electron-donating methyl group (**3ad**, 70%) or an electron-withdrawing cyano group (**3ae**, 83%) successfully participated in the transformation. An ortho-substituted epoxide, ortho-methoxystyrene oxide, was also evaluated and afforded the product **3af** in 80% yield. To expand the scope beyond terminal epoxides, 1,1-diphenylethylene oxide containing two substituents at one carbon atom was tested, furnishing the product **3ag** in 63% yield. Beyond aromatic epoxides, more challenging aliphatic and functionalized terminal epoxides were explored to showcase the synthetic utility of this method. Encouragingly, phenyl glycidyl ether and epichlorohydrin underwent smooth ring-opening reactions under the standard catalyst-free conditions, furnishing the target compounds **3ah** (52%) and **3ai** (55%) in moderate yields. Notably, phenyl glycidyl ether and epichlorohydrin are categorized as aliphatic substituted epoxides. Owing to the lack of a phenyl ring for charge stabilization, the regioselectivity of the reaction is dictated exclusively by steric effects. As a result, the indole nucleophile preferentially attacks the less hindered terminal CH_2_, thereby affording the secondary alcohol as the ring-opened product.

**Scheme 2:**
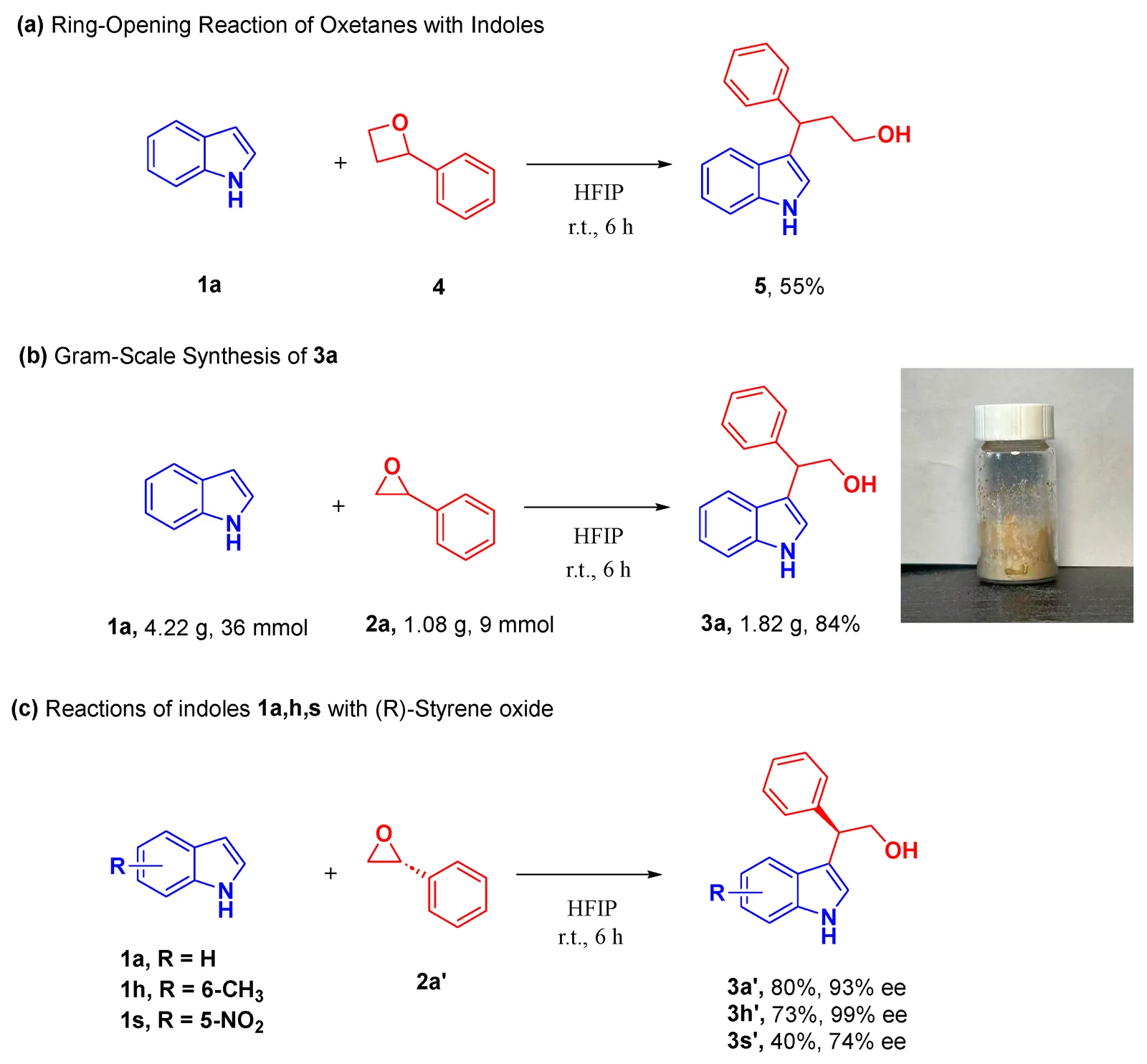
Synthetic utility and stereochemical investigations.

To further test how useful this method is and to better understand how the reaction works, a few additional experiments were done (Scheme 2). First, a less reactive four-membered cyclic ether, 2-phenyloxetane (4), was tried (Scheme 2a). These types of ethers have lower ring strain than epoxides, so they usually need strong acids or harsh conditions to open up. But under our standard conditions, it reacted with indole (**1a**) at room temperature and gave product **5** in 55% yield. This shows that HFIP can help activate even these harder to react substrates.

To demonstrate the practical utility and scalability of this catalyst-free protocol, a preparative-scale synthesis was conducted. Pleasingly, the reaction could be readily scaled up without a significant decrease in efficiency. As illustrated in Scheme 2b, the reaction of indole (**1a**, 4.22 g, 36 mmol) with styrene oxide (**2a**, 1.08 g, 9 mmol) in HFIP at room temperature proceeded smoothly, affording the desired product 3a in 84% isolated yield (1.82 g) after 6 h.

Finally, the stereochemical pathway was investigated by reacting different indoles (**1a, 1h, 1s**) with enantiopure (R)-styrene oxide (**2a**′) (Scheme 2c). The reactions gave the chiral products **3a′**, **3h′**, and **3s′** in 40–80% yields, with very high enantiomeric excess (74–99% ee). Such high stereospecificity suggests that the nucleophilic attack follows an S_N_2 mechanism, meaning the attack happens at the benzylic carbon with inversion of configuration. This also indicates that HFIP hydrogen-bonding network activates the epoxide effectively, while at the same time preventing the formation of a free carbocation intermediate, which would otherwise lead to racemization.

### 2.3. Mechanism Investigation

**Scheme 3:**
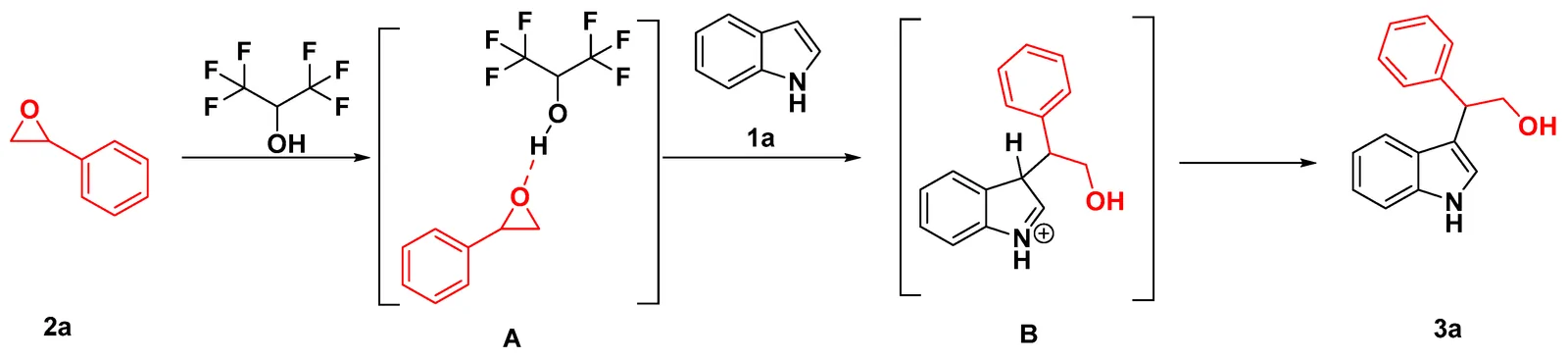
A plausible reaction pathway.

Based on the aforementioned experimental results, particularly the strict stereospecificity observed in the chiral experiments, and previous literature reports, a plausible mechanism for this HFIP mediated ring-opening reaction is proposed in Scheme 3. HFIP is known to promote Friedel–Crafts type C–C bond formation via C–O bond cleavage in substrates such as aromatic aldehyde hydrates and propargyl alcohols [29,30,31,32,33]. Consistent with this reactivity, the present reaction is proposed to follow a similar pathway. Initially, HFIP forms a strong hydrogen-bonding network with the oxygen of styrene oxide (**2a**), which activates the epoxide and polarizes the C–O bond to give intermediate A.

Subsequently, the C3-position of indole (**1a**) attacks the activated epoxide. Regioselectivity is dictated by the phenyl ring, which stabilizes the developing positive charge at the benzylic carbon, ensuring exclusive attack at this site and generating the zwitterionic Wheland-type indolium intermediate, intermediate B. Finally, proton transfer from the indolium moiety to the alkoxide oxygen triggers rearomatization of the indole ring, affording the C3-alkylated primary alcohol (**3a**) and regenerating HFIP for the next reaction cycle.

## 3. Materials and Methods

### 3.1. General Information

^1^H NMR spectra were recorded on a Bruker Ascend 400 (400 MHz) and a Bruker Ascend 600 (600 MHz) spectrometer (Bruker Switzerland AG, Fällanden, Switzerland). Data for ^1^H NMR are reported as follows: chemical shifts in ppm relative to tetramethylsilane as an internal standard in CDCl_3_, integration, multiplicity (s = singlet, d = doublet, t = triplet, q = quartet, m = multiplet, br = broad), coupling constants (Hz), and assignment. ^13^C NMR spectra were recorded on a Bruker Ascend 400 (100 MHz) and 600 (150 MHz) spectrometer (Bruker Switzerland AG, Fällanden, Switzerland) with complete proton decoupling. Chemical shifts for ^13^C NMR were reported in ppm relative to the residual solvent as an internal standard. ^19^F NMR spectra were acquired at 376 MHz on a Bruker Ascend 400 spectrometer. High resolution mass spectra were acquired on a Waters G2-S Qtof mass spectrometer (Milford, MA, USA). Fourier IR data were determined on a VECTOR II spectrometer in Bruker Germany (Billerica, MA, USA). All reagents and solvents were purchased from commercial sources and used without further purification.

All commercially available chemicals and solvents were used directly without further purification unless otherwise stated. Specifically, all indole starting materials were purchased from Energy Chemical (Anhui, China) and Macklin (Shanghai, China) with a purity of >99%. Hexafluoroisopropanol (HFIP, purity: 99.5%) was also purchased from Energy Chemical (Anhui, China). Most terminal epoxides used in the substrate scope were commercially available and used as received. The specific starting materials, including substituted styrene oxides (**2b**–**e**) and 2-phenyloxetane (**4**), were not commercially sourced and were synthesized in our laboratory according to known literature methods. The detailed synthetic procedures for these specific compounds are described below in Section 3.1.1 and Section 3.1.2, respectively.

#### 3.1.1. The Compound **2b**–**e** Were Prepared According to the Literature [34,35]

A solution of NaH (11 mmol, 2.2 equiv.) and Me_3_SI (11 mmol, 2.2 equiv.) in DMSO (7 mL) and THF (6 mL) was stirred for 30 min at room temperature in a 50 mL flask under an Ar atmosphere. Then a solution of aldehyde 6 (5 mmol, 1.0 equiv.) in THF was added dropwise at 0 °C. After complete addition, the mixture was warmed to room temperature and allowed to stir for 12 h. The mixture was quenched with sat. NH_4_Cl and diluted with EtOAc. Then the mixture was separated and the aqueous layer was back-extracted with EtOAc. The organic layer was washed with brine and dried over Na_2_SO_4_. The solvent was removed under reduced pressure and the residue was purified by column chromatography to give the desired epoxides.

#### 3.1.2. The Compound 4 Was Prepared According to the Literature [36]

3-Chloro-1-phenyl-1-propanone **7** (5 mmol, 1.0 equiv) was dissolved in anhydrous THF (40 mL), and KO*t*-Bu (15 mmol, 3.0 equiv) was added. The mixture was stirred at room temperature for 4 h. Upon completion of the reaction, water was added to quench the reaction, and the mixture was diluted with ethyl acetate. The resulting mixture was transferred to a separatory funnel, and the layers were separated after thorough shaking and standing. The aqueous layer was extracted twice with ethyl acetate. The combined organic layers were washed successively with saturated brine and dried over anhydrous sodium sulfate. After filtration, the solvent was removed under reduced pressure, and the crude product was purified by column chromatography on silica gel to afford the compound **4**.

### 3.2. Experimental Procedure for All the Products ***3a**–**3v***, ***3aa**–**3ai***, ***3a′***, ***3h′***, ***3s′***, and ***5***

The indole **1** (2.0 mmol, 4.0 equiv.) and Epoxides **2** (0.5 mmol, 1 equiv.) in HFIP (1.0 mL) was stirred at room temperature for 6 h. After the filtration and evaporation of the solvents under reduced pressure, the crude products were purified by column chromatography on silica gel to afford the desired products **3a**–**3v**, **3aa**–**3ai**, **3a′**, **3h′**, **3s′**, and **5**.

### 3.3. Characterization Data for All the Products ***3a**–**3v***, ***3aa**–**3ai***, ***3a′***, ***3h′***, ***3s′***, and ***5***

**2-(1*H*-indol-3-yl)-2-phenylethan-1-ol (3a):**white solid; yield: 97.3 mg (82%), R_f_ = 0.4 (petroleum ether:ethyl aceate = 3:1), m.p. 119–120 °C. **^1^H NMR (400 MHz, CDCl_3_):**δ 8.09 (br s, 1H), 7.47 (d, *J* = 4.0 Hz, 1H), 7.38–7.29 (m, 5H), 7.25–7.15 (m, 2H), 7.13 (s, 1H), 7.09–7.02 (m, 1H), 4.54–4.46 (m, 1H), 4.29–4.17 (m, 2H), 1.27 (br s, 1H). **^13^C NMR (100 MHz, CDCl_3_):**δ 141.6, 136.5, 128.7, 128.3, 127.0, 126.8, 122.4, 121.9, 119.6, 119.4, 116.1, 111.2, 66.5, 45.7. **IR (KBr):**3546, 3411, 1622, 1490, 1388, 1093, 1006, 744, 698 cm^−1^. **HRMS (ESI):**calculated for C_16_H_16_NO ([M + H]^+^), *m*/*z* 238.1230, found 238.1232. **Chiral HPLC:**IB (isocratic, heptane: isopropanol) = 85:15, 0.5 mL min^−1^, UV at 254 nm, t(*S*) = 29.3 min, t(*R*) = 35.2 min. **(*R*)-2-(1*H*-indol-3-yl)-2-phenylethan-1-ol (3a′)**: 93% ee.
**2-(1-Methyl-1*H*-indol-3-yl)-2-phenylethan-1-ol (3b):**yellow oil; yield: 105.6 mg (84%), R_f_ = 0.45 (petroleum ether:ethyl aceate = 3:1). **^1^H NMR (400 MHz, CDCl_3_):**δ 7.49 (d, *J* = 8.0 Hz, 1H), 7.41–7.29 (m, 5H), 7.26–7.22 (m, 1H), 7.07 (t, *J* = 6 Hz, 1H), 6.98 (s, 1H), 4.50 (t, J = 6.8 Hz, 1H), 4.27–4.14 (m, 2H), 3.78 (s, 3H), 1.74 (br s, 1H). **^13^C NMR (100 MHz, CDCl_3_):**δ 141.8, 137.3, 128.6, 128.3, 127.5, 126.7, 121.9, 119.5, 119.1, 114.5, 109.3, 66.5, 45.6, 32.8. **IR (KBr):**3541, 3379, 2929, 1614, 1473, 1373, 1238, 1053, 744, 700 cm^−1^. **HRMS (ESI):**calculated for C_17_H_18_NO ([M + H]^+^), *m*/*z* 252.1385, found 252.1388.
**2-(1,2-Dimethyl-1*H*-indol-3-yl)-2-phenylethan-1-ol (3c):**yellow oil; yield: 108.8 mg (82%), R_f_ = 0.32 (petroleum ether:ethyl aceate = 2:1). **^1^H NMR (600 MHz, CDCl_3_):**δ 7.47 (d, *J* = 6.0 Hz, 1H), 7.35–7.30 (m, 2H), 7.28–7.23 (m, 3H), 7.18–7.11 (m, 2H), 7.00 (t, *J* = 6 Hz, 1H), 4.50 (t, *J* = 6 Hz, 1H), 4.32 (d, *J* = 12 Hz, 2H), 3.65 (s, 3H), 2.36 (s, 3H), 1.60 (br s, 1H). **^13^C NMR (150 MHz, CDCl_3_):**δ 142.0, 137.0, 135.2, 128.4, 128.0, 126.8, 126.3, 120.8, 119.3, 119.2, 109.4, 109.1, 65.2, 45.5, 29.4, 10.7. **IR (KBr):**3544, 3379, 2933, 1600, 1467, 1367, 1249, 1029, 744, 700 cm^−1^. **HRMS (ESI):**calculated for C_18_H_20_NO ([M + H]^+^), *m*/*z* 266.1545, found 266.1548.
**2-(1-Methyl-2-phenyl-1*H*-indol-3-yl)-2-phenylethan-1-ol(3d):**yellow oil; yield: 139.2 mg (85%), R_f_ = 0.37 (petroleum ether:ethyl aceate = 2:1). **^1^H NMR (400 MHz, CDCl_3_):**δ 7.62 (d, *J* = 7.6 Hz, 1H), 7.47 (s, 3H), 7.40–7.14 (m, 9H), 7.11 (t, *J* = 7.2 Hz, 1H), 4.45–4.21 (m, 3H), 3.60 (s, 3H), 1.56 (br s, 1H). **^13^C NMR (100 MHz, CDCl_3_):**δ 142.2, 140.5, 137.5, 131.7, 131.0, 128.6, 128.5, 128.4, 128.0, 126.5, 126.3, 121.9, 120.5, 119.7, 111.3, 109.7, 65.4, 45.6, 31.0. **IR (KBr):**3546, 3369, 2921, 1602, 1473, 1369, 1251, 1052, 744, 702 cm^−1^. **HRMS (ESI):**calculated for C_23_H_22_NO ([M + H]^+^), *m*/*z* 328.1701, found 328.1709.
**2-Phenyl-2-(1-phenyl-1*H*-indol-3-yl)ethan-1-ol (3e):**yellow oil; yield: 117.5 mg (75%), R_f_ = 0.48. (petroleum ether:ethyl aceate = 5:1). **^1^H NMR (400 MHz, DMSO-*d*6):**δ 7.58 (s, 1H), 7.56–7.48 (m, 5H), 7.39 (d, J = 8 Hz, 1H), 7.35–7.21 (m, 3H), 7.21 (t, *J* = 7.8 Hz, 2H), 7.13–7.08 (m, 2H), 6.97 (t, *J* = 7.6 Hz, 1H), 4.81 (t, *J* = 5.2 Hz, 1H), 4.30 (t, *J* = 7.2 Hz, 1H), 4.06–3.90 (m, 2H). **^13^C NMR (100 MHz, DMSO-*d*6):**δ 143.7, 139.7, 135.6, 130.3, 129.1, 128.8, 128.6, 126.5, 125.8, 124.0, 122.9, 120.3, 120.0, 118.7, 110.7, 65.5, 45.7. **IR (KBr):**3455, 3058, 1642, 1493, 1035, 915, 750, 700 cm^−1^. **HRMS (ESI):**calculated for C_22_H_20_NO ([M + H]^+^), *m*/*z* 314.1545, found 314.1541.
**2-(4-Methyl-1*H*-indol-3-yl)-2-phenylethan-1-ol (3f):**green solid; yield: 52.7 mg (42%), R_f_ = 0.3 (petroleum ether:ethyl aceate = 3:1), m.p. 189–190 °C. **^1^H NMR (400 MHz, DMSO-*d*6):**δ 10.91 (s, 1H), 7.27–7.12 (m, 9H), 6.94–6.68 (m, 1H), 6.65–6.57 (m, 1H), 4.73 (s, 1H), 4.59 (d, *J* = 6 Hz, 1H), 4.01–3.81 (m, 2H), 2.41 (s, 3H). **^13^C NMR (100 MHz, DMSO-*d*6):**δ 145.3, 137.0, 129.9, 129.0, 128.4, 126.2, 126.0, 123.1, 121.3, 120.5, 116.3, 109.8, 66.9, 46.6, 20.9. **IR (KBr):**3556, 3050, 2920, 2870, 1636, 1455, 1225, 1107, 790, 700 cm^−1^. **HRMS (ESI):**calculated for C_17_H_18_NO ([M + H]^+^), *m*/*z* 252.1388, found 252.1389.
**2-(5-Methyl-1*H*-indol-3-yl)-2-phenylethan-1-ol (3g)**:white solid; yield: 75.4 mg (60%), R_f_ = 0.43 (petroleum ether:ethyl aceate = 3:1), m.p. 140–141 °C. **^1^H NMR (400 MHz, CDCl_3_):**δ 7.99 (br s, 1H), 7.37–7.34 (m, 4H), 7.31 (t, *J* = 6.0 Hz, 1H), 7.26–7.22 (m, 3H), 7.07 (d, *J* = 1.8 Hz, 1H), 7.01 (d, *J* = 9 Hz, 1H), 4.47 (t, *J* = 6.0 Hz, 1H), 4.22–4.21 (m, 1H), 4.18–4.14 (m, 1H), 2.39 (s, 3H), 1.56 (br s, 1H). **^13^C NMR (100 MHz, CDCl_3_):**δ 141.7, 134.8, 128.9, 128.6, 128.3, 127.3, 126.7, 124.0, 122.1, 118.9, 115.5, 110.9, 66.5, 45.6, 21.5. **IR (KBr):**3558, 3437, 3049, 2921, 1640, 1456, 1226, 1105, 792, 700 cm^−1^. **HRMS (ESI):**calculated for C_17_H_18_NO ([M + H]^+^), *m*/*z* 252.1388, found 252.1390.
**2-(6-Methyl-1*H*-indol-3-yl)-2-phenylethan-1-ol (3h):** yellow oil; yield: 91.7 mg (73%), R_f_ = 0.48 (petroleum ether:ethyl aceate = 3:1). **^1^H NMR (400 MHz, CDCl_3_):**δ 7.94 (br s, 1H), 7.33–7.25 (m, 5H), 7.23–7.19 (m, 1H), 7.12 (s, 1H), 6.98 (s, 1H), 6.87 (d, *J* = 8.0 Hz, 1H), 4.44 (t, *J* = 6.8 Hz, 1H), 4.25–4.10 (m, 2H), 2.42 (s, 3H), 1.62 (br s, 1H). **^13^C NMR (100 MHz, CDCl_3_):**δ 141.7, 136.9, 132.1, 128.6, 128.3, 126.7, 124.8, 121.4, 121.3, 119.0, 115.7, 111.1, 66.4, 45.6, 21.6. **IR (KBr):**3554, 3419, 3020, 2929, 1637, 1454, 1228, 1105, 800, 700 cm^−1^. **HRMS (ESI):**calculated for C_17_H_18_NO ([M + H]^+^), *m*/*z* 252.1388, found 252.1391. **Chiral HPLC**:IB (isocratic, heptane: isopropanol) = 90:10, 0.5 mL min^−1^, UV at 254 nm, t(*S*) = 49.3 min, t(*R*) = 53.7 min. **(*R*)-2-(6-Methyl-1*H*-indol-3-yl)-2-phenylethan-1-ol (3h′)**: 99% ee.
**2-(7-Methyl-1*H*-indol-3-yl)-2-phenylethan-1-ol (3i)**:yellow oil; yield: 71.6 mg (57%), R_f_ = 0.43 (petroleum ether:ethyl aceate = 3:1). **^1^H NMR (400 MHz, CDCl_3_):**δ 8.02 (br s, 1H), 7.34–7.26 (m, 5H), 7.22–7.18 (m, 1H), 7.08 (d, *J* = 1.6 Hz, 1H), 6.99–6.93 (m, 2H), 4.46 (t, *J* = 6.8 Hz, 1H), 4.26–4.13 (m, 2H), 2.45 (s, 3H), 1.63(br s, 1H). **^13^C NMR (100 MHz, CDCl_3_):**δ 141.7, 136.1, 128.6, 128.3, 126.7, 126.6, 122.9, 121.6, 120.4, 119.9, 117.2, 116.6, 66.5, 45.8, 16.6. **IR (KBr):**3434, 3240, 2918, 1616, 1452, 1226, 1100, 1053, 786, 700 cm^−1^. **HRMS (ESI):**calculated for C_17_H_18_NO ([M + H]^+^), *m*/*z* 252.1388, found 252.1390.
**2-(7-Methoxy-1*H*-indol-3-yl)-2-phenylethan-1-ol (3j):**yellow oil; yield: 109.6 mg (82%), R_f_ = 0.31 (petroleum ether:ethyl aceate = 3:1). **^1^H NMR (400 MHz, CDCl_3_):**δ 8.57 (brs, 1H), 7.43–7.30 (m, 5H), 7.28 (t, *J* = 8.0 Hz, 1H), 7.00 (d, *J* = 8 Hz, 2H), 6.67 (d, *J* = 7.6 Hz, 1H), 6.56–6.53 (m, 1H), 4.65 (t, *J* = 7.2 Hz, 1H), 4.40–4.27 (m, 2H), 3.95 (s, 3H), 1.78 (br s, 1H). **^13^C NMR (100 MHz, CDCl_3_):**δ 145.4, 142.0, 128.9, 128.6, 128.3, 126.6, 125.6, 123.7, 118.2, 101.7, 101.6, 65.9, 55.4, 50.7. **IR (KBr):**3542, 3419, 2935, 1623, 1448, 1261, 1081, 800, 727 cm^−1^. **HRMS (ESI):**calculated for C_17_H_18_NO_2_ ([M + H]^+^), *m*/*z* 268.1338, found 268.1340.
**2-(5-methoxy-1*H*-indol-3-yl)-2-phenylethan-1-ol (3k):**black solid; yield: 106.5mg (77%), R_f_ = 0.34 (petroleum ether:ethyl aceate = 3:1), m.p. 102–103 °C. **^1^H NMR (400 MHz, CDCl_3_):**δ 7.99 (br s, 1H), 7.37–7.26 (m, 4H), 7.25–7.21 (m, 2H), 7.07 (s, 1H), 6.88–6.81 (m, 1H), 4.43 (t, *J* = 6.8 Hz, 1H), 4.26–4.10 (m, 2H), 3.75 (s, 3H), 1.60 (br s, 1H). **^13^C NMR (100 MHz, CDCl_3_):**δ 154.0, 141.5, 131.6, 128.7, 128.3, 127.5, 126.8, 122.7, 115.7, 112.5, 111.9, 101.3, 66.4, 55.9, 45.6. **IR (KBr):**3527, 3450, 3024, 2925, 1633, 1485, 1213, 1174, 1076, 800, 700 cm^−1^. **HRMS (ESI):**calculated for C_17_H_18_NO_2_ ([M + H]^+^), *m*/*z* 268.1338, found 268.1342.
**2-(5-Fluoro-1*H*-indol-3-yl)-2-phenylethan-1-ol (3l):**brown oil; yield: 85.4 mg (67%), R_f_ = 0.34 (petroleum ether:ethyl aceate = 3:1). **^1^H NMR (400 MHz, CDCl_3_):**δ 8.11 (br s, 1H), 7.33–7.29 (m, 4H), 7.26–7.22 (m, 2H), 7.18 (s, 1H), 7.05 (d, *J* = 8.8 Hz, 1H), 6.91 (t, *J* = 8.4 Hz, 1H), 4.45–4.34 (m, 1H), 4.24–4.12 (m, 2H), 1.16 (br s, 1H). ^1**3**^**C NMR (100 MHz, CDCl_3_):**δ 157.8 (d, *J* = 230.0 Hz), 141.3, 133.0, 128.8, 128.3, 127.5, 127.4, 127.0, 123.6, 116.3 (d, *J* = 4.7 Hz), 111.8 (d, *J* = 9.6 Hz), 110.8 (d, J = 26.2 Hz), 104.4 (d, J = 23.5 Hz), 66.4, 45.6. **^19^F NMR (376 MHz, CDCl_3_)**:δ −124.23. **IR (KBr):**3550, 3420, 3051, 1634, 1488, 1216, 1138, 1032, 815, 700 cm^−1^. **HRMS (ESI):**calculated for C_16_H_15_FNO ([M + H]^+^), *m*/*z* 256.1138, found 256.1139.
**2-(4-Chloro-1*H*-indol-3-yl)-2-phenylethan-1-ol (3m):**brown oil; yield: 55.7 mg (41%), R_f_ = 0.35 (petroleum ether:ethyl aceate = 3:1). **^1^H NMR (400 MHz, CDCl_3_):**δ 8.28 (s, 1H), 7.34–7.28 (m, 4H), 7.26–7.21 (m, 3H), 7.11 (s, 1H), 7.10–7.01 (m, 2H), 5.13 (t, *J* = 6.4 Hz, 1H), 4.26–4.09 (m, 2H), 1.67 (br s, 1H). **^13^C NMR (100 MHz, CDCl_3_):**δ 142.1, 137.8, 128.7, 128.6, 126.6, 126.4, 123.8, 123.5, 122.8, 116.5, 116.5, 110.0, 67.2, 45.1. **IR (KBr):**3558, 3444, 1649, 1487, 1338, 1200, 1031, 937, 754, 700 cm^−1^. **HRMS (ESI):**calculated for C_16_H_15_ClNO ([M + H]^+^), *m*/*z* 272.0842 and 274.0813, found 272.0844 and 274.0813.
**2-(5-Chloro-1*H*-indol-3-yl)-2-phenylethan-1-ol (3n):**red solid; yield: 101.9 mg (75%), R_f_ = 0.4 (petroleum ether:ethyl aceate = 3:1), m.p. 124–125 °C. **^1^H NMR (400 MHz, CDCl_3_):**δ 8.12 (br s, 1H), 7.39 (s, 1H), 7.34–7.29 (m, 4H), 7.28–7.26 (m, 1H), 7.17–7.10 (m, 2H), 4.40 (t, *J* = 6.4 Hz, 1H), 4.25–4.13 (m, 2H). **^13^C NMR (100 MHz, CDCl_3_):**δ 141.1, 134.8, 128.7, 128.1, 127.0, 125.3, 123.2, 122.7, 118.8, 116.0, 112.2, 66.4, 45.4. **IR (KBr):**3552, 3450, 1629, 1456, 1103, 1041, 798, 702 cm^−1^. **HRMS (ESI):**calculated for C_16_H_15_ClNO ([M + H]^+^), *m*/*z* 272.0842 and 274.0813, found 272.0844 and 274.0813.
**2-(4-Bromo-1*H*-indol-3-yl)-2-phenylethan-1-ol (3o):**white solid; yield: 79.1 mg (50%), R_f_ = 0.4 (petroleum ether:ethyl aceate = 3:1) m.p. 146–147 °C. **^1^H NMR (400 MHz, CDCl_3_):**δ 8.27 (br s, 1H), 7.36–7.26 (m, 6H), 7.23–7.20 (m, 1H), 7.13 (s, 1H), 6.99 (t, *J* = 7.9 Hz, 1H), 5.26 (t, *J* = 6.4 Hz, 1H), 4.26–4.21 (m, 1H), 4.15–4.09 (m, 1H), 1.66 (br s, 1H). **^13^C NMR (100 MHz, CDCl_3_):**δ 142.09, 137.74, 128.75, 128.56, 126.62, 125.08, 124.56, 123.96, 123.12, 116.98, 114.28, 110.60, 67.21, 44.57. **IR (KBr):**3566, 3446, 1633, 1427, 1334, 1191, 1031, 781, 702 cm^−1^. **HRMS (ESI):**calculated for C_16_H_15_BrNO ([M + H]^+^), *m*/*z* 316.0337 and 318.0317, found 316.0338 and 318.0316.
**2-(5-Bromo-1*H*-indol-3-yl)-2-phenylethan-1-ol (3p)**:yellow oil; yield: 220.6mg (70%), R_f_ = 0.42 (petroleum ether:ethyl aceate = 3:1). **^1^H NMR (400 MHz, DMSO-d6):**δ 11.06 (s, 1H), 7.41 (s, 1H), 7.29–7.20 (m, 6H), 7.12–7.06 (m, 2H), 4.72 (t, *J* = 4.8 Hz, 1H), 4.21 (t, *J* = 7.2 Hz, 1H), 4.00–3.86 (m, 2H). **^13^C NMR (100 MHz, DMSO-d6):**δ 148.7, 140.1, 134.1, 133.5, 133.3, 131.2, 129.2, 128.5, 126.1, 120.9, 118.6, 116.1, 70.5, 50.3. **IR (KBr):**3531, 3406, 2931, 1451, 1215, 1099, 798, 752, 700 cm^−1^. **HRMS (ESI):**calculated for C_16_H_15_BrNO ([M + H]^+^), *m*/*z* 316.0337 and 318.0317, found 316.0338 and 318.0323.
**2-(5-Iodo-1*H*-indol-3-yl)-2-phenylethan-1-ol (3q):**red solid; yield: 118.0 mg (65%), R_f_ = 0.37 (petroleum ether:ethyl aceate = 2:1), m.p. 170–171 °C. **^1^H NMR (400 MHz, CDCl_3_)**:δ 8.13 (br s, 1H), 7.77 (s, 1H), 7.42 (d, *J* = 8.6 Hz, 1H), 7.33–7.24 (m, 5H), 7.15–7.05 (m, 2H), 4.40 (t, *J* = 6.0 Hz, 1H), 4.22–4.10 (m, 2H), 1.57 (br s, 1H). **^13^C NMR (100 MHz, CDCl_3_):**δ 141.1, 135.5, 130.7, 129.7, 128.8, 128.2, 128.2, 127.0, 122.7, 115.7, 113.1, 83.1, 66.5, 45.4. **IR (KBr):**3456, 3363, 1633, 1450, 1334, 1215, 1097, 875, 702 cm^−1^. **HRMS (ESI):**calculated for C_16_H_15_INO ([M + H]^+^), *m*/*z* 364.0198, found 364.0201.
**3-(2-Hydroxy-1-phenylethyl)-1*H*-indole-5-carbonitrile (3r)**:white solid; yield: 63.0 mg (60%), R_f_ = 0.42 (petroleum ether:ethyl aceate = 3:1), m.p. 152–153 °C. **^1^H NMR (400 MHz, CDCl_3_)**:δ 8.50 (br s, 1H), 7.72 (s, 1H), 7.39 (s, 2H), 7.36–7.26 (m, 6H), 4.43 (t, *J* = 6.4 Hz, 1H), 4.25–4.15 (m, 2H), 1.64 (s, 1H). **^13^C NMR (100 MHz, CDCl_3_)**:δ 140.79, 138.08, 128.89, 128.22, 127.23, 126.98, 125.21, 123.96, 120.71, 117.35, 112.08, 102.66, 66.43, 45.32. **IR (KBr):**3533, 3450, 3026, 2227, 1618, 1325, 1221, 1043, 810, 700 cm^−1^. **HRMS (ESI):**calculated for C_17_H_15_N_2_O ([M + H]^+^), *m*/*z* 263.1184, found 263.1187.
**2-(5-Nitro-1*H*-indol-3-yl)-2-phenylethan-1-ol (3s):**yellow oil; yield: 56.5 mg (40%), R_f_ = 0.33 (petroleum ether:ethyl aceate = 1:1). **^1^H NMR (400 MHz, DMSO-*d*6)**:δ 11.69 (br s, 1H), 8.29 (s, 1H), 7.95 (d, *J* = 8.8 Hz, 1H), 7.58–7.48 (m, 2H), 7.36–7.27 (m, 4H), 7.19 (t, *J* = 7.2 Hz, 1H), 4.85 (s, 1H), 4.40 (t, *J* = 6.8 Hz, 1H), 4.08–3.93 (m, 2H). **^13^C NMR (100 MHz, DMSO-*d*6):**δ 143.5, 140.6, 139.8, 128.7, 128.7, 126.8, 126.8, 126.6, 119.3, 116.9, 116.3, 112.3, 65.7, 45.4. **IR (KBr):**3492, 3427, 1627, 1456, 1326, 1058, 740, 701 cm^−1^. **HRMS (ESI):**calculated for C_16_H_15_N_2_O_3_ ([M + H]^+^), *m*/*z* 283.1082, found 283.1083. **Chiral HPLC:**IB (isocratic, heptane: isopropanol) = 90:10, 0.5 mL min^−1^, UV at 254 nm, t(S) = 49.3 min, t(R) = 53.7 min. **(R)-2-(5-Nitro-1*H*-indol-3-yl)-2-phenylethan-1-ol (3s′)**:74% ee.
**Methyl 3-(2-hydroxy-1-phenylethyl)-1*H*-indole-4-carboxylate (3t):**green solid; yield: 73.8 mg (50%), R_f_ = 0.3 (petroleum ether:ethyl aceate = 3:1), m.p. 159–160 °C. **^1^H NMR (400 MHz, DMSO-*d*6):**δ 11.34 (br s, 1H), 7.56 (d, *J* = 8.0 Hz, 1H), 7.45 (s, 1H), 7.22–7.14 (m, 3H), 7.09–7.02 (m, 4H), 4.73–4.62 (m, 2H), 3.96–3.90 (m, 1H), 3.83–3.77 (m, 1H), 3.67 (s, 3H). **^13^C NMR (100 MHz, DMSO-*d*6):**δ 168.9, 144.5, 137.8, 129.0, 128.1, 126.0, 126.0, 124.7, 123.8, 121.2, 120.2, 116.1, 115.7, 66.4, 52.2, 45.6. **IR (KBr):**3485, 3392, 1687, 1436, 1282, 1043, 752, 700 cm^−1^. **HRMS (ESI):**calculated for C_18_H_17_NO_3_Na ([M + Na]^+^), *m*/*z* 318.1106, found 318.1110.
**Methyl 3-(2-hydroxy-1-phenylethyl)-1*H*-indole-6-carboxylate (3u):**green oil; yield: 84.2 mg (57%), R_f_ = 0.41 (petroleum ether:ethyl aceate = 3:1). **^1^H NMR (400 MHz, CDCl_3_)**:δ 8.48 (br s, 1H), 8.11 (s, 1H), 7.71 (d, *J* = 8.4 Hz, 1H), 7.43 (d, *J* = 8.4 Hz, 1H), 7.35–7.27 (m, 6H), 4.48 (s, 1H), 4.26–4.14 (m, 2H), 3.91 (s, 3H), 1.67 (br s, 1H). **^13^C NMR (100 MHz, CDCl_3_)**:δ 168.2, 141.3, 135.7, 130.6, 128.7, 128.3, 127.0, 125.4, 123.9, 120.5, 119.0, 116.5, 113.7, 66.5, 52.1, 45.4. **IR (KBr):**3496, 3388, 1695, 1436, 1280, 1207, 1143, 1039, 756, 698 cm^−1^. **HRMS (ESI):**calculated for C_18_H_18_NO_3_ ([M + H]^+^), *m*/*z* 296.1287, found 296.1290.
**Methyl 3-(2-hydroxy-1-phenylethyl)-1*H*-indole-7-carboxylate (3v):**white solid; yield: 88.6 mg (60%), R_f_ = 0.3 (petroleum ether:ethyl aceate = 3:1), m.p. 132–133 °C. **^1^H NMR (400 MHz, CDCl_3_)**:δ 9.76 (br s, 1H), 7.85 (d, *J* = 7.6 Hz, 1H), 7.63 (d, *J* = 7.8 Hz, 1H), 7.34–7.29 (m, 4H), 7.25 (s, 2H), 7.06 (t, *J* = 7.6 Hz, 1H), 4.48 (t, *J* = 6.8 Hz, 1H), 4.26–4.14 (m, 2H), 3.97 (s, 3H), 1.60 (br s, 1H). **^13^C NMR (100 MHz, CDCl_3_)**:δ 167.9, 141.5, 136.4, 128.7, 128.3, 128.3, 126.9, 125.1, 124.6, 122.9, 118.9, 116.1, 112.5, 66.6, 51.0, 45.5. **IR (KBr):**3496, 3388, 1695, 1436, 1280, 1143, 1039, 756, 698 cm^−1^. **HRMS (ESI):**calculated for C_18_H_18_NO_3_ ([M + H]^+^), *m*/*z* 296.1287, found 296.1288.
**3-(4-Bromophenyl)-2-(1*H*-indol-3-yl)ethan-1-ol (3aa):**yellow oil; yield: 139.1 mg (88%), R_f_ = 0.32 (petroleum ether:ethyl aceate = 3:1). **^1^H NMR (400 MHz, CDCl_3_)**:δ 8.14 (br s, 1H), 7.38 (d, *J* = 8.4 Hz, 3H), 7.29 (d, *J* = 8.4 Hz, 1H), 7.18–7.13 (m, 3H), 7.03 (t, J = 7.6 Hz, 1H), 6.97 (s, 1H), 4.37 (t, J = 6.8 Hz, 1H), 4.18–4.13 (m, 2H), 4.10–4.03 (m, 2H), 1.81 (br s, 1H). **^13^C NMR (100 MHz, CDCl_3_)**:δ 140.9, 136.5, 131.7, 130.2, 126.8, 122.5, 122.1, 120.6, 119.7, 119.3, 115.4, 111.4, 66.3, 45.1. **IR (KBr):**3415, 3303, 2927, 1487, 1338, 1222, 1103, 1037, 815, 742, 700 cm^−1^. **HRMS (ESI):**calculated for C_16_H_15_BrNO ([M + H]^+^), *m*/*z* 316.0337 and 318.0317, found 316.0340 and 318.0323.
**2-(4-Bromophenyl)-2-(7-methyl-1*H*-indol-3-yl)ethan-1-ol (3ab):**yellow oil; yield: 138.7 mg (84%), R_f_ = 0.32 (petroleum ether:ethyl aceate = 3:1). **^1^H NMR (400 MHz, CDCl_3_):**δ 8.05 (br s, 1H), 7.41 (d, *J* = 8.4 Hz, 2H), 7.25–7.20 (m, 3H), 7.10 (d, *J* = 2.4 Hz, 1H), 7.01–6.97 (m, 2H), 4.43 (t, J = 6.8 Hz, 1H), 4.25–4.19 (m, 1H), 4.16–4.10 (m, 1H), 2.48 (s, 3H), 1.59 (br s, 1H). **^13^C NMR (100 MHz, CDCl_3_)**:δ 140.7, 135.9, 131.5, 129.9, 126.2, 122.9, 121.5, 120.4, 120.3, 119.8, 116.8, 115.8, 66.0, 45.0, 16.5. **IR (KBr):**3411, 3301, 2923, 1612, 1485, 1226, 1068, 784, 748 cm^−1^. **HRMS (ESI):**calculated for C_17_H_17_BrNO ([M + H]^+^), *m*/*z* 330.0494 and 332.0474, found 330.0496 and 332.0480.
**3-(4-Bromophenyl)-2-(5-nitro-1*H*-indol-3-yl)ethan-1-ol (3ac):**yellow oil; yield: 72.2 mg (40%), R_f_ = 0.32 (petroleum ether:ethyl aceate = 3:1), m.p. 205–206 °C. **1H NMR (400 MHz, DMSO-*d*6)**:δ 11.75 (br s, 1H), 8.32 (s, 1H), 7.97 (dd, *J* = 8.8, 2 Hz, 1H), 7.58 (s, 1H), 7.53–7.47 (m, 3H), 7.32 (d, *J* = 8.0 Hz, 2H), 4.93 (t, *J* = 5.2 Hz, 1H), 4.42 (t, *J* = 6.8 Hz, 1H), 4.05–3.94 (m, 2H). **^13^C NMR (100 MHz, DMSO-*d*6):**δ 143.1, 140.6, 139.8, 131.5, 131.0, 127.0, 126.6, 119.7, 118.7, 117.0, 116.2, 112.4, 65.4, 44.6. **IR (KBr):**3294, 2920, 2867, 1620, 1475, 1325, 1010, 810, 738 cm^−1^. **HRMS (ESI):**calculated for C_16_H_14_BrN_2_O_3_ ([M + H]^+^), *m*/*z* 361.0188 and 363.0168, found 361.0189 and 363.0169.
**2-(1*H*-indol-3-yl)-2-(m-tolyl)ethan-1-ol (3ad)**:yellow oil; yield: 88.0 mg (70%), R_f_ = 0.4 (petroleum ether:ethyl aceate = 3:1). **^1^H NMR (400 MHz, CDCl_3_):**δ 8.24 (s, 1H), 7.59 (d, *J* = 8.0 Hz, 1H), 7.41 (d, *J* = 8.4 Hz, 1H), 7.33–7.24 (m, 4H), 7.19–7.10 (m, 3H), 4.54 (t, *J* = 6.8 Hz, 1H), 4.34–4.22 (m, 2H), 2.42 (s, 3H), 1.92 (br s, 1H). **^13^C NMR (100 MHz, CDCl_3_):**δ 141.7, 138.3, 136.5, 129.2, 128.6, 127.6, 127.1, 125.4, 122.3, 122.0, 119.6, 119.4, 116.0, 111.3, 66.5, 45.6, 21.6. **IR (KBr):**3470, 3048, 2921, 1632, 1490, 1030, 815, 700 cm^−1^. **HRMS (ESI):**calculated for C_17_H_18_NO ([M + H]^+^), *m*/*z* 252.1388, found 252.1388.
**3-(2-Hydroxy-1-(1*H*-indol-3-yl)ethyl)benzonitrile (3ae):**white solid; yield: 108.9 mg (83%), R_f_ = 0.3 (petroleum ether:ethyl aceate = 3:1). **^1^H NMR (400 MHz, CDCl_3_):**δ 8.23 (br s, 1H), 7.65–7.58 (m, 2H), 7.51 (d, *J* = 7.8 Hz, 1H), 7.43–7-35 (m, 3H), 7.23–7.18 (m, 1H), 7.14 (s, 1H), 7.07 (t, *J* = 7.6 Hz, 1H), 4.51 (t, J = 6.7 Hz, 1H), 4.28–4.14 (m, 2H), 1.64 (br s, 1H). **^13^C NMR (100 MHz, CDCl_3_):**δ 143.7, 136.5, 133.0, 131.9, 130.5, 129.3, 126.6, 122.7, 122.1, 119.9, 119.0, 114.8, 112.5, 111.4, 65.9, 45.1. **IR (KBr):**3550, 3415, 3048, 2226, 1630, 1490, 1032, 835, 700 cm^−1^. **HRMS (ESI):**calculated for C_17_H_15_N_2_O ([M + H]^+^), *m*/*z* 263.1184, found 263.1187.
**2-(1*H*-indol-3-yl)-2-(2-methoxyphenyl)ethan-1-ol (3af):**yellow solid; yield: 106.9 mg (80%), R_f_ = 0.42 (petroleum ether:ethyl aceate = 2:1), m.p. 115–116 °C. **^1^H NMR (600 MHz, CDCl_3_):**δ 8.11 (brs, 1H), 7.48 (d, *J* = 8.8 Hz, 1H), 7.35 (d, *J* = 8.2 Hz, 1H), 7.23–7.15 (m, 4H), 7.05 (t, *J* = 8.0 Hz, 1H), 6.93 (d, *J* = 8.3 Hz, 1H), 6.85 (t, *J* = 7.4 Hz, 1H), 5.02 (t, *J* = 6.6 Hz, 1H), 4.24–4.15 (m, 2H), 3.89 (s, 3H). **^13^C NMR (150 MHz, CDCl_3_):**δ157.3, 136.5, 129.8, 128.9, 127.7, 127.3, 122.2, 122.2, 120.7, 119.5, 119.4, 116.1, 111.1, 110.7, 65.7, 55.6, 38.1. **IR (KBr):**3550, 3409, 2935, 1596, 1456, 1103, 1026, 750, 738 cm^−1^. **HRMS (ESI):**calculated for C_16_H_15_INO ([M + H]^+^), *m*/*z* 268.1338, found 268.1342.
**2-(1*H*-indol-3-yl)-2,2-diphenylethan-1-ol (3ag):**white solid; yield: 98.7 mg (63%), Rf = 0.35 (petroleum ether:ethyl aceate = 3:1), m.p. 181–182 °C. **^1^H NMR (600 MHz, CDCl_3_):**δ8.10 (brs, 1H), 7.36–7.30 (m, 5H), 7.29–7.24 (m, 4H), 7.23–7.20 (m, 2H), 7.17–7.14 (m, 1H), 7.09 (d, *J* = 7.8 Hz, 1H), 6.93 (t, *J* = 7.0 Hz, 1H), 6.74 (d, *J* = 2.5 Hz, 1H), 4.68 (s, 2H). 1.58 (br s, 1H). **^13^C NMR (151 MHz, CDCl_3_):**δ 145.1, 137.0, 129.0, 128.2, 126.5, 126.3, 122.2, 121.6, 119.6, 119.5, 111.4, 69.3, 55.0. **IR (KBr):**3560, 3280, 2900, 1595, 1444, 1336, 1240, 1000, 736, 702 cm^−1^. **HRMS (ESI):**calculated for C_22_H_19_NO ([M + H]^+^), *m*/*z* 314.1545, found 314.1547.
**1-(1*H*-indol-3-yl)-3-phenoxypropan-2-ol (3ah):**yellow oil; yield: 69.4 mg (52%), R_f_ = 0.4 (petroleum ether:ethyl aceate = 3:1). **^1^H NMR (400 MHz, CDCl_3_):**δ 8.03 (br s, 1H), 7.62 (d, *J* = 7.6 Hz, 1H), 7.32 (d, *J* = 7.6 Hz, 1H), 7.27–7.18 (m, 4H), 7.13–7.09 (m, 1H), 6.94 (t, *J* = 7.6 Hz, 1H), 6.88 (d, J = 8.4 Hz, 1H), 4.36–4.31 (m, 1H), 4.00–3.92 (m, 2H), 3.15–3.02 (m, 2H), 2.37 (br s, 1H). **^13^C NMR (100 MHz, CDCl_3_):**δ 158.7, 136.4, 129.6, 127.7, 123.0, 122.3, 121.1, 119.6, 118.9, 114.7, 111.4, 111.3, 71.3, 70.2, 29.5. **IR (KBr):**3411, 3047, 2916, 1596, 1454, 1242, 1097, 746, 700 cm^−1^. **HRMS (ESI):**calculated for C_17_H_18_NO_2_ ([M + H]^+^), *m*/*z* 268.1338, found 268.1335.
**1-Chloro-3-(1*H*-indol-3-yl)propan-2-ol (3ai):**yellow oil; yield: 63.4 mg (55%), R_f_ = 0.32 (petroleum ether:ethyl aceate = 3:1). **^1^H NMR (400 MHz, CDCl_3_):**δ 8.09 (s, 1H), 7.66 (d, *J* = 8.0 Hz, 1H), 7.40 (d, *J* = 8.0 Hz, 1H), 7.23 (d, *J* = 8.0 Hz, 1H), 7.19–7.12 (m, 2H), 4.19 (s, 1H), 3.76–3.51 (m, 2H), 3.13–3.04 (m, 2H), 2.24 (br s, 1H). **^13^C NMR (100 MHz, CDCl_3_):**δ 136.4, 127.5, 123.0, 122.4, 119.8, 118.8, 111.3, 111.0, 71.3, 49.3, 30.2. **IR (KBr):**3417, 3053, 2918, 1622, 1458, 1338, 1087, 742, 702 cm^−1^. **HRMS (ESI):**calculated for C_11_H_13_ClNO ([M + H]^+^), *m*/*z* 210.0686 and 212.0657, found 210.0690 and 212.0663.
**3-(1*H*-indol-3-yl)-3-phenylpropan-1-ol (5)**:yellow solid; yield: 69.1 mg (55%), R_f_ = 0.43 (petroleum ether:ethyl aceate = 3:1), m.p. 99–100 °C. **^1^H NMR (400 MHz, CDCl_3_):**δ 8.00 (br s, 1H), 7.45 (d, *J* = 8.0 Hz, 1H), 7.33–7.24 (m, 5H), 7.18–7.12 (m, 2H), 7.06–6.99 (m, 2H), 4.39 (t, *J* = 7.6 Hz, 1H), 3.69–3.63 (m, 2H), 2.52–2.42 (m, 1H), 2.33–2.22 (m, 1H), 1.58 (br s, 1H). **^13^C NMR (100 MHz, CDCl_3_):**δ 144.8, 136.6, 128.5, 127.9, 126.9, 126.2, 122.1, 121.1, 119.8, 119.6, 119.4, 111.1, 61.41, 39.3, 38.7. **IR (KBr):**3411, 3386, 2865, 1614, 1452, 1048, 1006, 742, 696 cm^−1^. **HRMS (ESI):**calculated for C_17_H_18_NO ([M + H]^+^), *m*/*z* 252.1388, found 252.1391.

## 4. Conclusions

In summary, a highly efficient, mild, and strictly catalyst-free protocol for the ring-opening of cyclic ethers with indoles has been developed, using HFIP as the sole solvent and promoter. The reaction proceeds at room temperature within 6 h, affording a diverse array of C3-alkylated indoles in good-to-excellent yields. Importantly, this method successfully accommodates both epoxides and the more challenging four-membered oxetanes, while stereospecific studies confirm an S_N_2 pathway with excellent enantioselectivity. This green, atom-economical, and operationally simple strategy offers a practical alternative to conventional acid-catalyzed approaches, and its scalability and functional-group compatibility highlight its potential for broader synthetic applications.

## Figures and Tables

**Table 1:** Optimization of the Reaction Conditions ^[a]^.

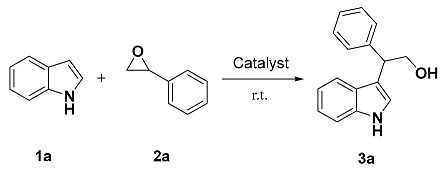							
Entry	Catalyst	Catalyst (mol%)	Solvent	C (M)	T (h)	1a (equiv.)	Yield of 3a (%) ^[b]^
1	TFA	5	HFIP	0.5	6	5	81
2	HCl	5	HFIP	0.5	6	5	69
3	TfOH	5	HFIP	0.5	6	5	88
4	In(OTf)_3_	5	HFIP	0.5	6	5	82
5	Sc(OTf)_3_	5	HFIP	0.5	6	5	86
6	-	-	HFIP	0.5	6	5	84
7	-	-	HFIP	0.5	6	1	61
8	-	-	HFIP	0.5	6	2	78
9	-	-	HFIP	0.5	6	3	78
10	-	-	HFIP	0.5	6	4	89, 82 ^[c]^
11			HFIP	0.5	5	4	78
12			HFIP	0.5	3	4	52
13	-	-	HFIP	1	6	4	75
14	-	-	HFIP	0.25	6	4	82
15	-	-	EtOH	0.5	6	4	NR ^[d]^
16	-	-	MeCN	0.5	6	4	NR
17	-	-	Toluene	0.5	6	4	NR
18	-	-	*i*-PrOH	0.5	6	4	NR
19	-	-	TFE	0.5	6	4	NR
20	-	-	TFE	0.5	24	4	trace
21	-	-	HFIP, DCM	0.5 (1:19)	6	4	NR
22	-	-	HFIP, DCM	0.5 (1:9)	6	4	NR
23	-	-	HFIP, DCM	0.5 (1:4)	6	4	trace
24	-	-	HFIP, EtOH	0.5 (1:1)	6	4	63

^[a]^ Reaction conditions: **2a** (0.5 mmol) at room temperature. ^[b]^ Yield was determined by ^1^H NMR, using 1,1,2,2-tetrachloroethane as an internal standard. ^[c]^ Yield of the isolated product. ^[d]^ No reaction.

**Table 2:** Substrate scope of indoles ^[a]^.

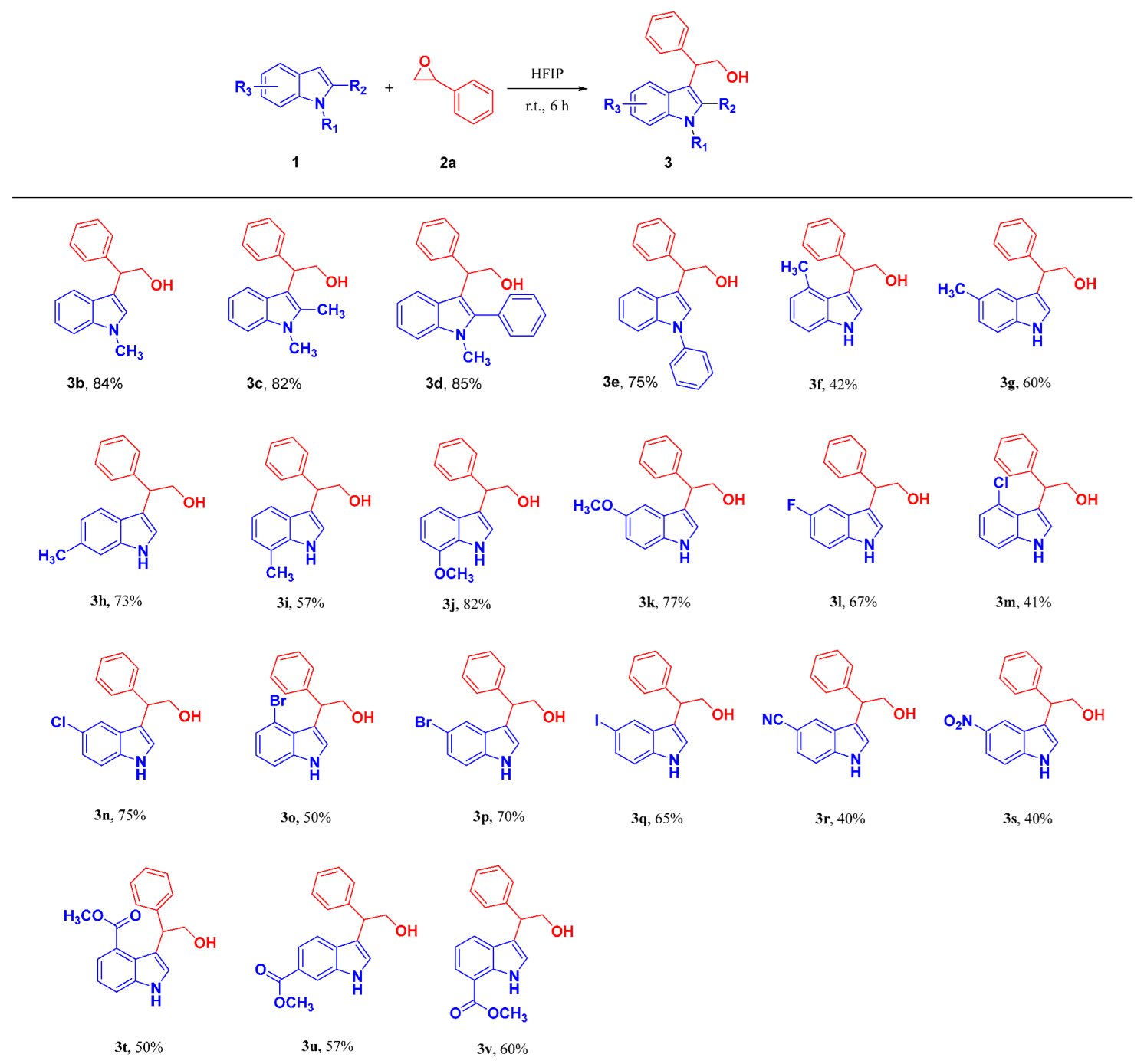

^[a]^ Reaction conditions: **1** (2 mmol) and **2a** (0.5 mmol) in HFIP (1 mL) at room temperature for 6 h; all are isolated yields.

**Table 3:** Substrate scope of epoxides ^[a]^.

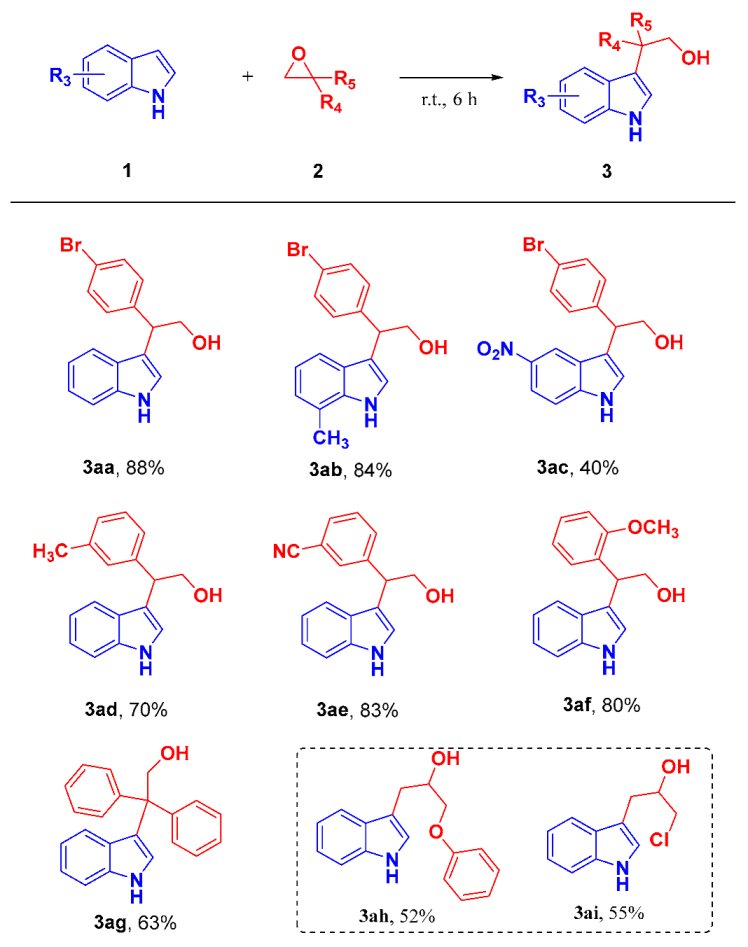

^[a]^ Reaction conditions: **1** (2 mmol) and **2** (0.5 mmol) in HFIP (1 mL) at room temperature for 6 h; all are isolated yields.

## Data Availability

All data supporting the findings of this study are available within the paper and within its Supplementary Materials published online.

## Supplementary Materials

The following supporting information can be downloaded at https://www.mdpi.com/article/10.3390/molecules31173108/s1, which contains details on the experimental procedure for compounds **2b**–**2e**, experimental procedure for compound **4**, ^1^H, ^13^C, ^19^F NMR spectra and HPLC spectra.

## References

[1] Silva Moratorio de Moraes R., Mestre Botelho A.B., Tavares de Almeida Pinto G., Couto Rodrigues S., Miranda Martins M.T., Alves Soares D.L., Cardoso Cruz C., de Almeida Pinto A., Rodrigues Fintelman Dias F., Dias Fernandes P. (2025). Chemistry, Applications, and Synthesis Methods of Indole Derivatives: A Comprehensive Review. Chem. Rec..

[2] Kilic H., Bayindir S., Erdogan E., Saracoglu N. (2012). Synthesis of highly N-substituted indole library via conjugate additions of indoline and their synthetic tool potentials. Tetrahedron.

[3] Kumar P., Lee J.H., Lee J. (2021). Diverse roles of microbial indole compounds in eukaryotic systems. Biol. Rev. Camb. Philos. Soc..

[4] Moreira V.M., Salvador J.A., Beja A.M., Paixao J.A. (2011). The reaction of azoles with 17-chloro-16-formylandrosta-5,16-dien-3beta-yl-acetate: Synthesis and structural elucidation of novel 16-azolylmethylene-17-oxoandrostanes. Steroids.

[5] Rallabandi J., Mohanty S., Shown I. (2024). Ruthenium(ii) catalyzed C-3 site selective alkenylation of indole derivatives via C-H activation. RSC Adv..

[6] Belasri K., Fulop F., Szatmari I. (2019). Solvent-Free C-3 Coupling of Azaindoles with Cyclic Imines. Molecules.

[7] Song H., Zhou H., Shen Y., Wang H., Song H., Cai X., Xu C. (2022). HFIP as Protonation Reagent and Solvent for Regioselective Alkylation of Indoles with All-Carbon Centers. J. Org. Chem..

[8] Rashid A., Lone W.I., Dogra P., Rashid S., Bhat B.A. (2024). HFIP-mediated C-3-alkylation of indoles and synthesis of indolo[2,3-b]quinolines & related natural products. Org. Biomol. Chem..

[9] Basak S., Alvarez-Montoya A., Winfrey L., Melen R.L., Morrill L.C., Pulis A.P. (2020). B(C_6_F_5_)_3_-Catalyzed Direct C3 Alkylation of Indoles and Oxindoles. ACS Catal..

[10] Mazzotta S., Frattaruolo L., Brindisi M., Ulivieri C., Vanni F., Brizzi A., Carullo G., Cappello A.R., Aiello F. (2020). 3-Amino-alkylated Indoles: Unexplored Green Products Acting As anti-inflammatory Agents. Future Med. Chem..

[11] Arjunan V., Durgadevi G., Mohan S. (2020). An experimental and theoretical investigation on the structure, vibrations and reactivity properties of pharmacologically active compounds 3-acetylindole and indole-3-acetamide. J. Mol. Struct..

[12] Cadelis M.M., Li S.A., Bourguet-Kondracki M.L., Blanchet M., Douafer H., Brunel J.M., Copp B.R. (2021). Spermine Derivatives of Indole-3-carboxylic Acid, Indole-3-acetic Acid and Indole-3-acrylic Acid as Gram-Negative Antibiotic Adjuvants. ChemMedChem.

[13] Mei H., Pan G., Zhang X., Lin L., Liu X., Feng X. (2018). Catalytic Asymmetric Ring-Opening/Cyclopropanation of Cyclic Sulfur Ylides: Construction of Sulfur-Containing Spirocyclopropyloxindoles with Three Vicinal Stereocenters. Org. Lett..

[14] Mishra D.R., Mishra N.P. (2025). Recent breakthroughs in ring-opening annulation reactions of aziridines. Org. Biomol. Chem..

[15] Yadav J.S., Reddy B.V.S., Abraham S., Sabitha G. (2002). InCl3-Catalyzed Alkylation of Indoles with Epoxides. Synlett.

[16] Yadav J.S., Reddy B.V.S., Parimala G. (2002). Indium Tribromide Catalyzed Highly Regioselective Ring Opening of Epoxides and Aziridines with Pyrrole. Synlett.

[17] Bandini M., Fagioli M., Melloni A., Umani-Ronchi A. (2004). Polymer-Supported Indium Lewis Acid: Highly Versatile Catalyst for Regio- and Stereoselective Ring-Opening of Epoxides. Adv. Synth. Catal..

[18] Nagarjun N., Concepcion P., Dhakshinamoorthy A. (2020). MIL-101(Fe) as an active heterogeneous solid acid catalyst for the regioselective ring opening of epoxides by indoles. Mol. Catal..

[19] Nasef M.M., Zakeri M., Asadi J., Abouzari-Lotf E., Ahmad A., Malakooti R. (2016). Environmentally benign and highly regioselective ring opening of epoxides accelerated by ultrasound irradiation. Green Chem. Lett. Rev..

[20] Gu Y.C., Hu R.M., Li M.M., Xu D.Z. (2019). Iron-containing ionic liquid as an efficient and recyclable catalyst for the synthesis of C3-substituted indole derivatives. Appl. Organomet. Chem..

[21] Acocella M.R., Mauro M., Guerra G. (2014). Regio- and enantioselective friedel-crafts reactions of indoles to epoxides catalyzed by graphene oxide: A green approach. ChemSusChem.

[22] Das U., Crousse B., Kesavan K., Bonnet-Delpon D., Bégué J. (2000). Facile Ring Opening of Oxiranes with Aromatic Amines in Fluoro Alcohols. J. Org. Chem..

[23] Pozhydaiev V., Power M., Gandon V., Moran J., Leboeuf D. (2020). Exploiting hexafluoroisopropanol (HFIP) in Lewis and Bronsted acid-catalyzed reactions. Chem. Commun..

[24] Ameduri B. (2009). From Vinylidene Fluoride (VDF) to the Applications of VDF-Containing Polymers and Copolymers: Recent Developments and Future Trends. Chem. Rev..

[25] Zhang S., Vayer M., Noël F., Vuković V.D., Golushko A., Rezajooei N., Rowley C.N., Lebœuf D., Moran J. (2021). Unlocking the Friedel-Crafts arylation of primary aliphatic alcohols and epoxides driven by hexafluoroisopropanol. Chem.

[26] Vayer M., Zhang S., Moran J., Lebœuf D. (2022). Rapid and Mild Metal-Free Reduction of Epoxides to Primary Alcohols Mediated by HFIP. ACS Catal..

[27] Yaragorla S., Tangellapally R., Arun D. (2024). Hexafluoroisopropanol Promoted Ring-Opening-Cyclization of Donor-Acceptor Cyclopropanes with Primary Amines. Eur. J. Org. Chem..

[28] Deswal S., Biju A.T. (2026). HFIP-Mediated (3+2) Annulation of Bicyclobutanes with Indolyl Alcohols for Diastereoselective Access to Tetracyclic Spiroindolenines. Org. Lett..

[29] Sharma V.P., Nidhar M., Sheraj M., Kumar V., Sonker P., Patel A., Gill S., Sura S., Tewari A.K. (2024). Ultrasound-assisted ring opening of epoxides in HFIP: THF: Synthesis, characterization, computational studies and molecular docking of novel 2-hydroxy dithiocarbamates. J. Mol. Struct..

[30] Kirby G., Prestat G., Berhal F. (2023). Iron-Catalyzed Intermolecular Oxyamination of Terminal Alkenes Promoted by HFIP Using Hydroxylamine Derivatives. J. Org. Chem..

[31] Richmond E., Yi J., Vukovic V.D., Sajadi F., Rowley C.N., Moran J. (2018). Ring-opening hydroarylation of monosubstituted cyclopropanes enabled by hexafluoroisopropanol. Chem. Sci..

[32] Clerc M., Stricker F., Ulrich S., Sroda M., Bruns N., Boesel L.F., Read de Alaniz J. (2021). Promoting the Furan Ring-Opening Reaction to Access New Donor-Acceptor Stenhouse Adducts with Hexafluoroisopropanol. Angew. Chem. Int. Ed. Engl..

[33] Iskra J., Bonnet-Delpon D., Bégué J.-P. (2005). Comparative study of the ring opening of 1-CF3-epoxy ethers mediated by Brönsted acids and hexafluoro-2-propanol. J. Fluor. Chem..

[34] Zou S., Zhang Z., Chen C., Xi C. (2021). MeOTf/KI-catalyzed efficient synthesis of 2-arylnaphthalenes via cyclodimerization of styrene oxides. Org. Biomol. Chem..

[35] Tyagi A., Khan J., Yadav N., Mahato R., Hazra C.K. (2022). Catalyst-Switchable Divergent Synthesis of Bis(indolyl)alkanes and 3-Alkylated Indoles from Styrene Oxides. J. Org. Chem..

[36] Jana S., Yang Z., Pei C., Xu X., Koenigs R.M. (2019). Photochemical ring expansion reactions: Synthesis of tetrahydrofuran derivatives and mechanism studies. Chem. Sci..

