# Supplementary material for: Hexafluoroisopropanol Mediated Ring-Opening Reactions of Epoxides with Indoles Under Catalyst-Free Conditions
Source: Molecules. 2026 Sep 4;31(17):3108. doi: 10.3390/molecules31173108 (PMC13567142; doi:10.3390/molecules31173108)
Supplement: Supplementary file 1 [file molecules-31-03108-s001.zip › molecules-4487649-supplementary.pdf]

Supporting Information

Hexafluoroisopropanol Mediated Ring-Opening Reactions of Epoxides with Indoles under Catalyst-Free Conditions

Yangmin Ma *, Ding Ma, Zhichao Wang *, Murong Zhan, Qian Jiao and Sen Yang

Key Laboratory of Chemical Additives, China National Light Industry, College of Chemistry and Chemical Engineering, Shaanxi University of Science and Technology, Xi'an 710021, China

* Correspondence: mayangmin@sust.edu.cn (Y. M.); 5264@sust.edu.cn (Z. W.)

Table of Contents

1. General Information..1

2. Synthesis of Oxide of Styrene Oxide...2

3. Synthesis of 2-Phenyloxetane..3

4. NMR spectra...4

5. HPLC traces...37

1. General Information

^{1}H NMR spectra were recorded on a Bruker Ascend 400 (400 MHz) and 600 (600 MHz) spectrometer. Data for ^{1}H NMR are reported as follows: chemical shifts in ppm relative to tetramethylsilane as an internal standard in $CDCl_3$, integration, multiplicity (s = singlet, d = doublet, t = triplet, q = quartet, m = multiplet, br = broad), coupling constants (Hz), and assignment. ^{13}C NMR spectra were recorded on a Bruker Ascend 400 (100 MHz) and 600 (150 MHz) spectrometer with complete proton decoupling. Chemical shifts for ^{13}C NMR were reported in ppm relative to the residual solvent as an internal standard. ^{19}F NMR spectra were acquired at 376 MHz on a Bruker Ascend 400 spectrometer. High resolution mass spectra were acquired on a Waters G2-S Qtof mass spectrometer. All reagents and solvents were purchased from commercial sources and used without further purification.

All commercially available chemicals and solvents were used directly without further purification unless otherwise stated. Specifically, all indole starting materials were purchased from Energy Chemical (Anhui, China) and Macklin (Shanghai, China) with a purity of >99%. Hexafluoroisopropanol (HFIP, purity: 99.5%) was also purchased from Energy Chemical (Anhui, China). Most terminal epoxides used in the substrate scope were commercially available and used as received.

2. Synthesis of Oxide of Styrene Oxide

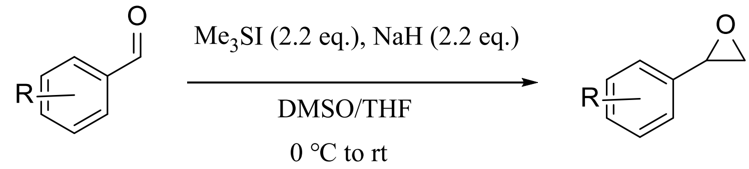

6 **2**

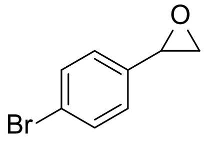

2b, 69%

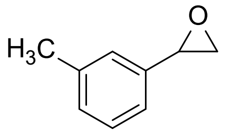

2c, 73%

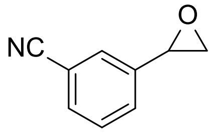

2d, 63%

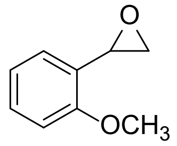

2e, 67%

A solution of NaH (11 mmol, 2.2 equiv.) and Me_3SI (11 mmol, 2.2 equiv.) in DMSO (7 mL) and THF (6 mL) was stirred for 30 min at room temperature in a 50 mL flask under an Ar atmosphere. Then a solution of aldehyde **6** (5 mmol,1.0 equiv.) in THF was added dropwise at 0 °C. After complete addition, the mixture was warmed to room temperature and allowed to stir for 12 hours. The mixture was quenched with sat. NH_4Cl and diluted with EtOAc. Then the mixture was separated and the aqueous layer was back-extracted with EtOAc. The organic layer was washed with brine and dried over Na_2SO_4. The solvent was removed under reduced pressure and the residue was purified by column chromatography to give the desired epoxides.

3. Synthesis of 2-Phenyloxetane

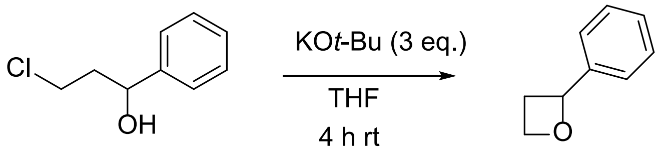

7

4, 56%

3-Chloro-1-phenyl-1-propanone **7** (5 mmol, 1.0 equiv) was dissolved in anhydrous THF (40 mL), and KO*t*-Bu (15 mmol, 3.0 equiv) was added. The mixture was stirred at room temperature for 4 hours. Upon completion of the reaction, water was added to quench the reaction, and the mixture was diluted with ethyl acetate. The resulting mixture was transferred to a separatory funnel, and the layers were separated after thorough shaking and standing. The aqueous layer was extracted twice with ethyl acetate. The combined organic layers were washed successively with saturated brine and dried over anhydrous sodium sulfate. After filtration, the solvent was removed under reduced pressure, and the crude product was purified by column chromatography on silica gel to afford the desired epoxide compound.

4. NMR spectra

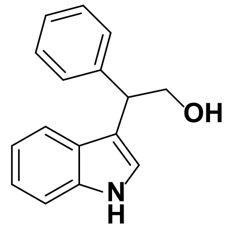

3a

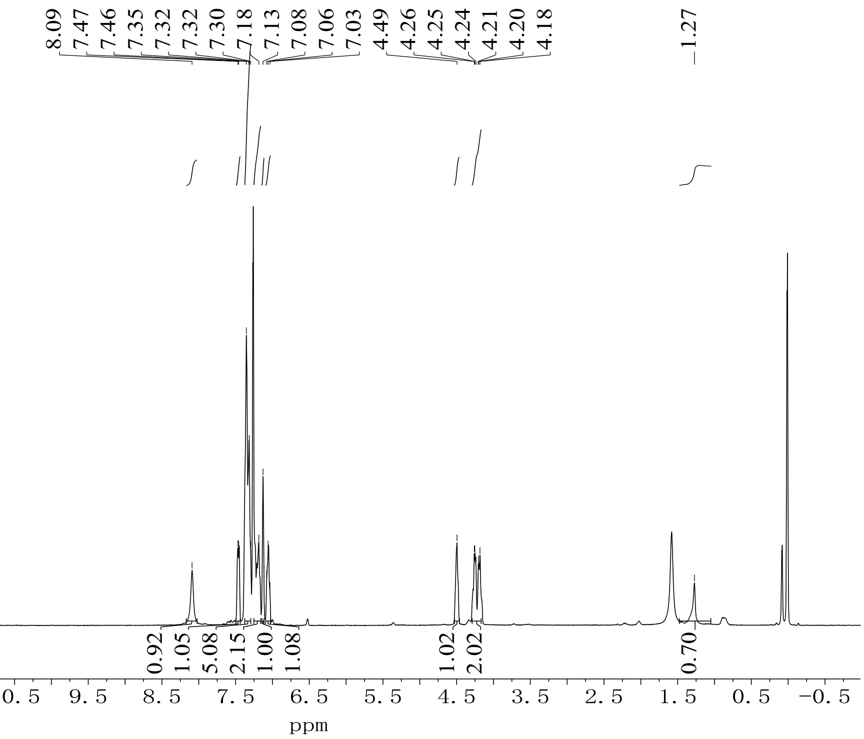

^{1}H NMR spectrum of compound **3a** (400 MHz, CDCl$_3$, 298 K)

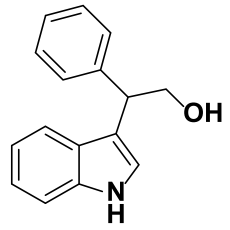

3a

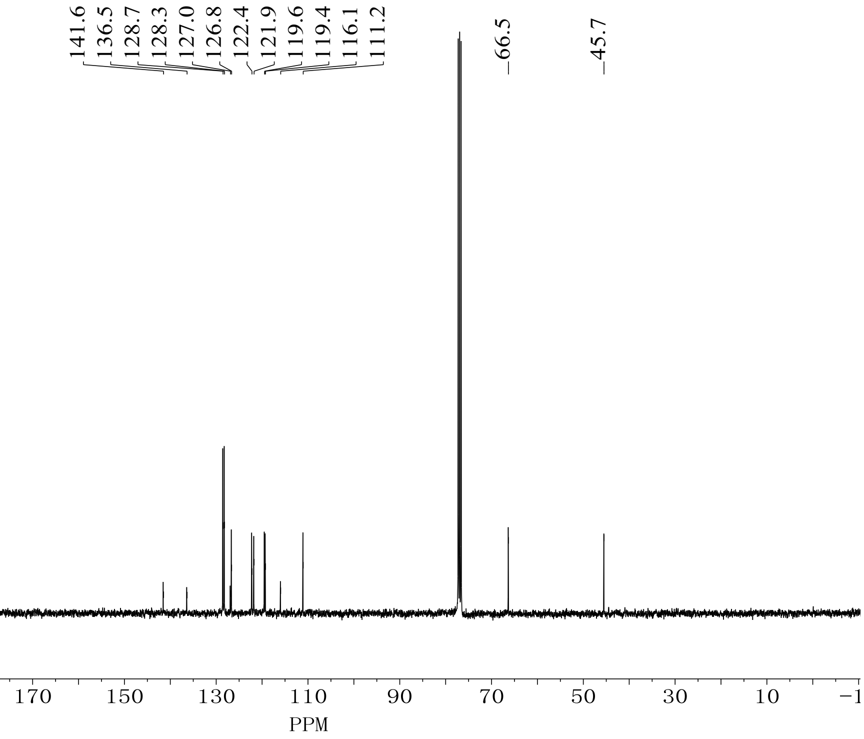

^{13}C NMR spectrum of compound **3a** (100 MHz, CDCl$_3$, 298 K)

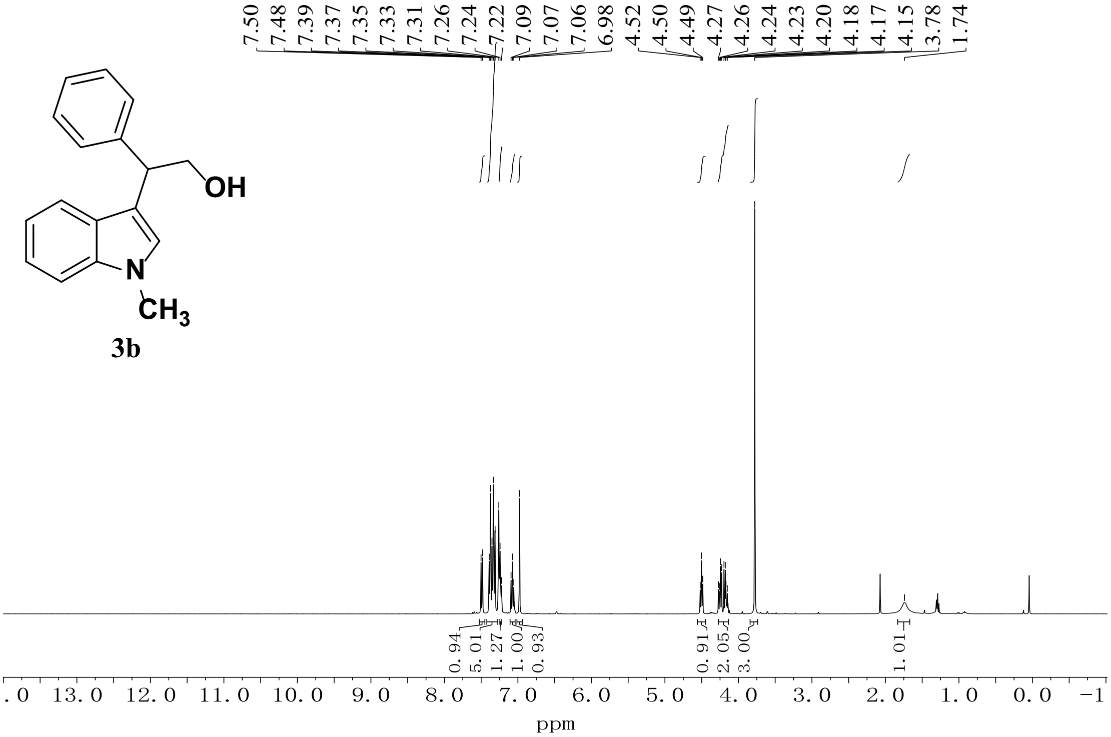

^{1}H NMR spectrum of compound **3b** (400 MHz, $CDCl_3$, 298 K)

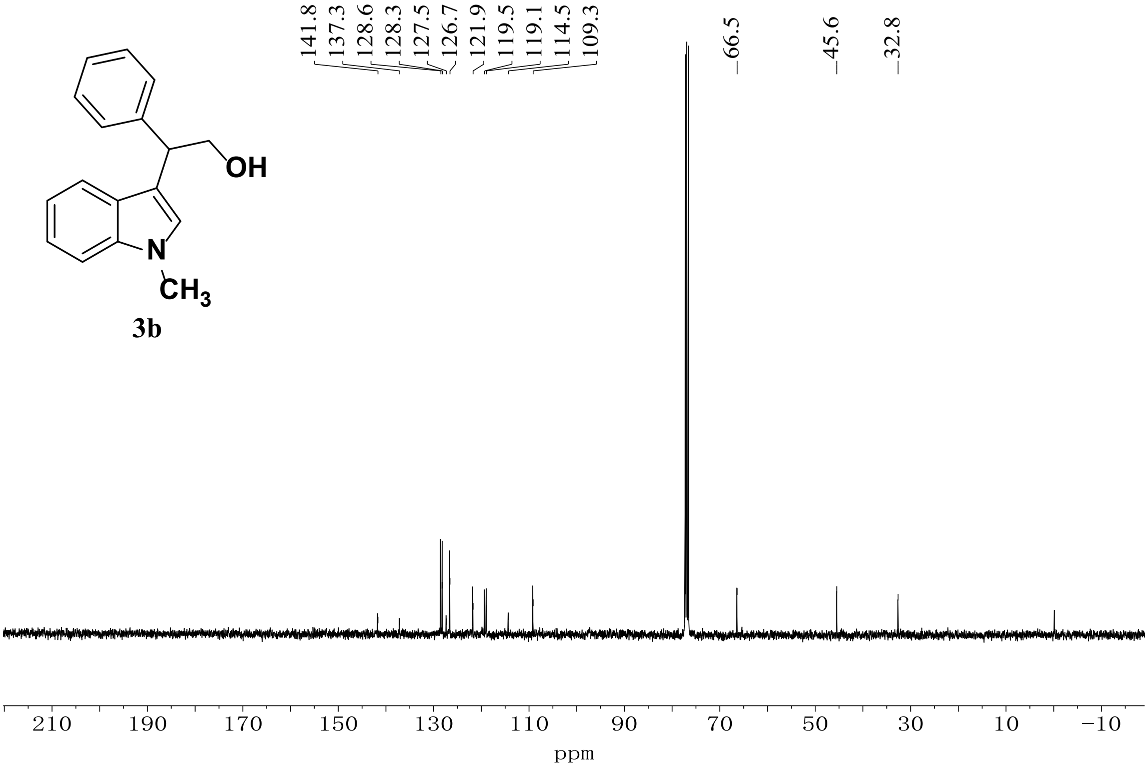

^{13}C NMR spectrum of compound **3b** (100 MHz, $CDCl_3$, 298 K)

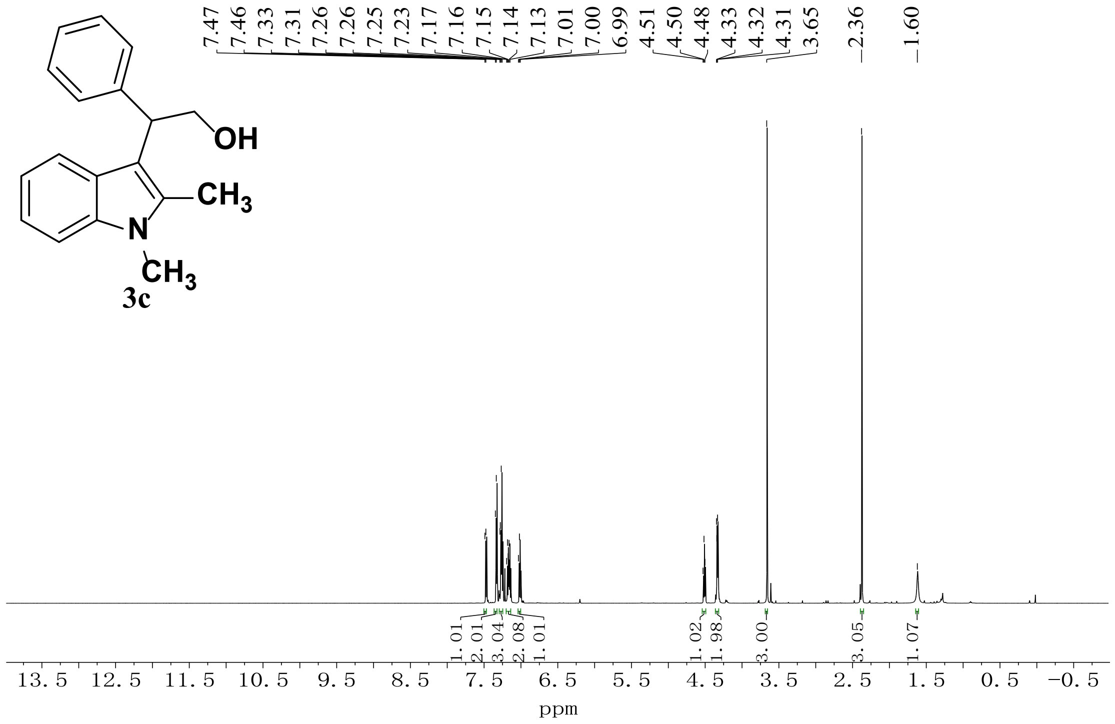

^{1}H NMR spectrum of compound **3c** (600 MHz, $CDCl_3$, 298 K)

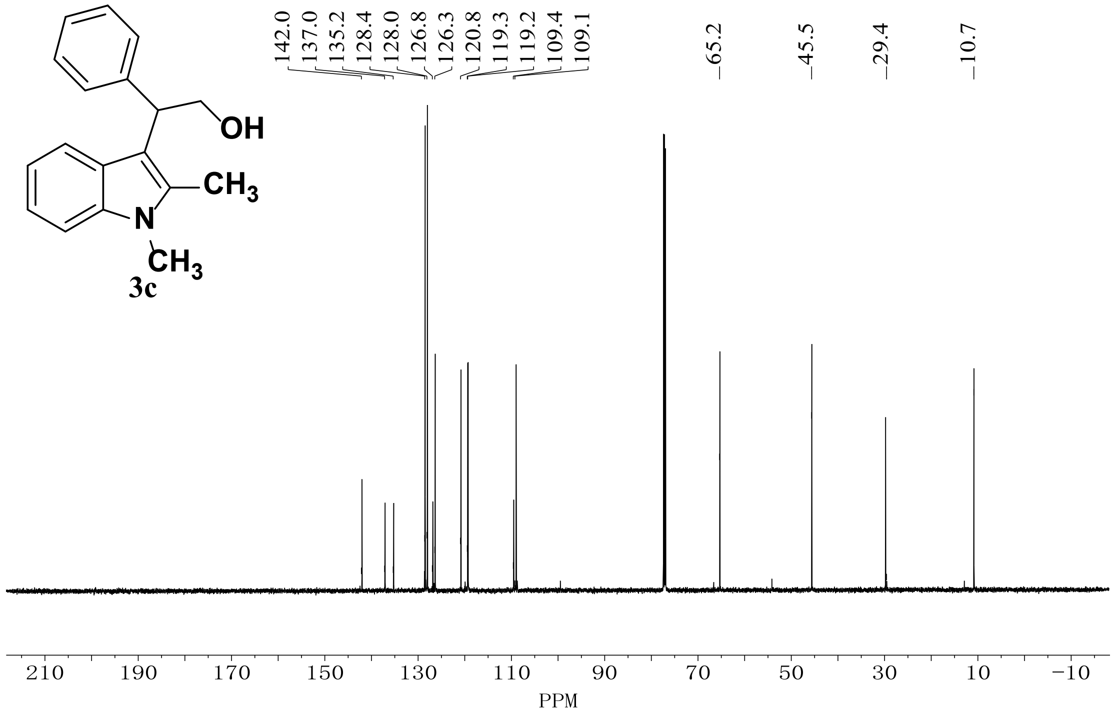

^{13}C NMR spectrum of compound **3c** (150 MHz, $CDCl_3$, 298 K)

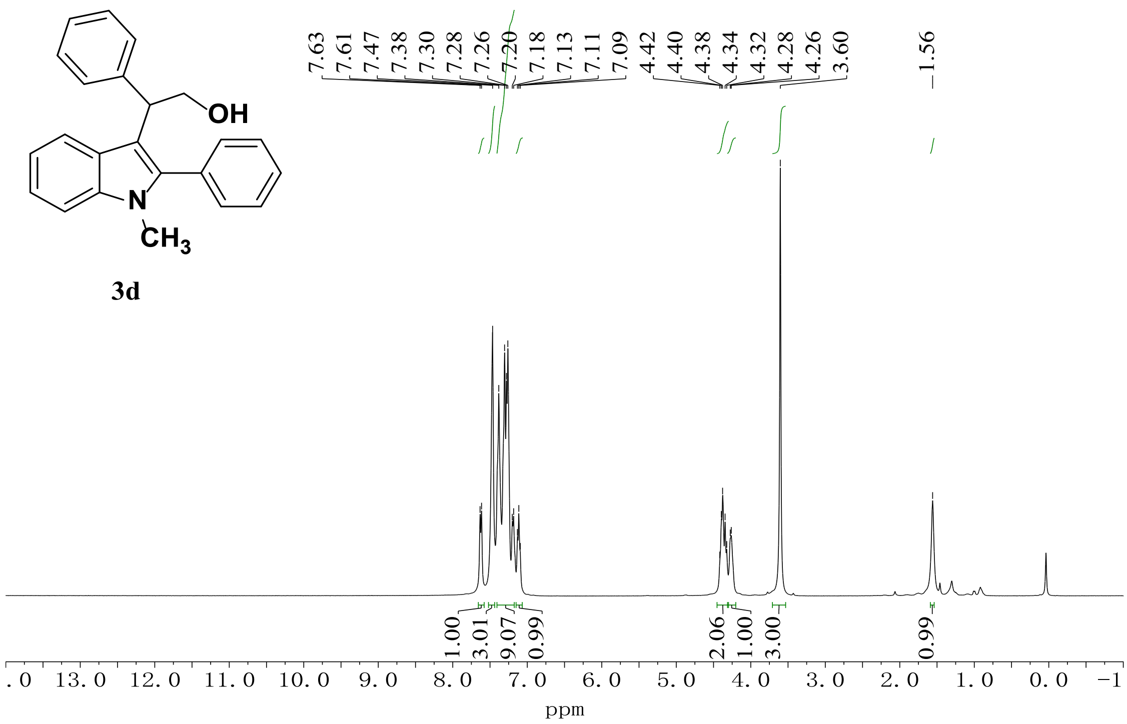

^{1}H NMR spectrum of compound **3d** (400 MHz, $CDCl_3$, 298 K)

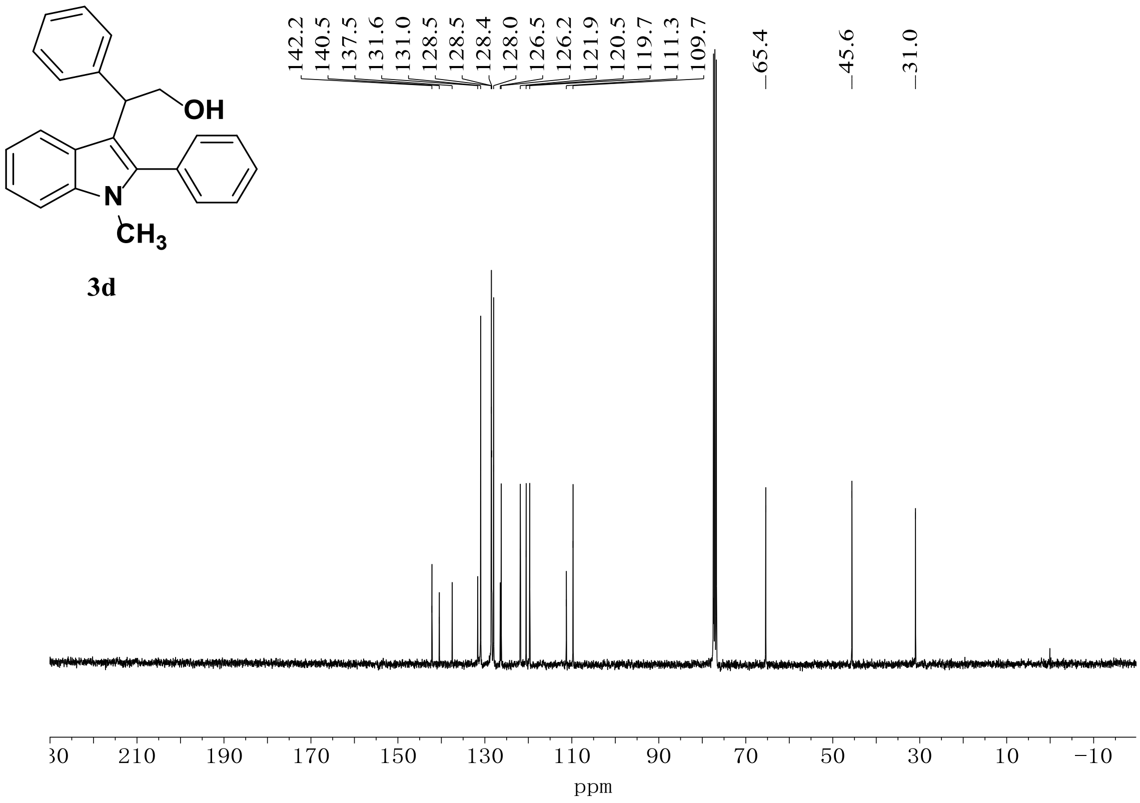

^{13}C NMR spectrum of compound **3d** (100 MHz, $CDCl_3$, 298 K)

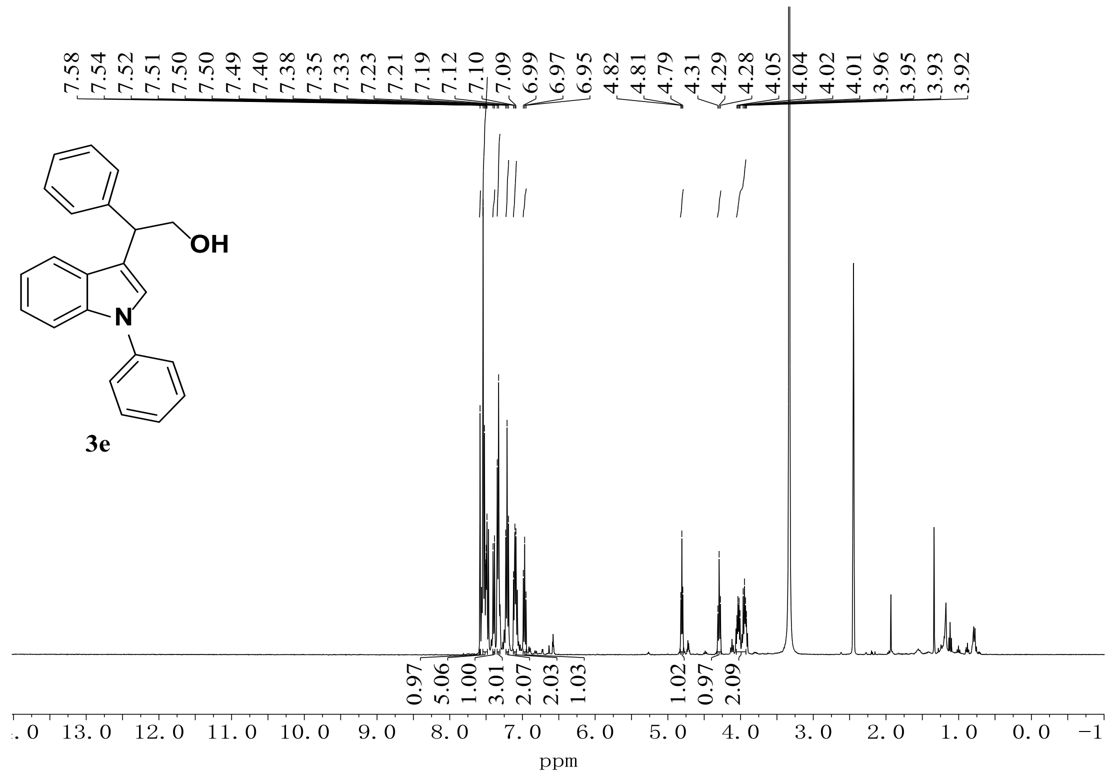

^{1}H NMR spectrum of compound **3e** (400 MHz, DMSO-*d*6, 298 K)

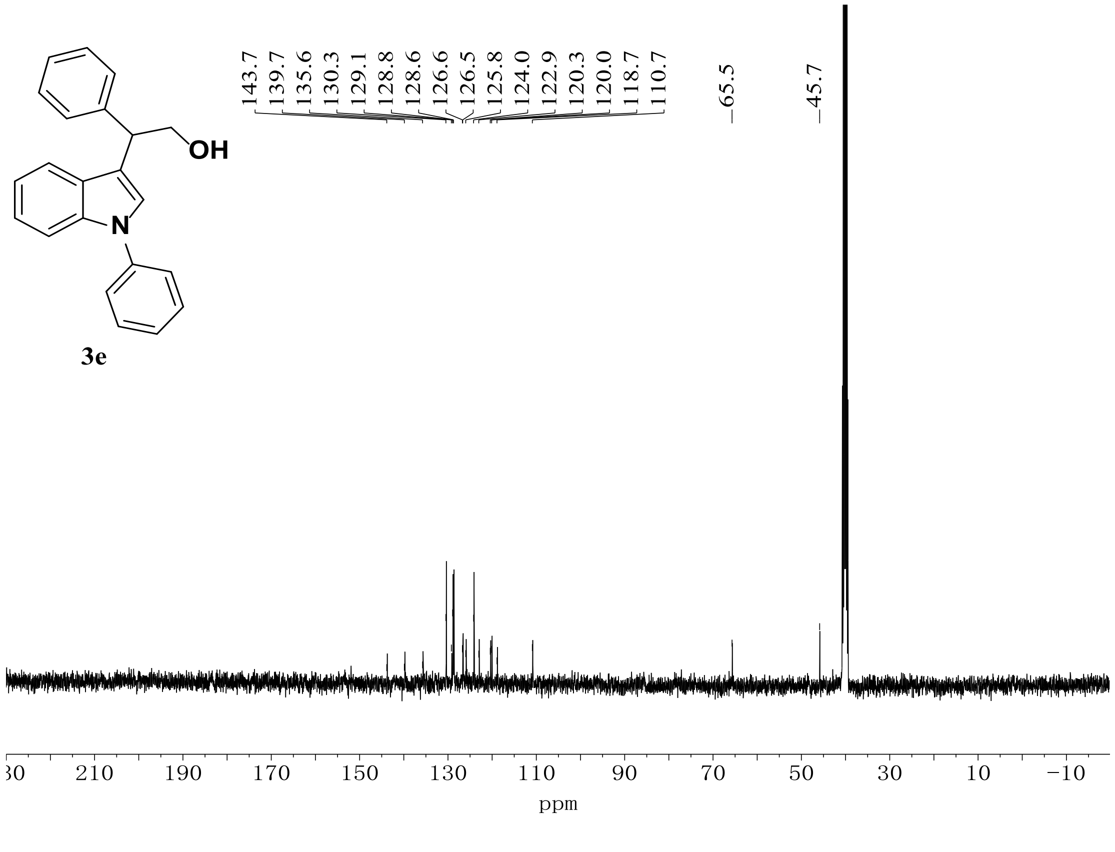

^{13}C NMR spectrum of compound **3e** (100 MHz, DMSO-*d*6, 298 K)

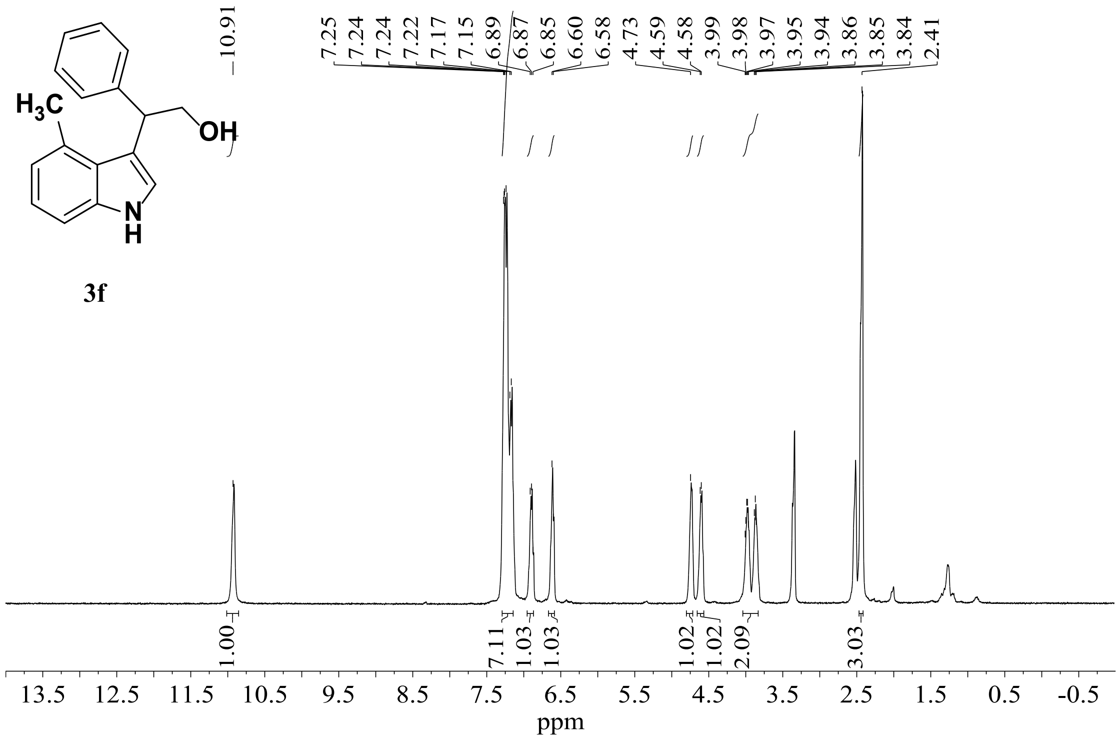

^{1}H NMR spectrum of compound **3f** (400 MHz, DMSO-*d*6, 298 K)

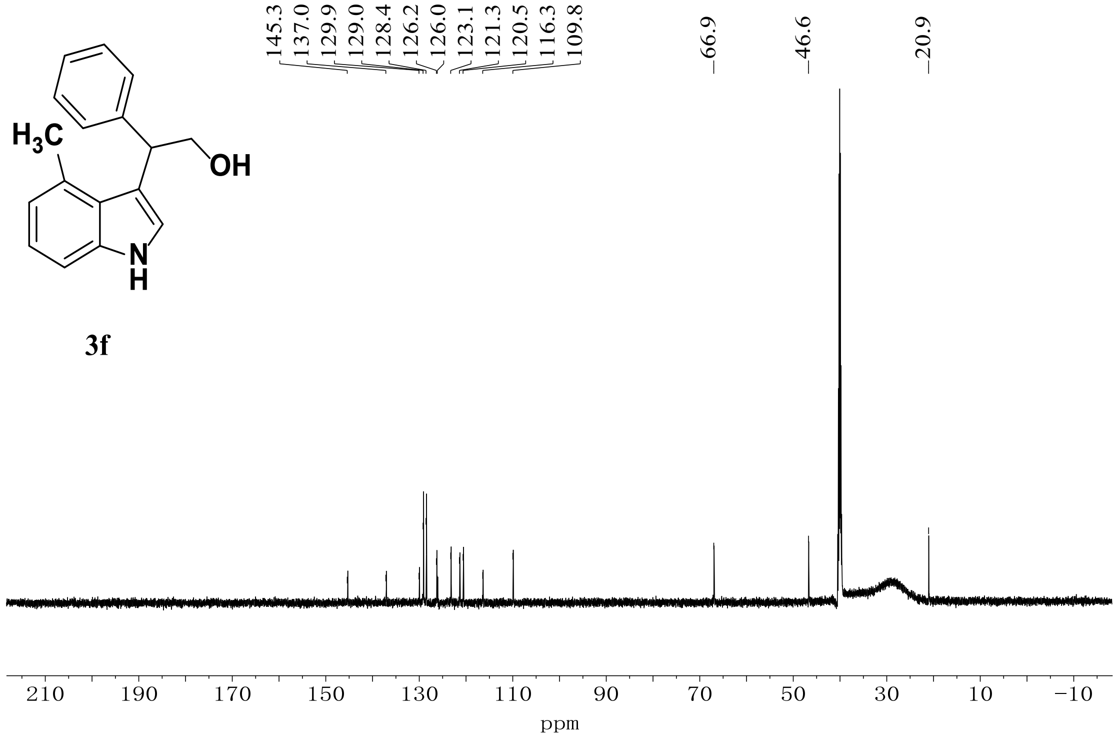

^{13}C NMR spectrum of compound **3f** (100 MHz, DMSO-*d*6, 298 K)

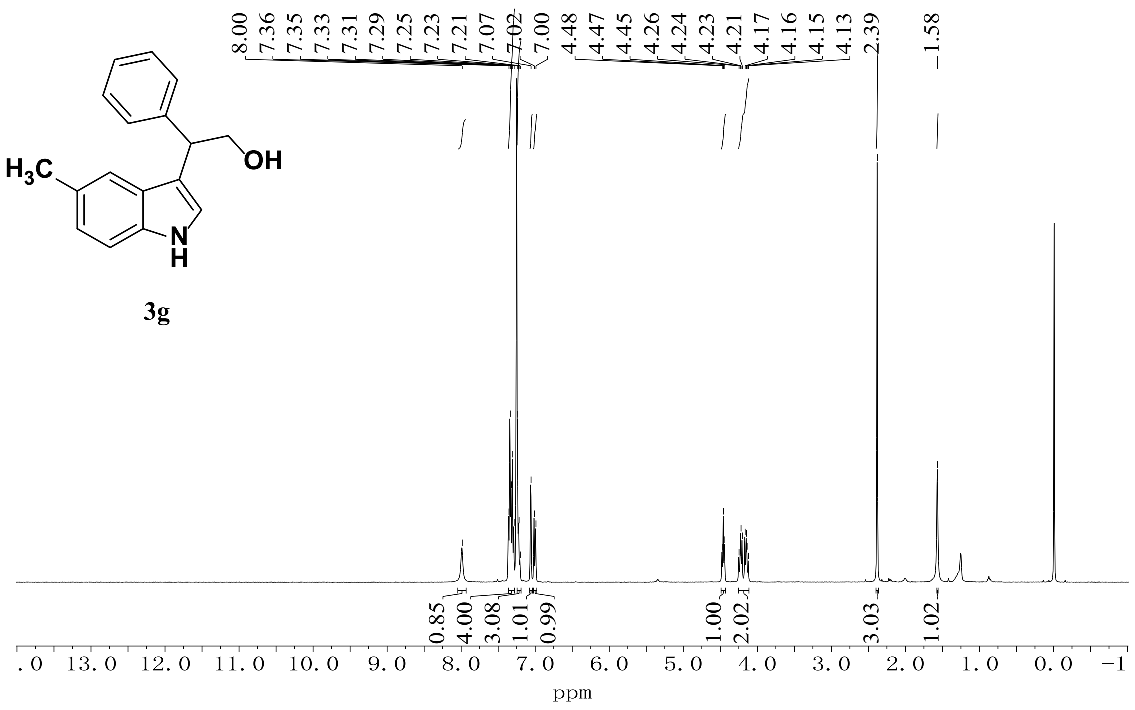

^{1}H NMR spectrum of compound **3g** (400 MHz, $CDCl_3$, 298 K)

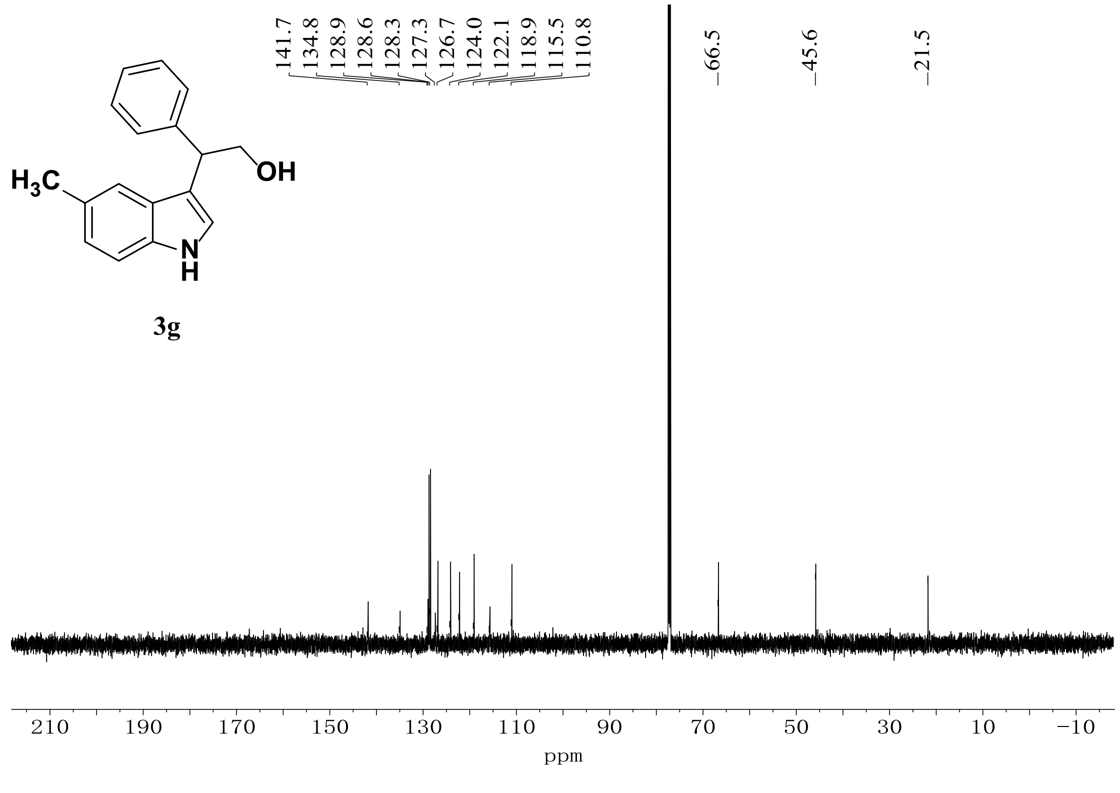

^{13}C NMR spectrum of compound **3g** (100 MHz, $CDCl_3$, 298 K)

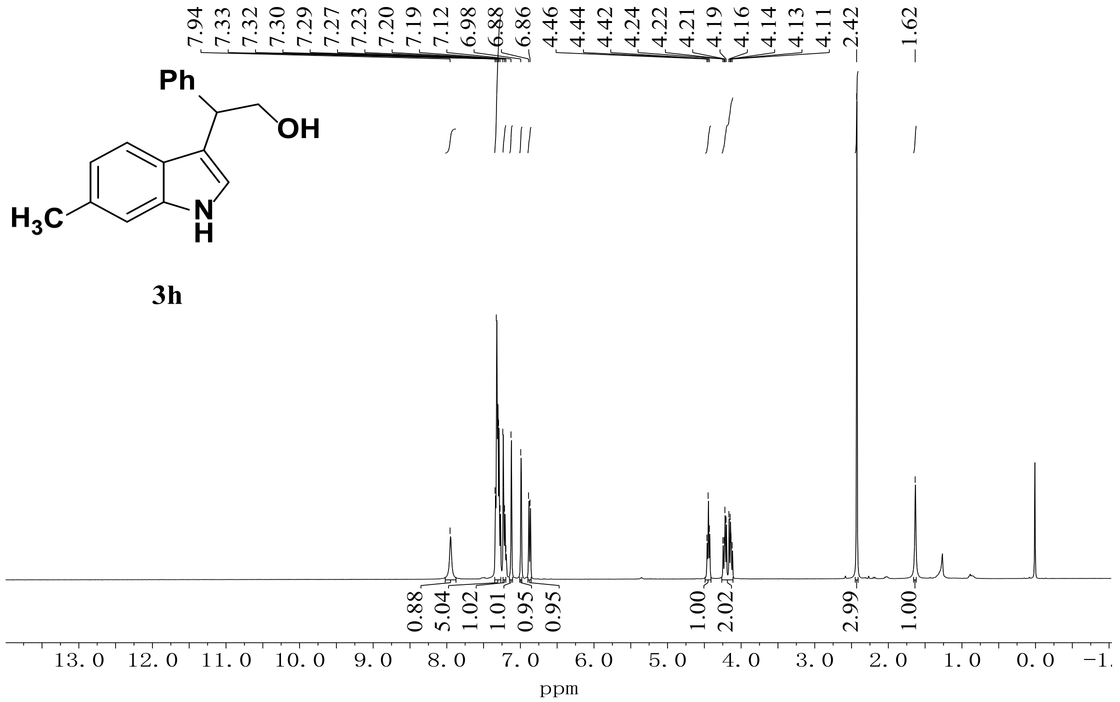

^{1}H NMR spectrum of compound **3h** (400 MHz, $CDCl_3$, 298 K)

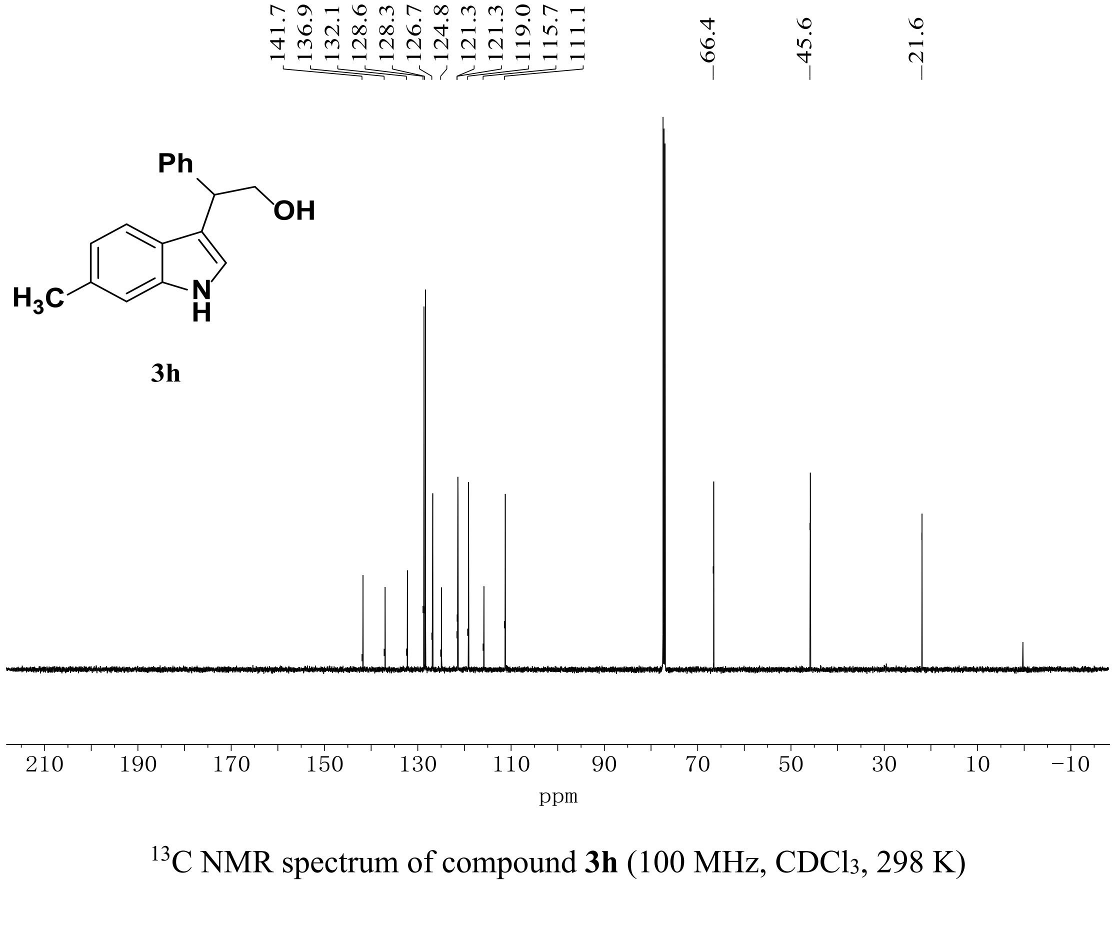

^{13}C NMR spectrum of compound **3h** (100 MHz, $CDCl_3$, 298 K)

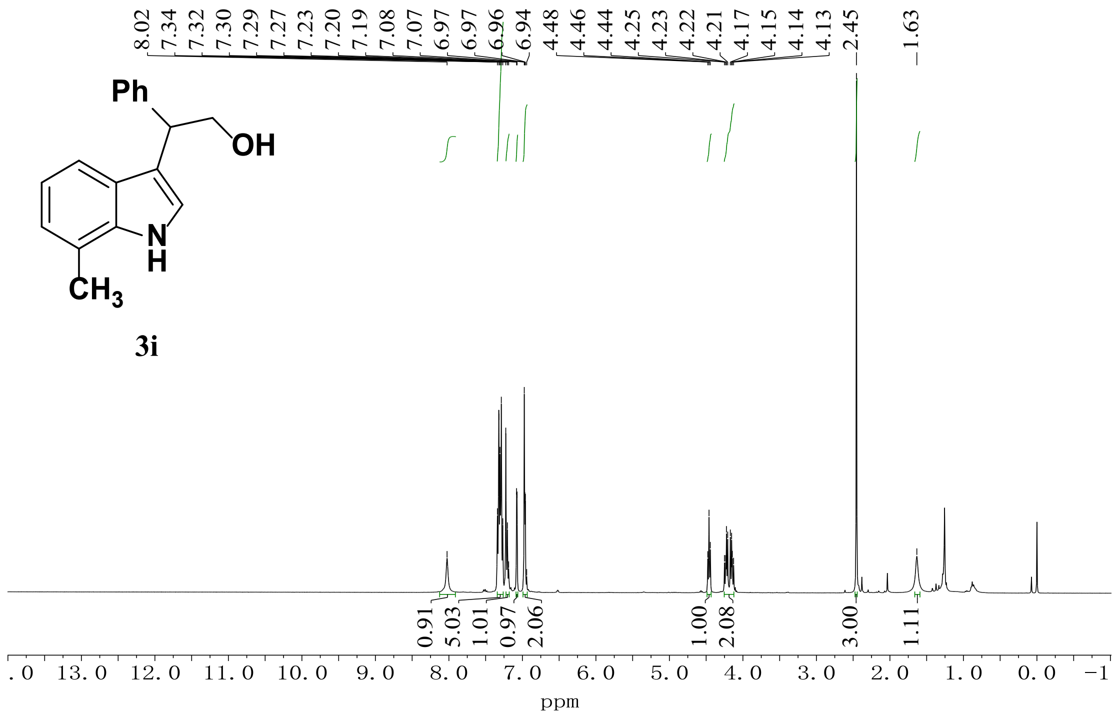

^{1}H NMR spectrum of compound **3i** (400 MHz, $CDCl_3$, 298 K)

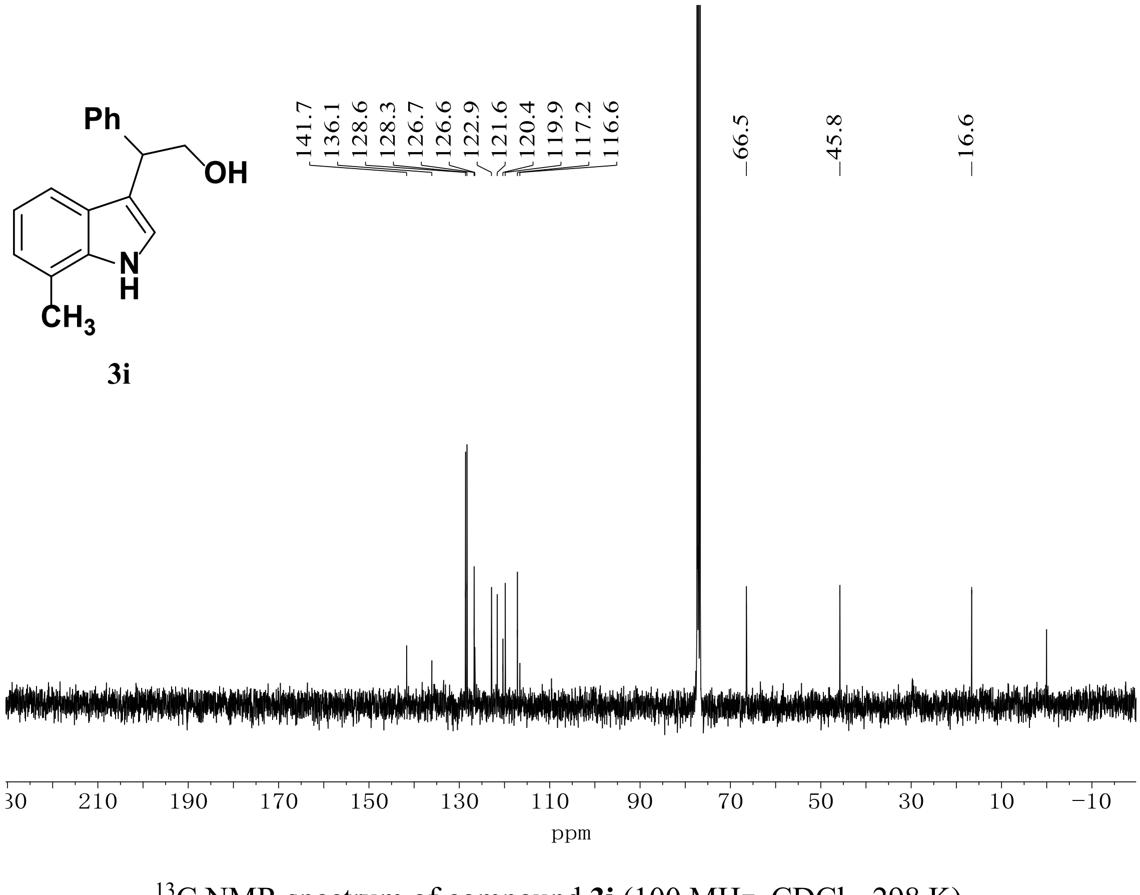

^{13}C NMR spectrum of compound **3i** (100 MHz, $CDCl_3$, 298 K)

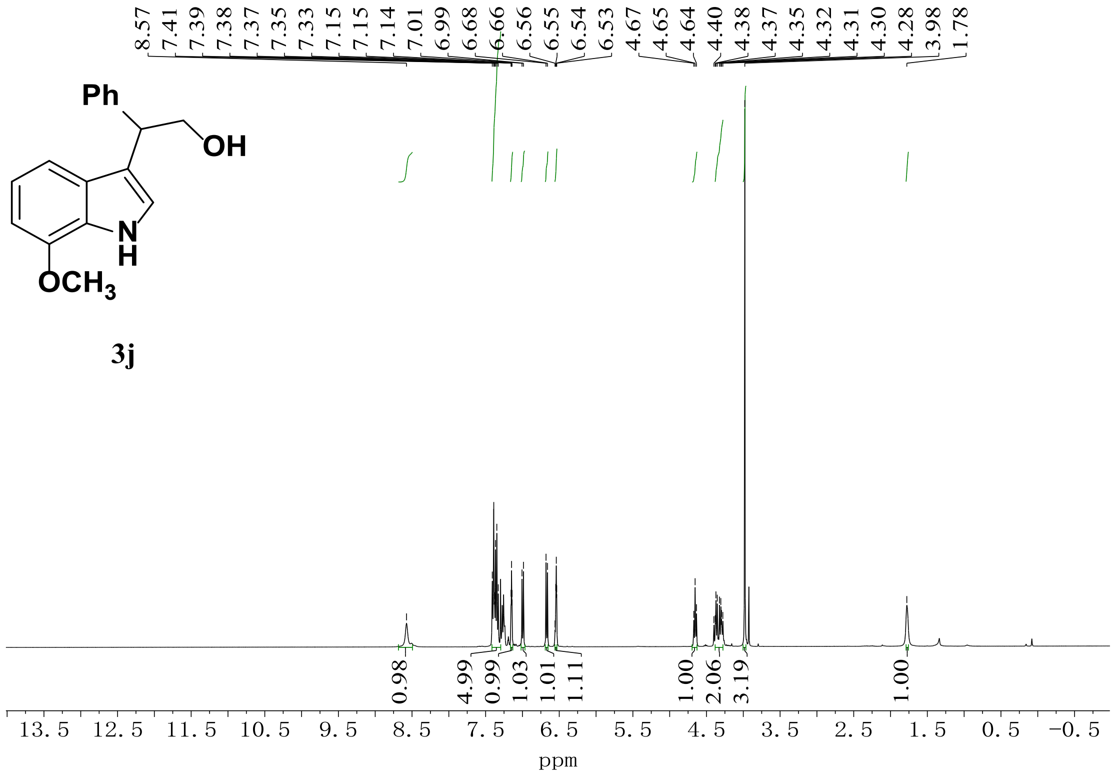

^{1}H NMR spectrum of compound **3j** (400 MHz, $CDCl_3$, 298 K)

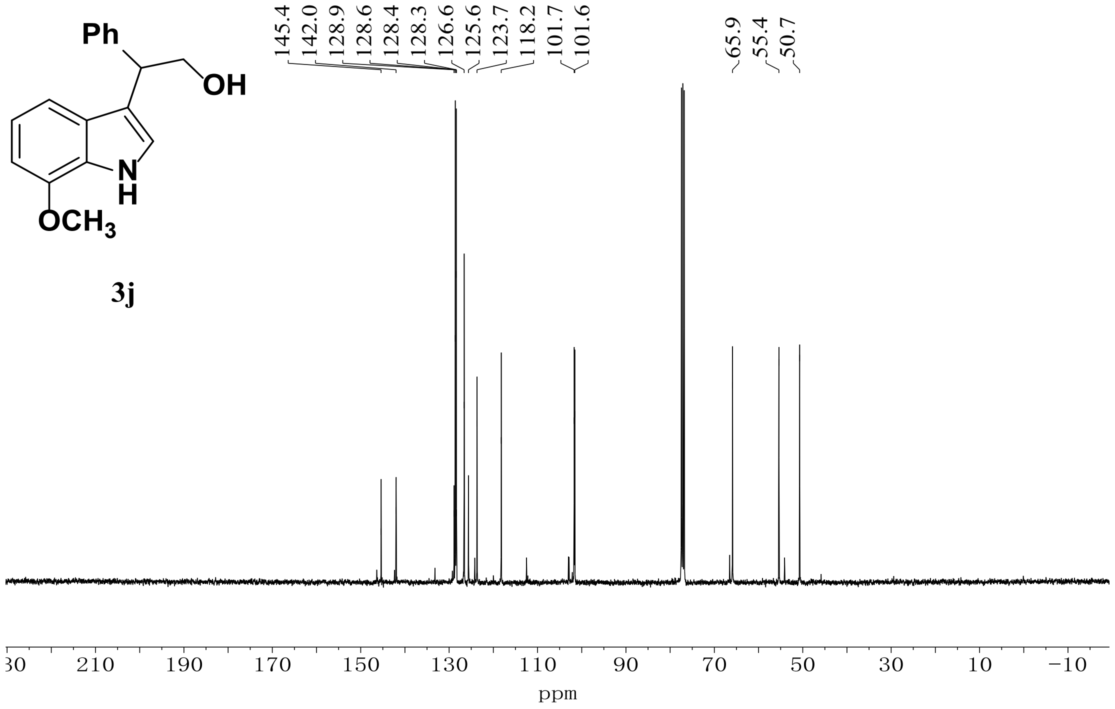

^{13}C NMR spectrum of compound **3j** (100 MHz, $CDCl_3$, 298 K)

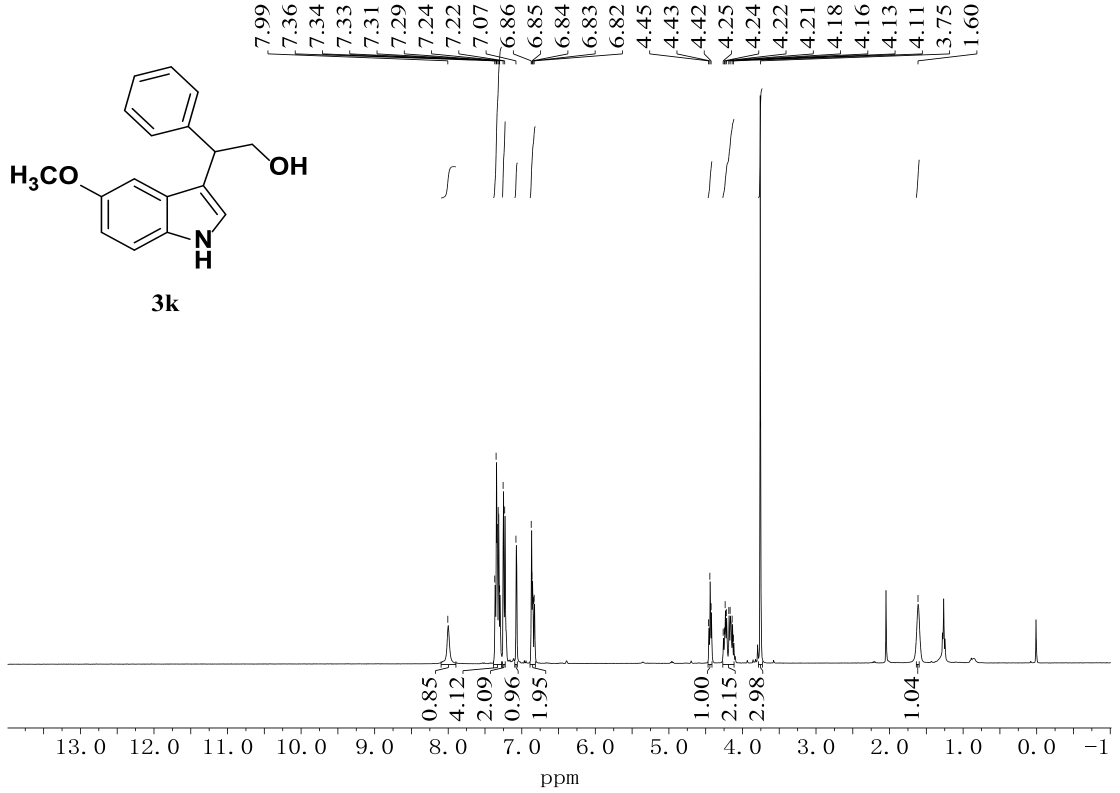

^{1}H NMR spectrum of compound **3k** (400 MHz, $CDCl_3$, 298 K)

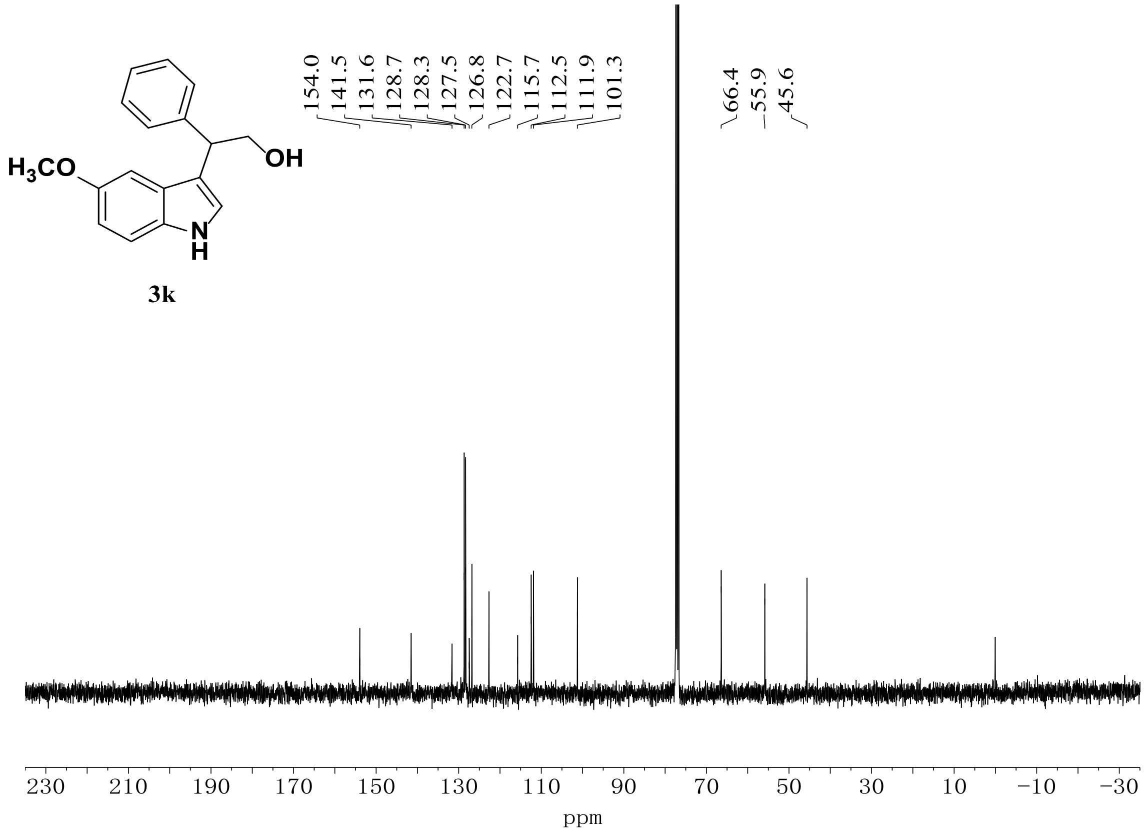

^{13}C NMR spectrum of compound **3k** (100 MHz, $CDCl_3$, 298 K)

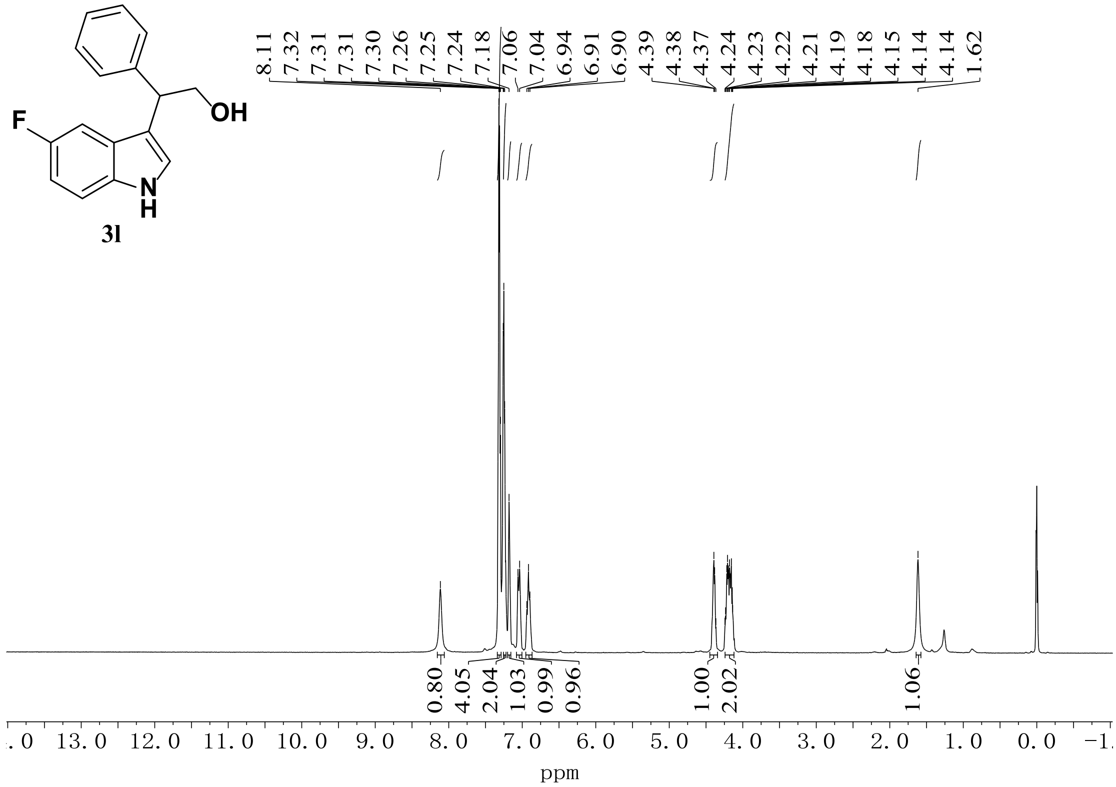

^{1}H NMR spectrum of compound **3l** (400 MHz, $CDCl_3$, 298 K)

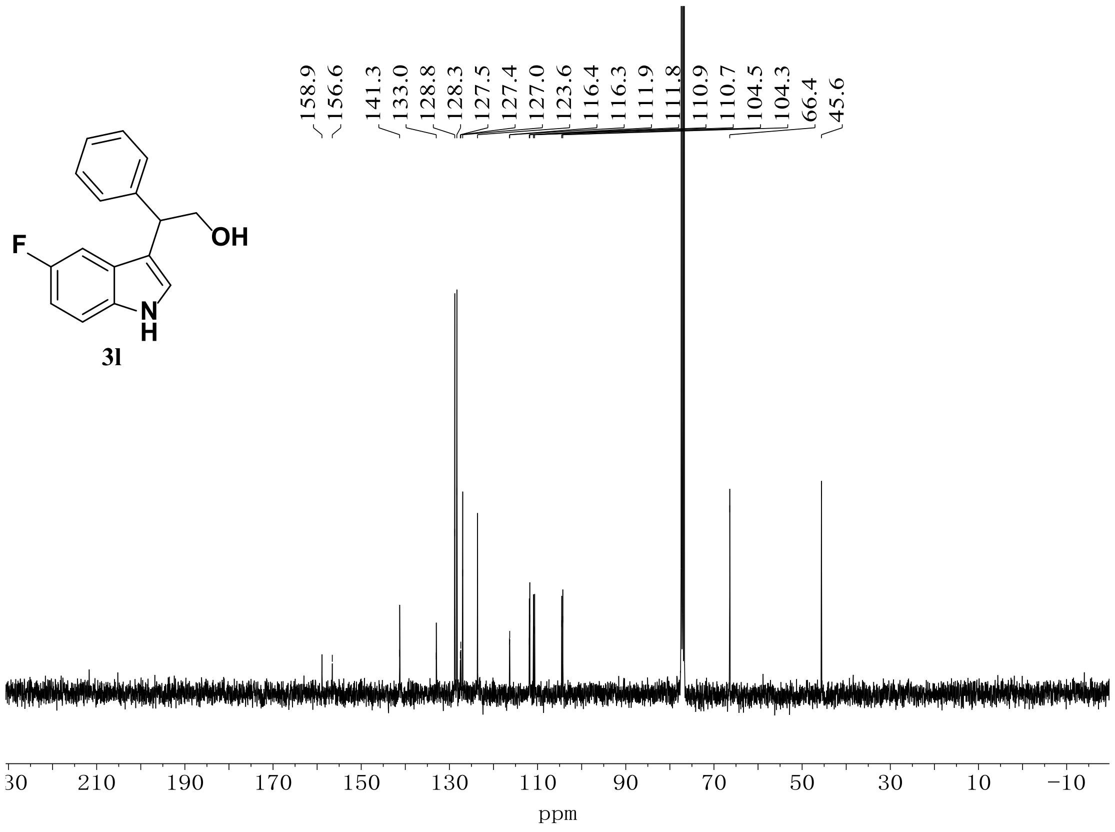

^{13}C NMR spectrum of compound **3l** (100 MHz, $CDCl_3$, 298 K)

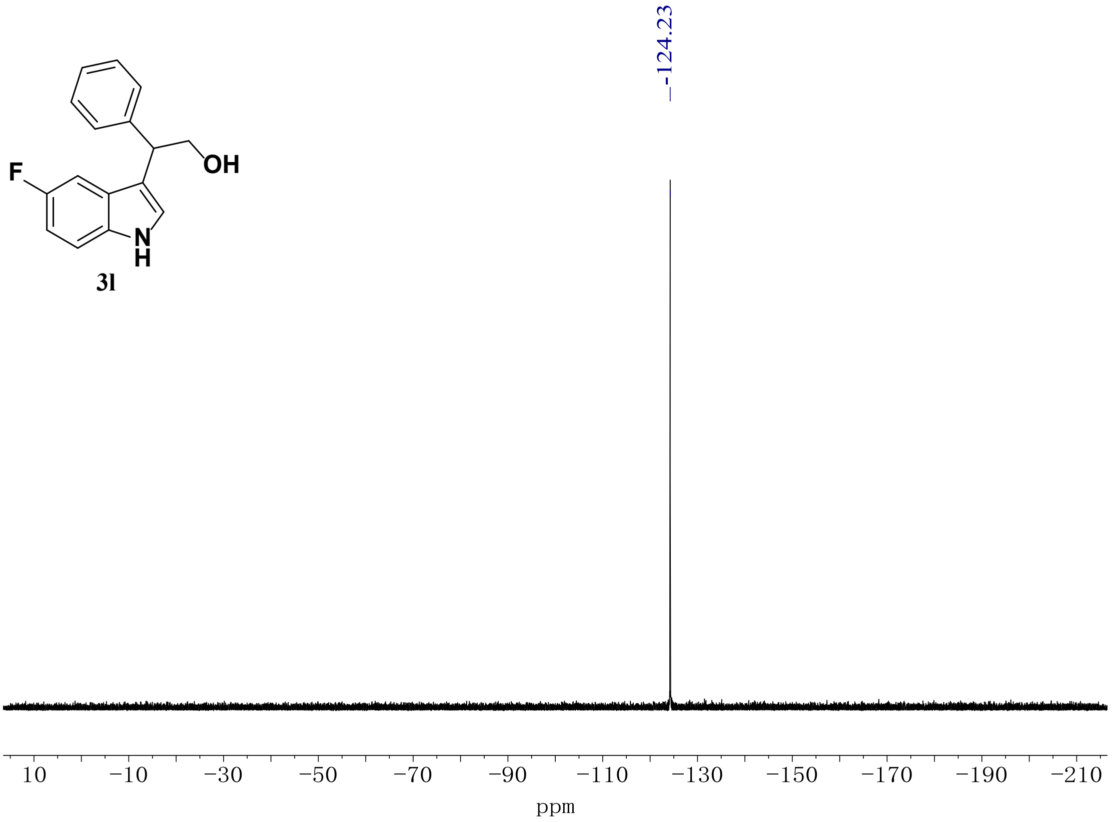

^{19}F NMR spectrum of compound **3l** (376 MHz, $CDCl_3$, 298 K)

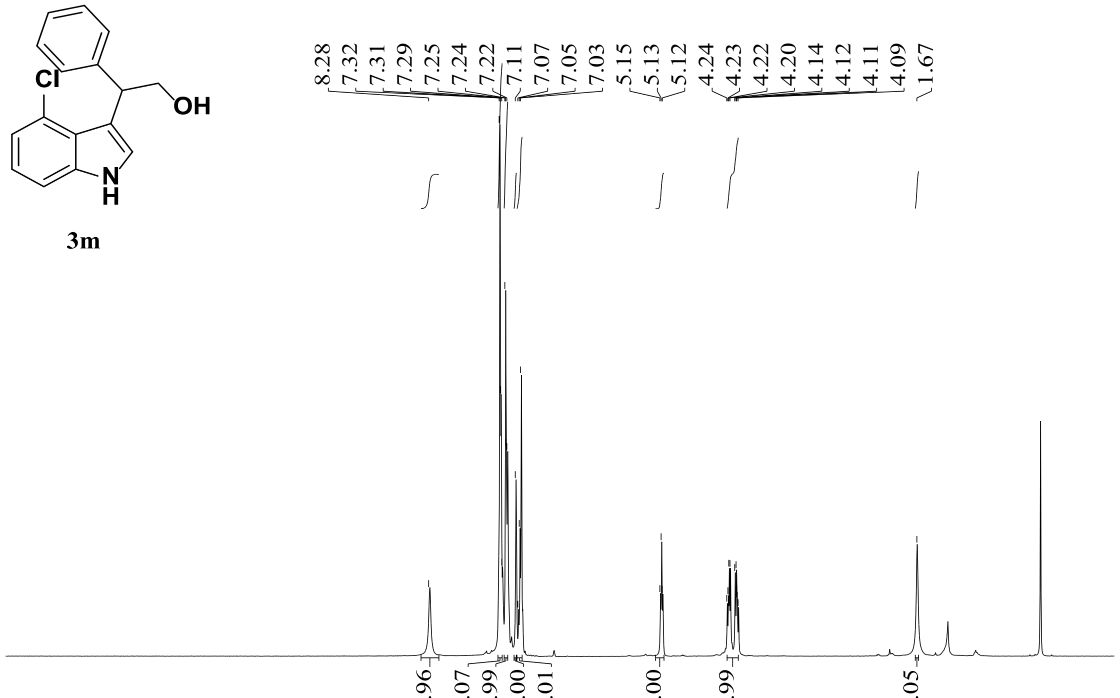

^{1}H NMR spectrum of compound **3m** (400 MHz, $CDCl_3$, 298 K)

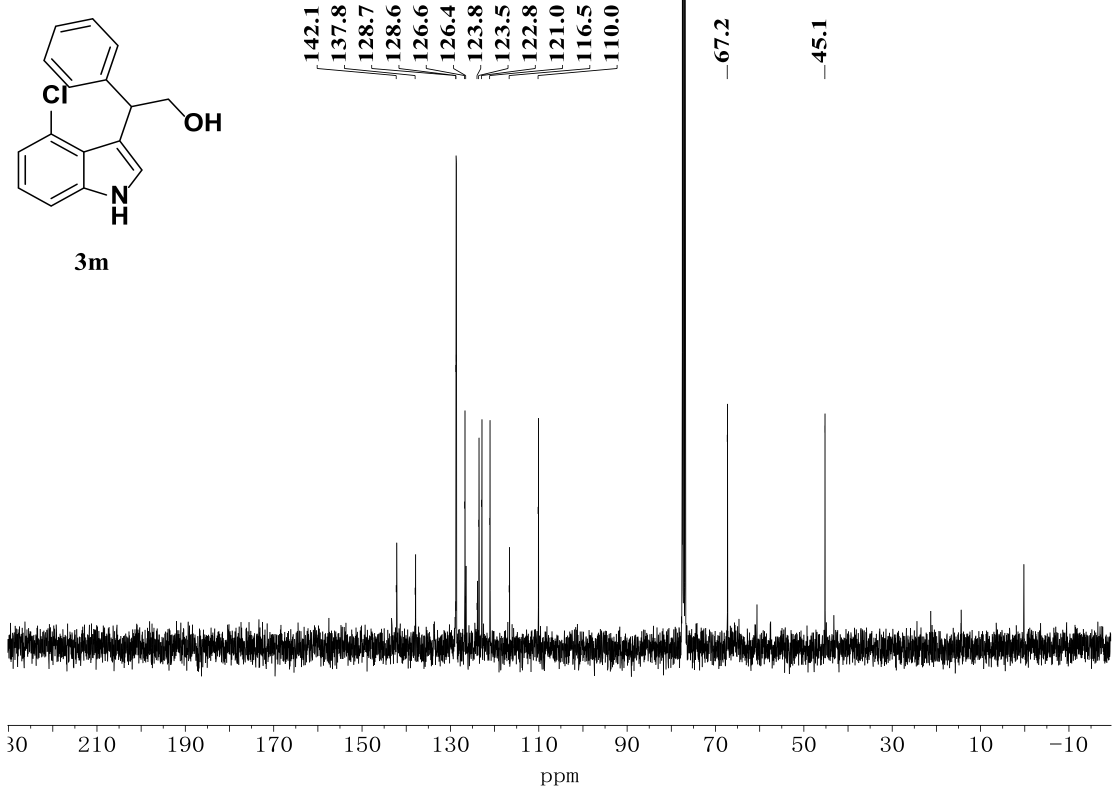

^{13}C NMR spectrum of compound **3m** (100 MHz, $CDCl_3$, 298 K)

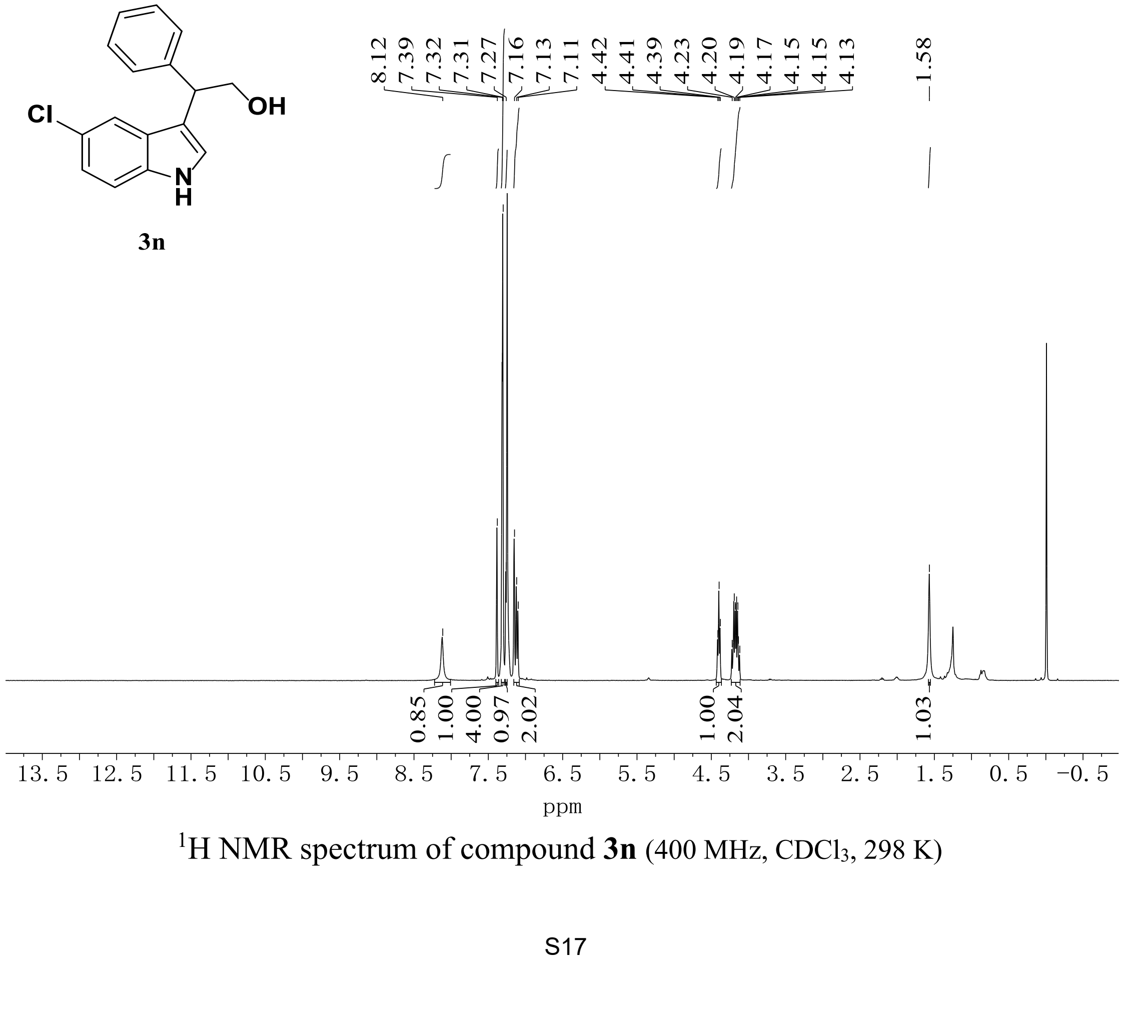

^{1}H NMR spectrum of compound **3n** (400 MHz, $CDCl_3$, 298 K)

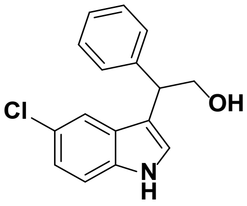

3n

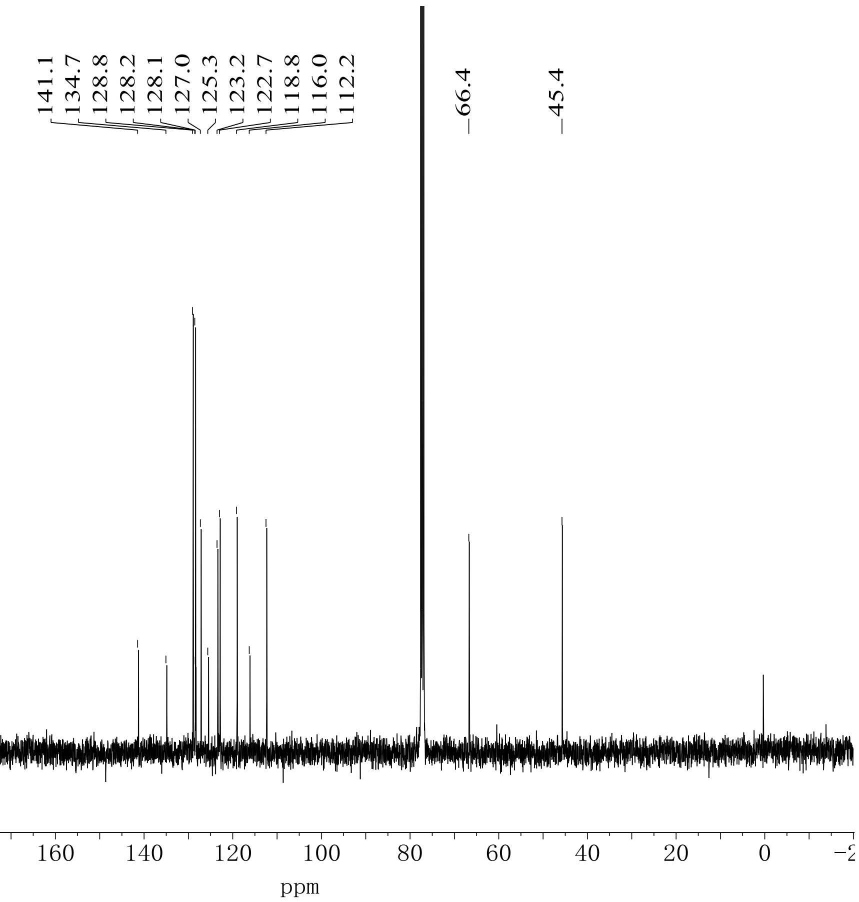

^{13}C NMR spectrum of compound **3n** (100 MHz, $CDCl_3$, 298 K)

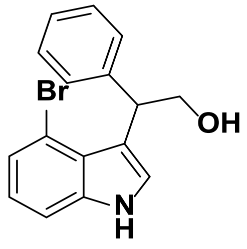

3o

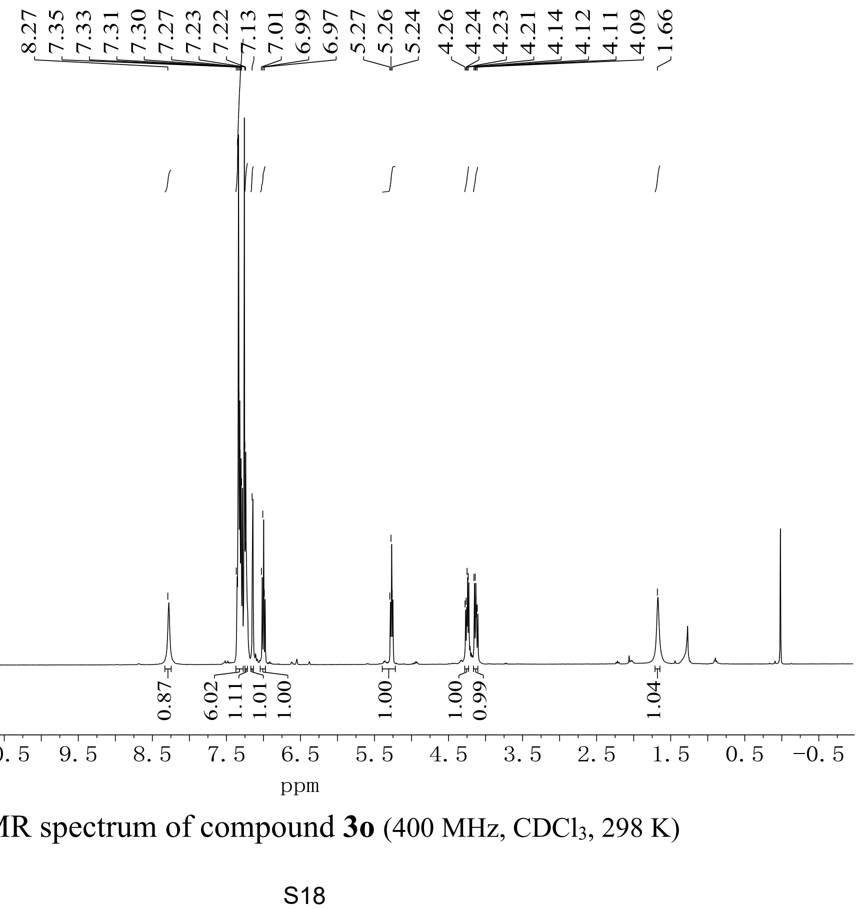

^{1}H NMR spectrum of compound **3o** (400 MHz, $CDCl_3$, 298 K)

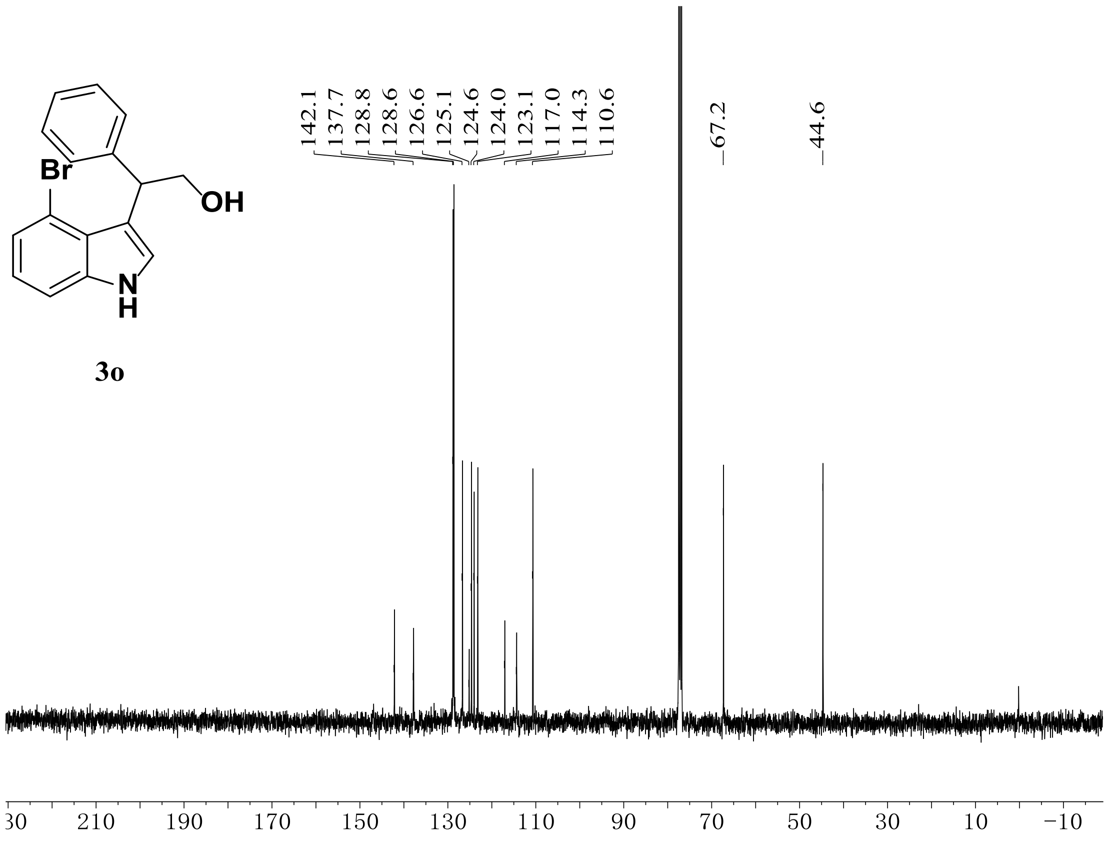

^{13}C NMR spectrum of compound **3o** (100 MHz, $CDCl_3$, 298 K)

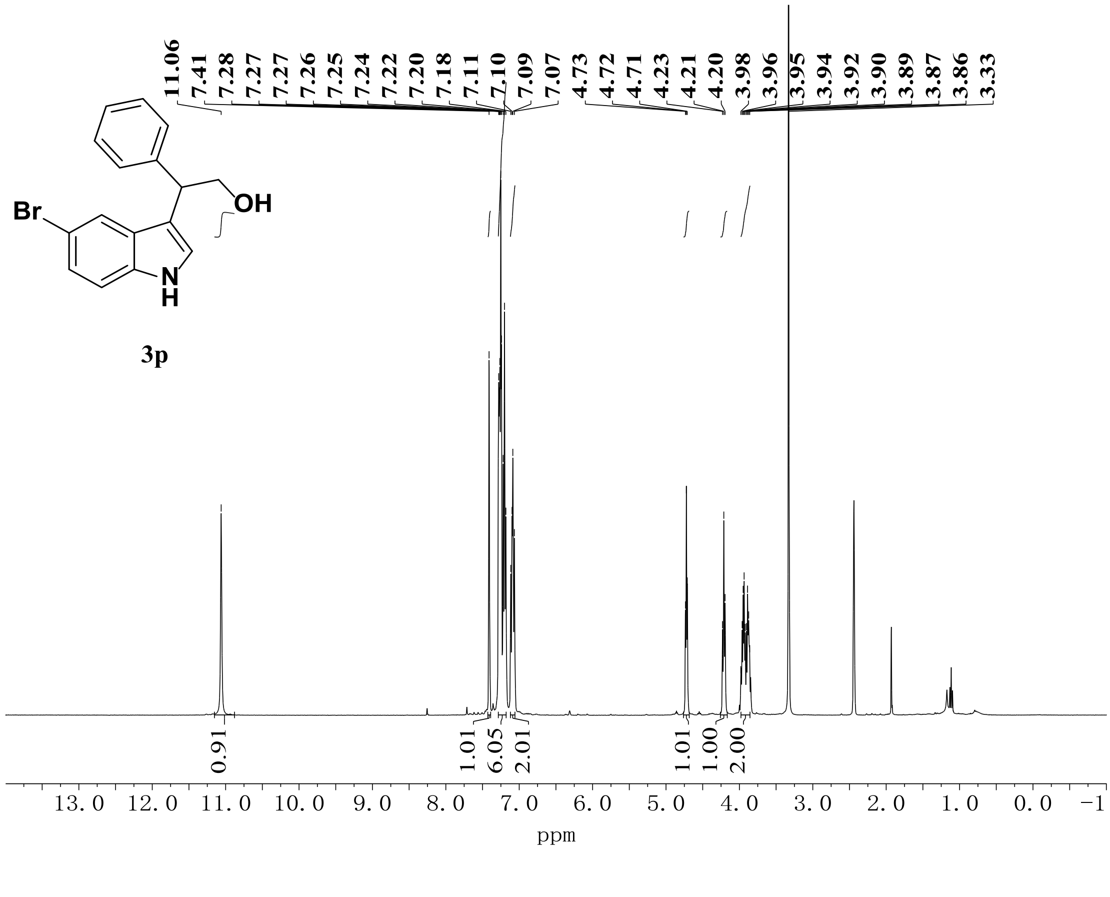

^{1}H NMR spectrum of compound **3p** (400 MHz, DMSO-*d*6, 298 K)

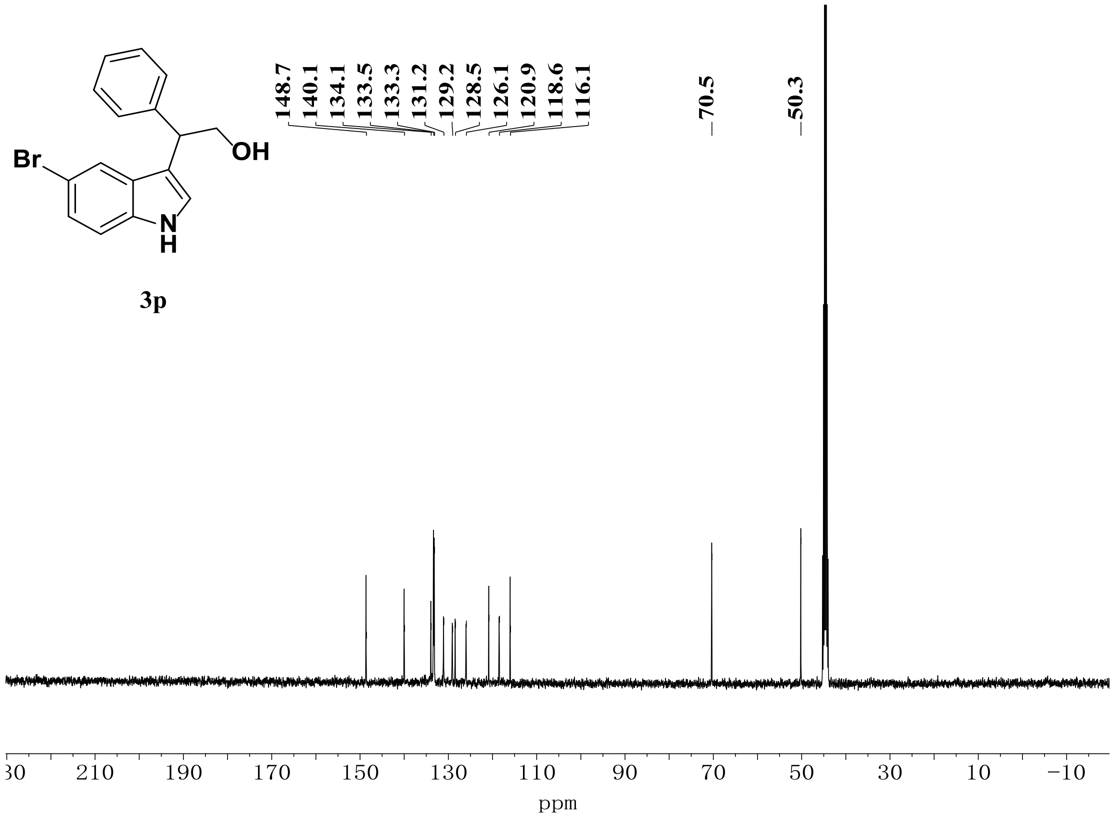

^{13}C NMR spectrum of compound **3p** (100 MHz, DMSO-*d*6, 298 K)

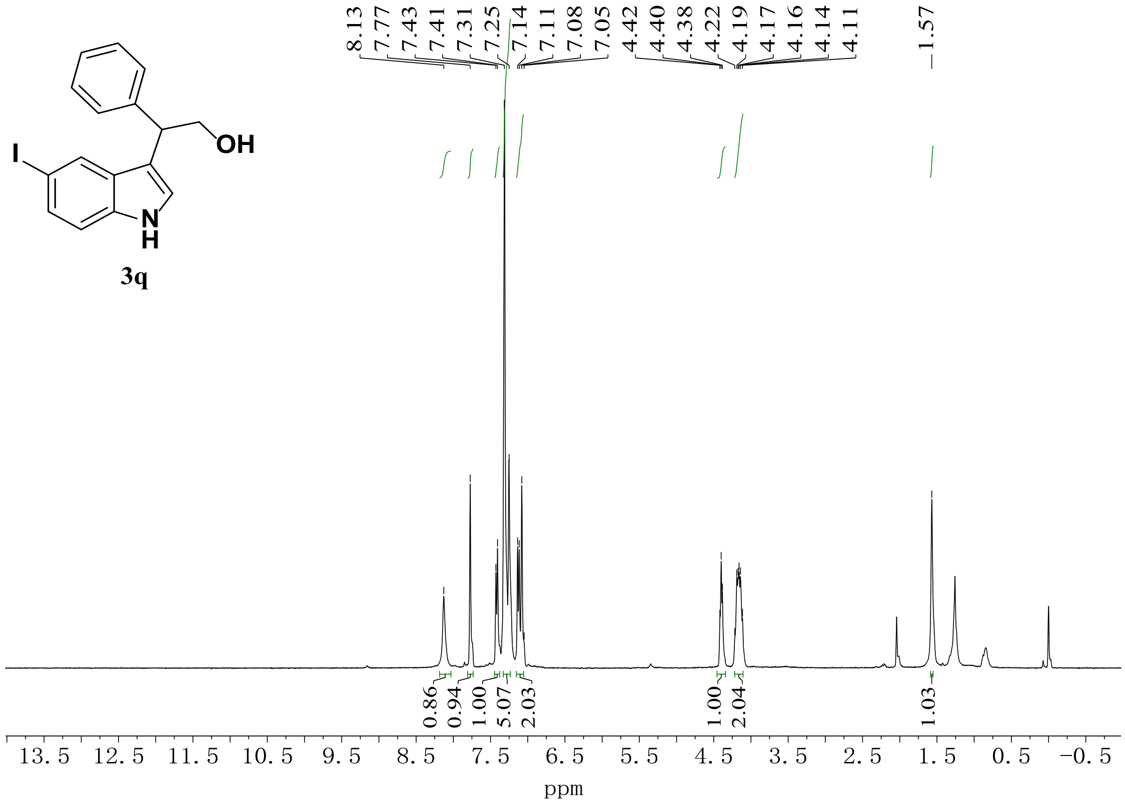

^{1}H NMR spectrum of compound **3q** (400 MHz, $CDCl_3$, 298 K)

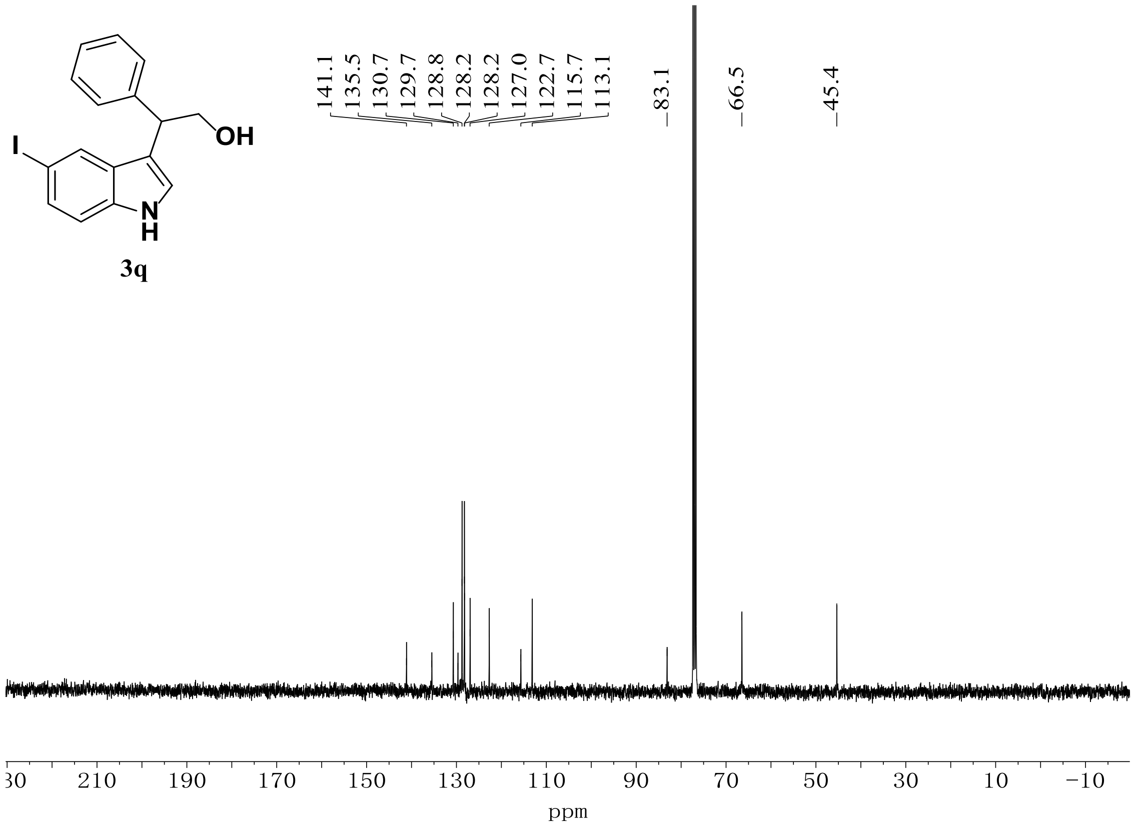

^{13}C NMR spectrum of compound **3q** (100 MHz, $CDCl_3$, 298 K)

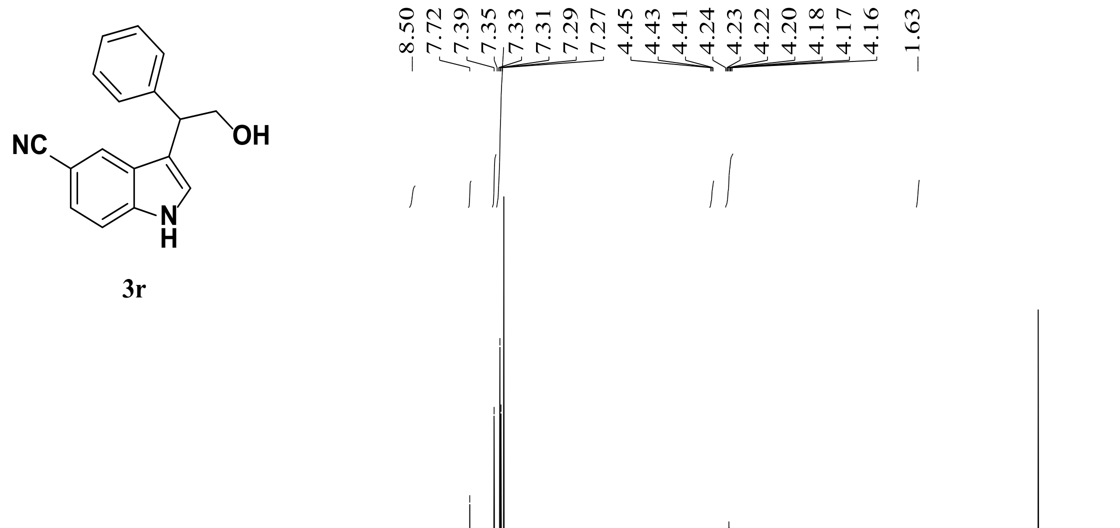

^{1}H NMR spectrum of compound **3r** (400 MHz, $CDCl_3$, 298 K)

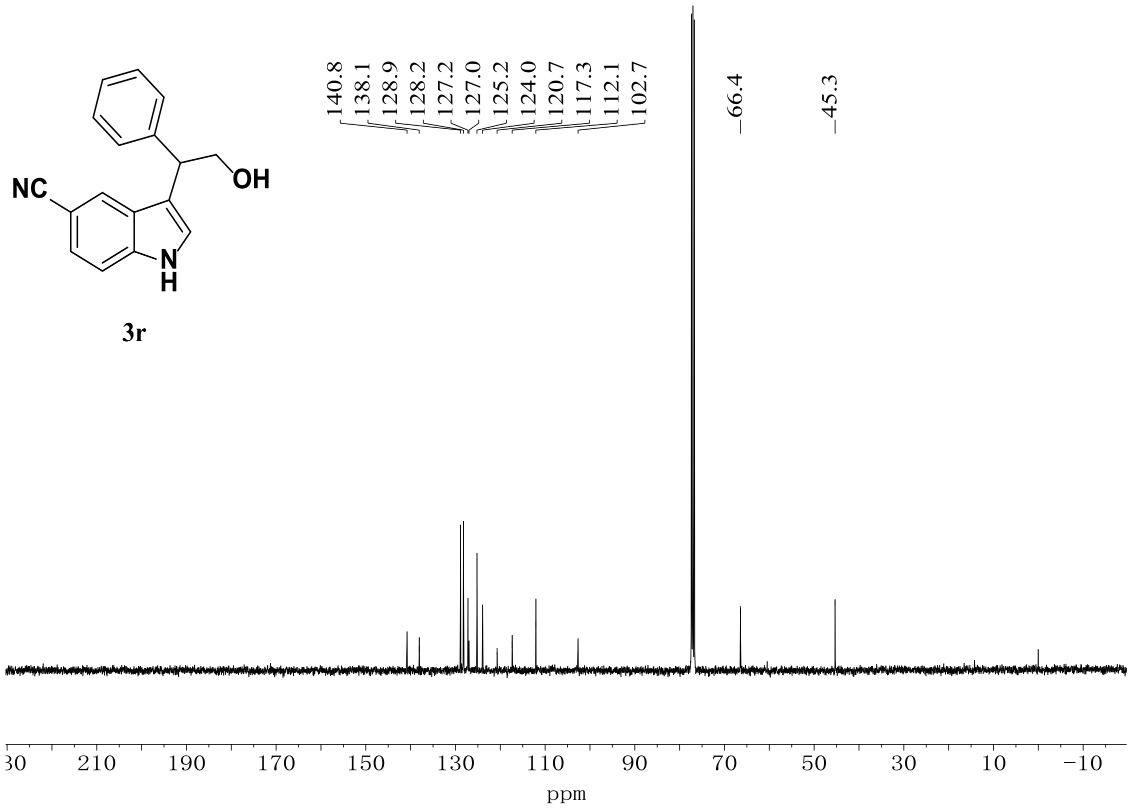

3r

^{13}C NMR spectrum of compound **3r** (100 MHz, $CDCl_3$, 298 K)

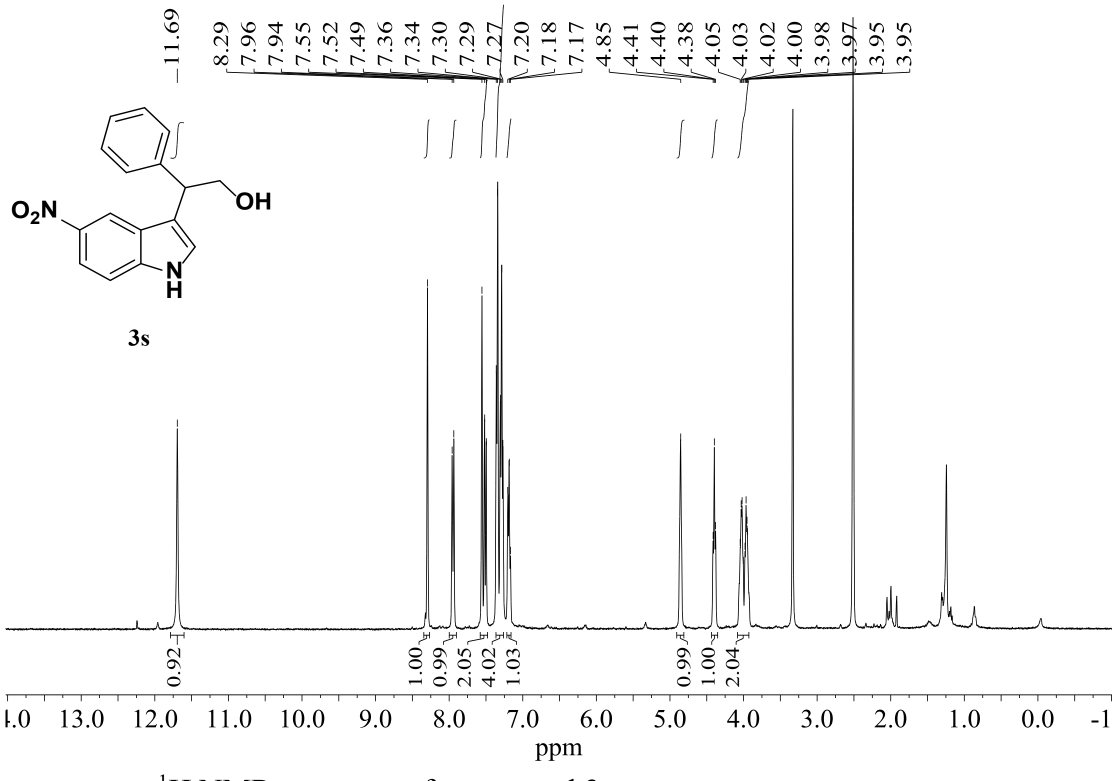

3s

^{1}H NMR spectrum of compound **3s** (400 MHz, DMSO-*d*6, 298 K)

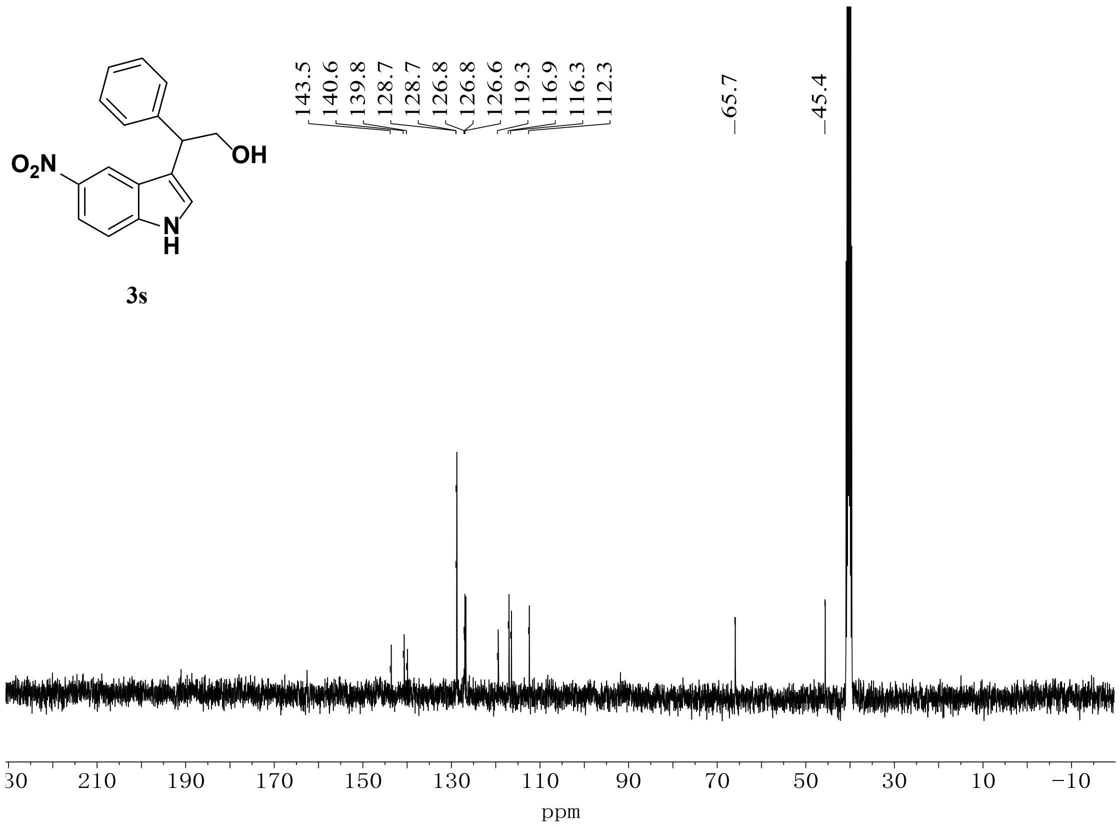

3s

^{13}C NMR spectrum of compound **3s** (400 MHz, DMSO-*d6*, 298 K)

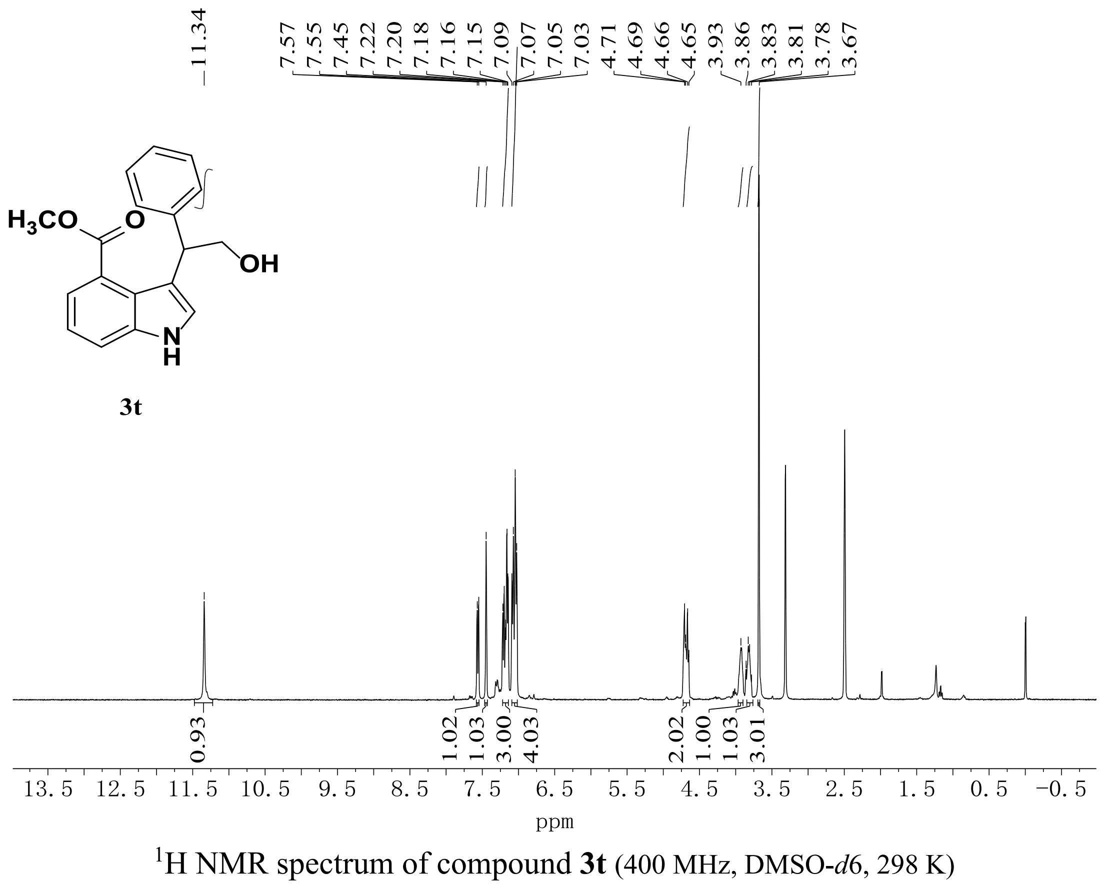

3t

^{1}H NMR spectrum of compound **3t** (400 MHz, DMSO-*d6*, 298 K)

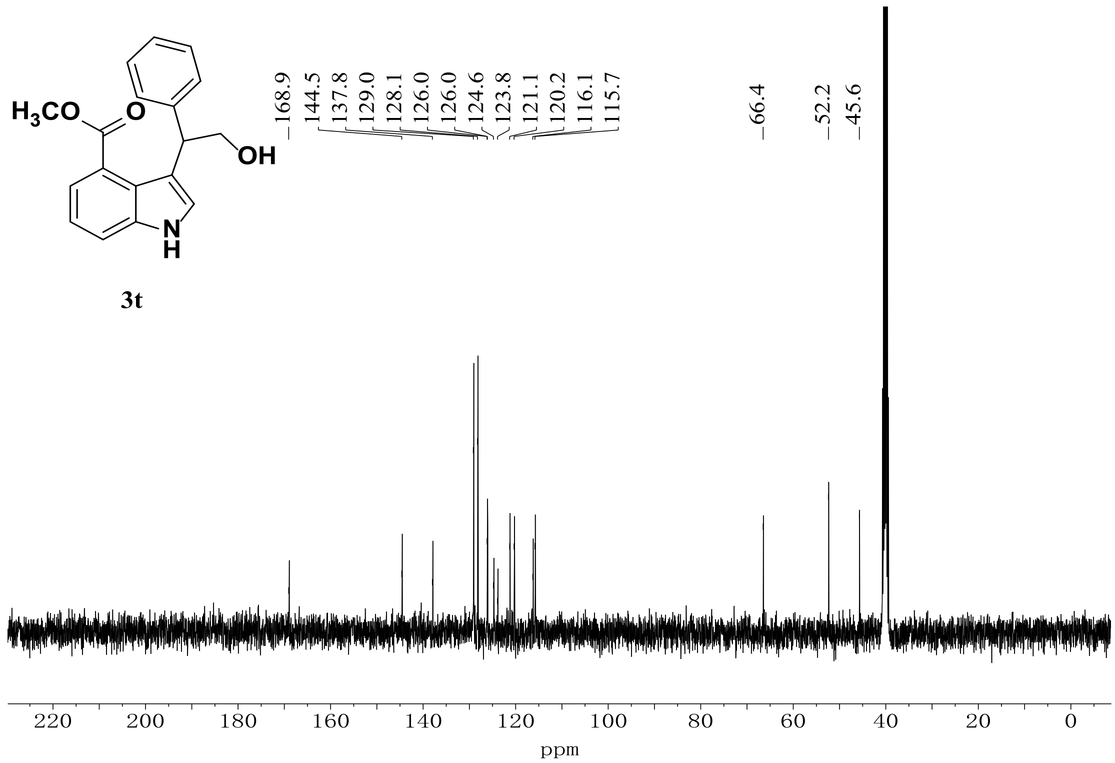

^{13}C NMR spectrum of compound **3t** (100 MHz, DMSO-*d*6, 298 K)

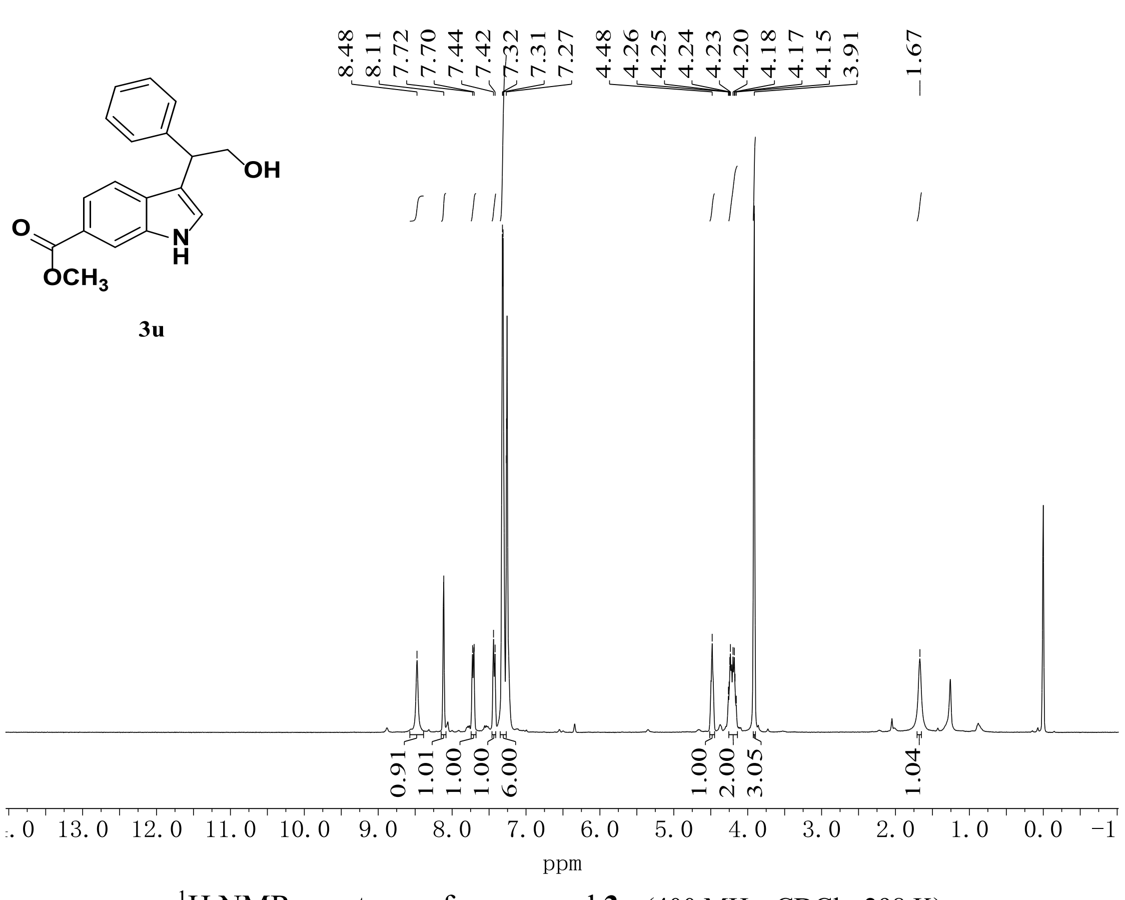

^{1}H NMR spectrum of compound **3u** (400 MHz, $CDCl_3$, 298 K)

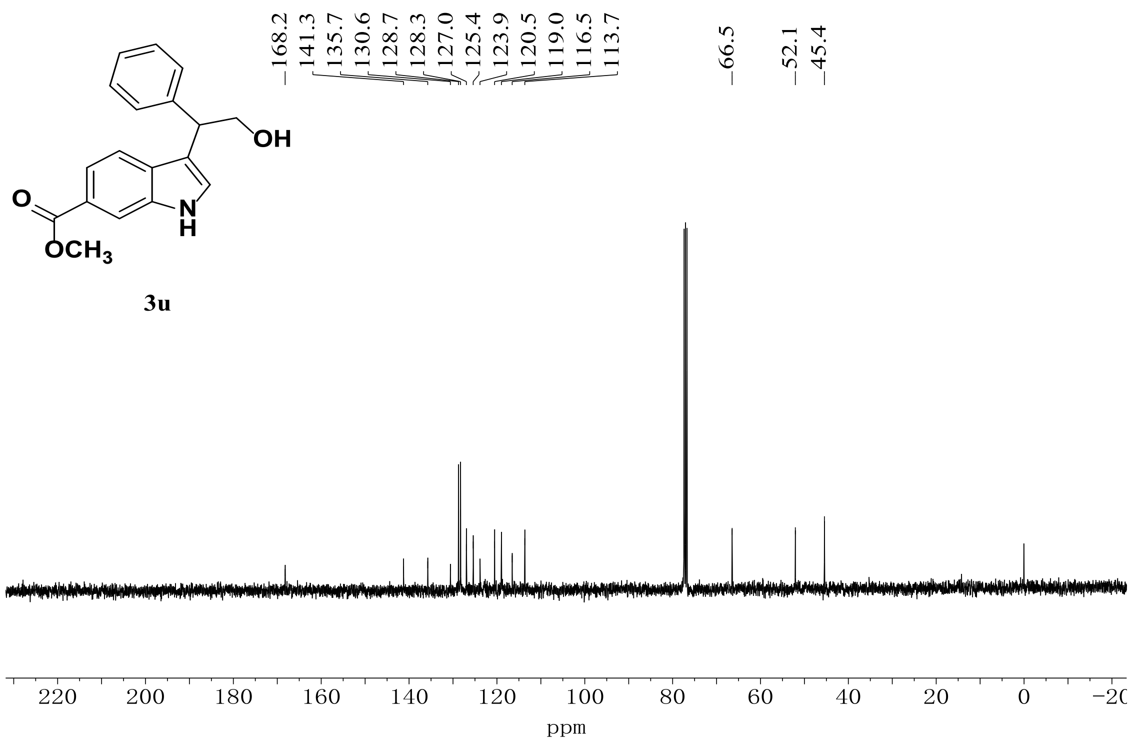

^{13}C NMR spectrum of compound **3u** (100 MHz, $CDCl_3$, 298 K)

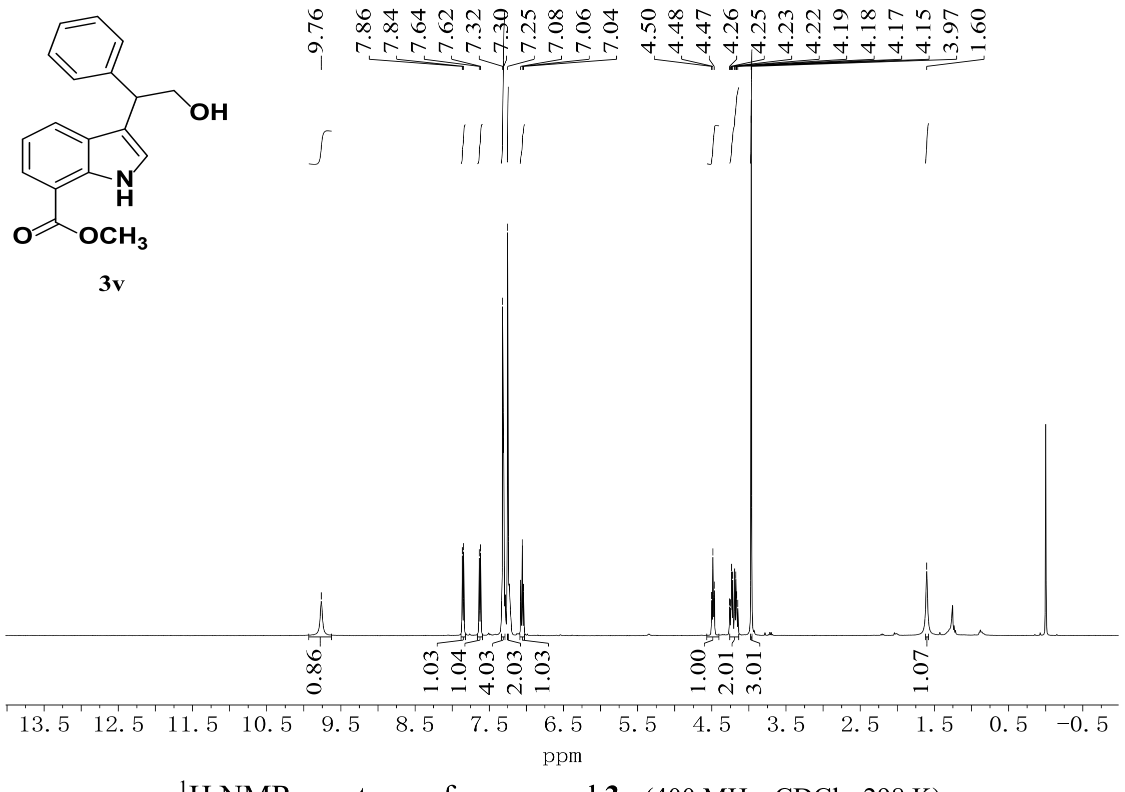

^{1}H NMR spectrum of compound **3v** (400 MHz, $CDCl_3$, 298 K)

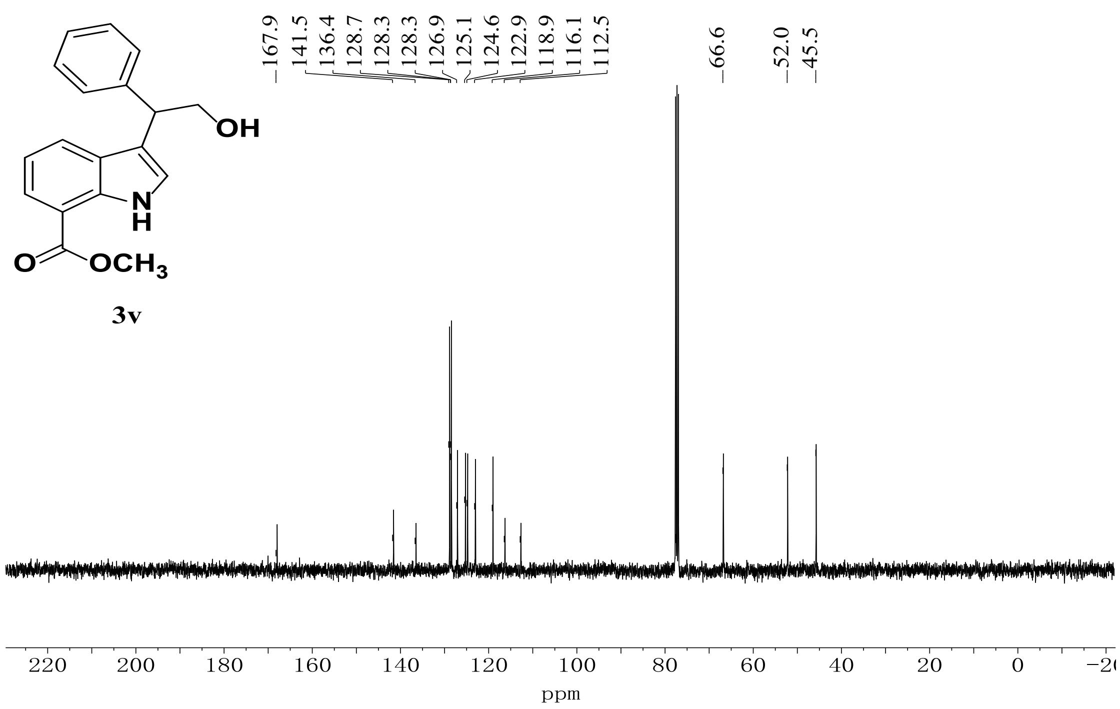

^{13}C NMR spectrum of compound **3v** (100 MHz, $CDCl_3$, 298 K)

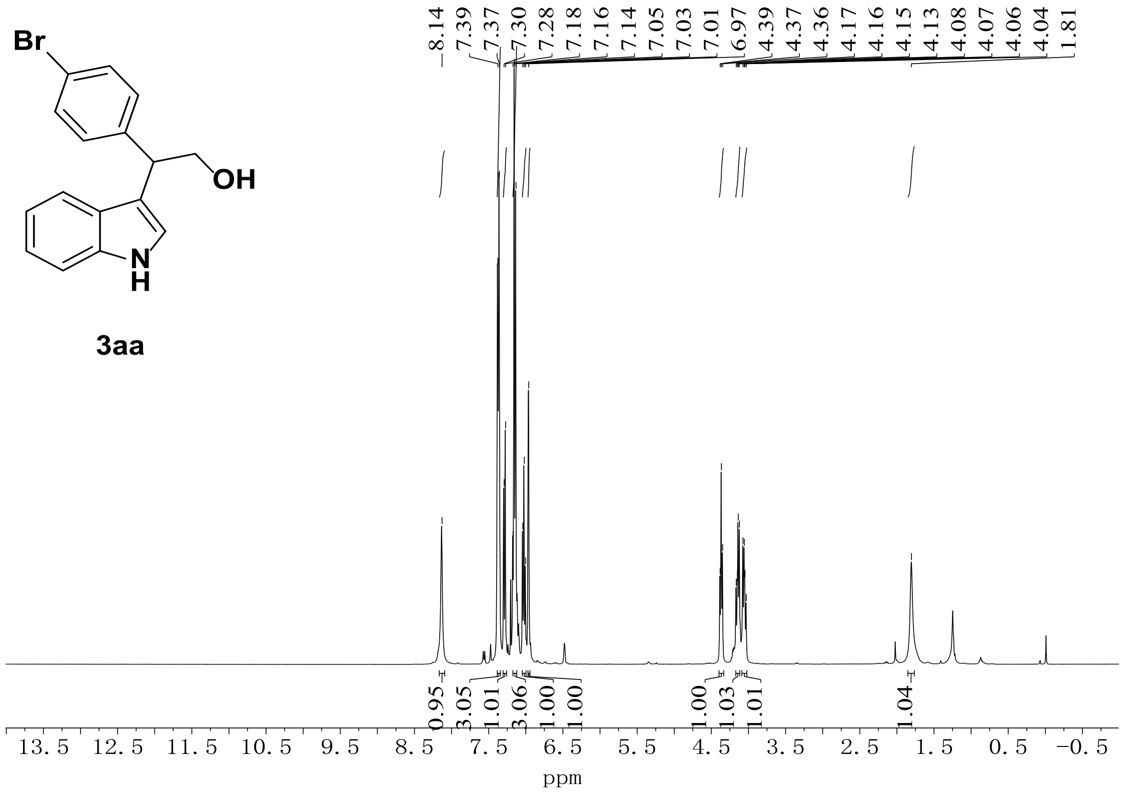

^{1}H NMR spectrum of compound **3aa** (400 MHz, $CDCl_3$, 298 K)

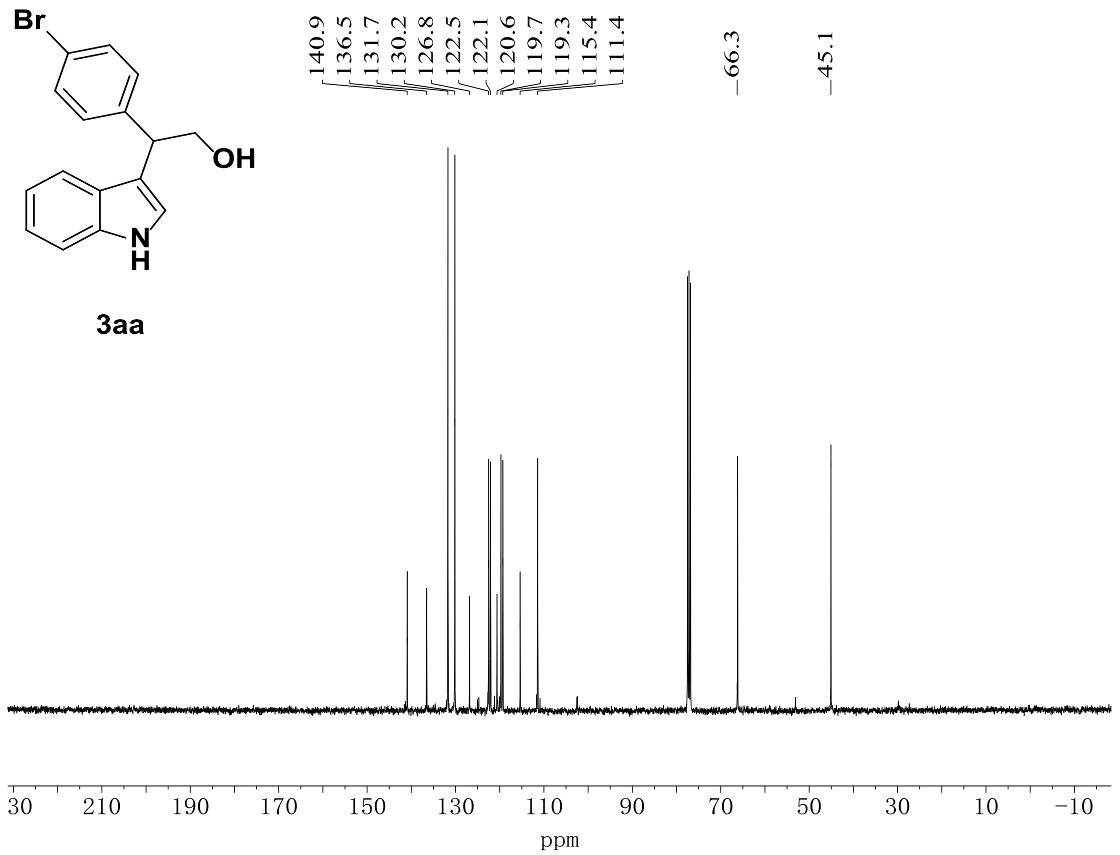

^{13}C NMR spectrum of compound **3aa** (100 MHz, $CDCl_3$, 298 K)

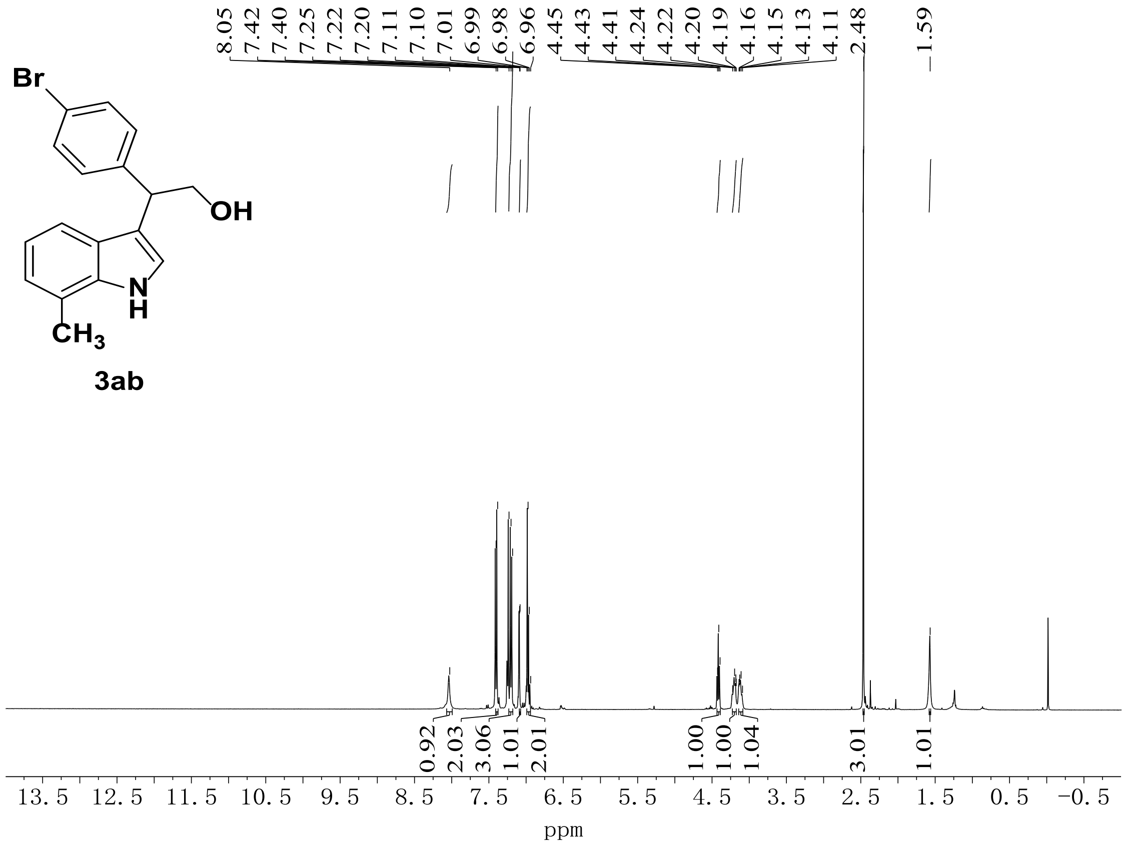

^{1}H NMR spectrum of compound **3ab** (400 MHz, $CDCl_3$, 298 K)

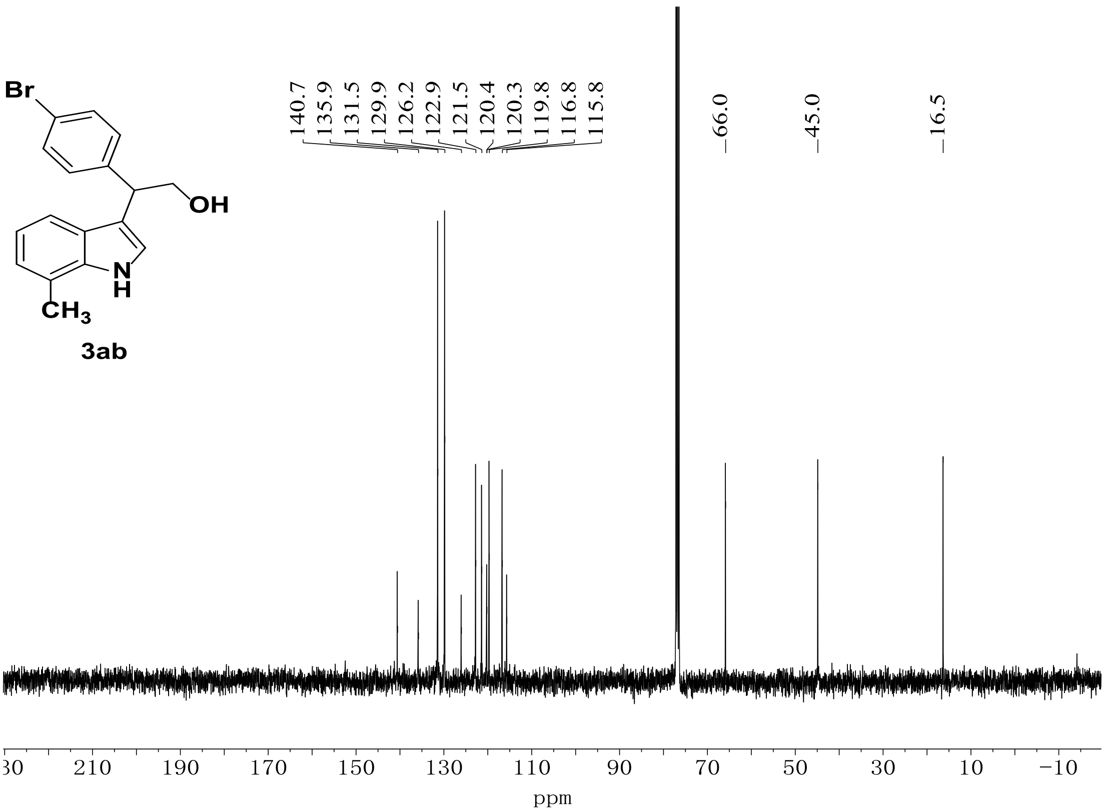

^{13}C NMR spectrum of compound **3ab** (100 MHz, $CDCl_3$, 298 K)

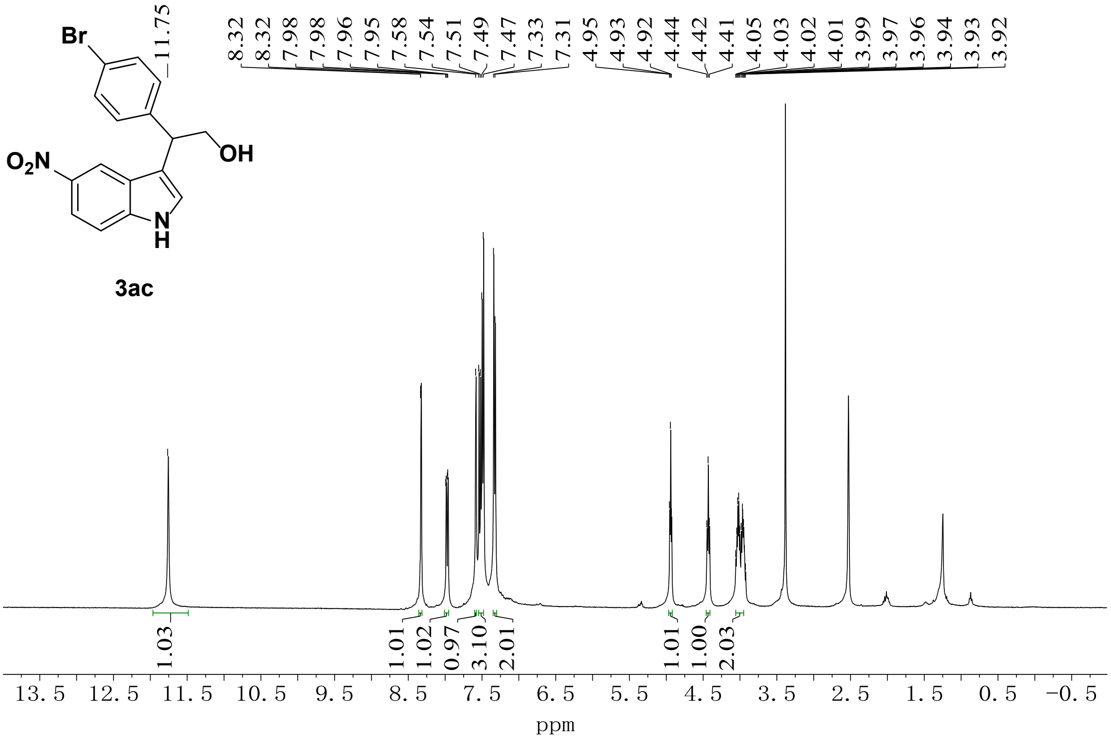

^{1}H NMR spectrum of compound **3ac** (400 MHz, DMSO-*d6*, 298 K)

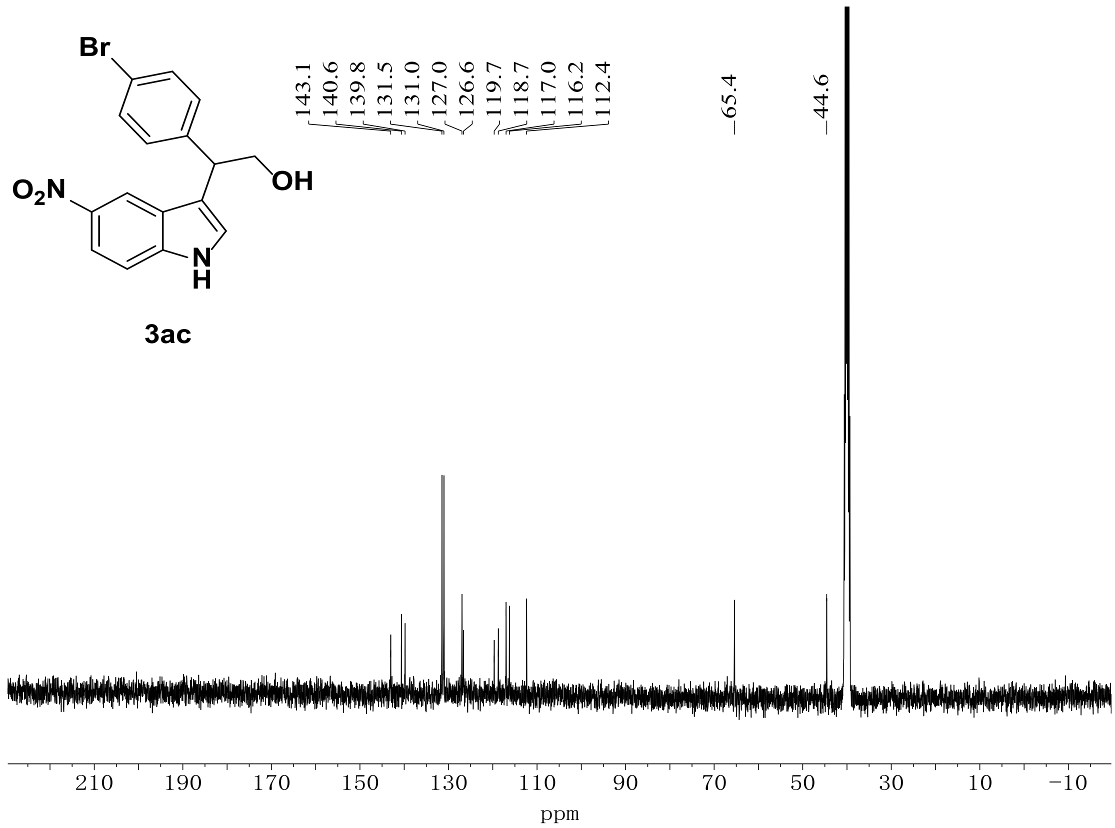

^{13}C NMR spectrum of compound **3ac** (100 MHz, DMSO-*d*6, 298 K)

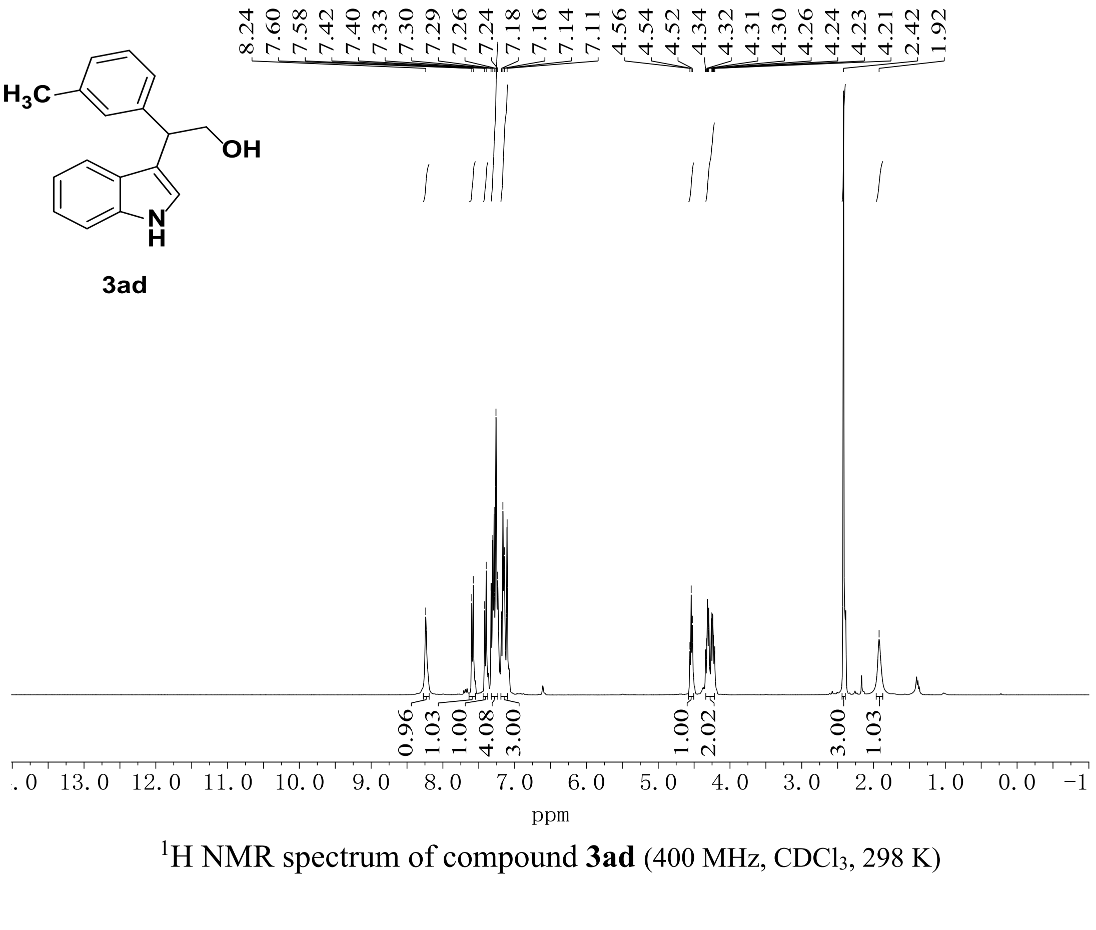

^{1}H NMR spectrum of compound **3ad** (400 MHz, $CDCl_3$, 298 K)

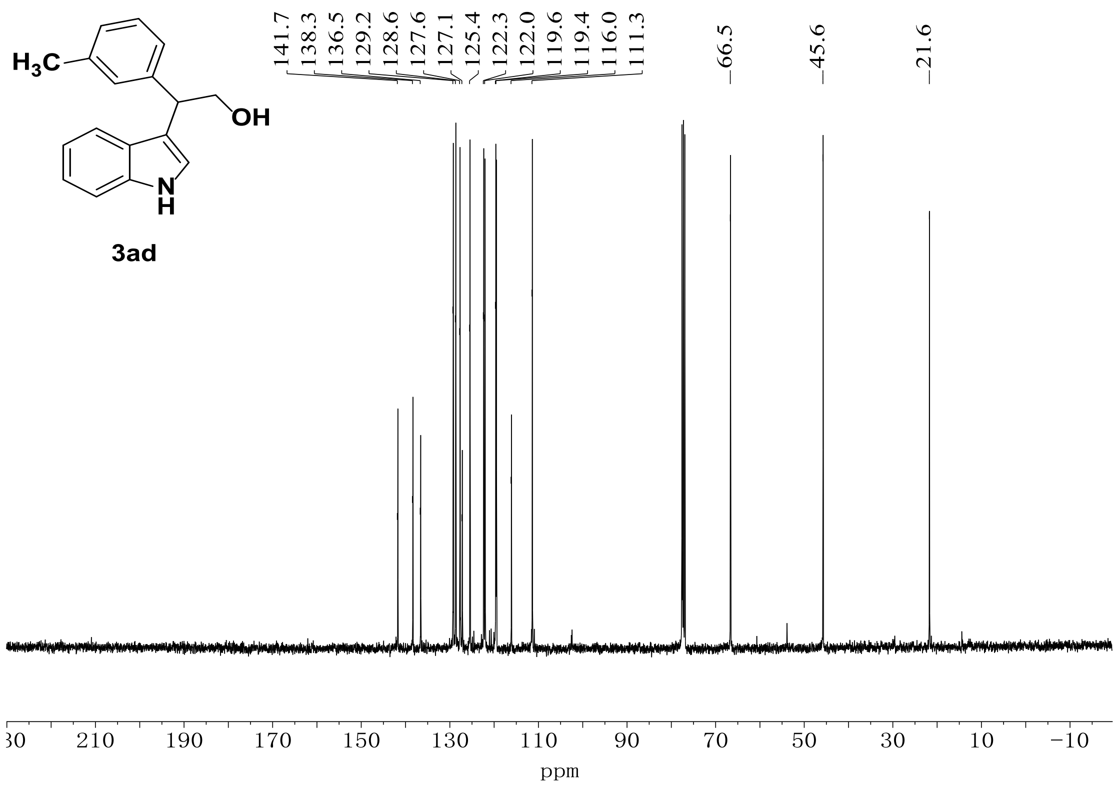

^{13}C NMR spectrum of compound **3ad** (100 MHz, $CDCl_3$, 298 K)

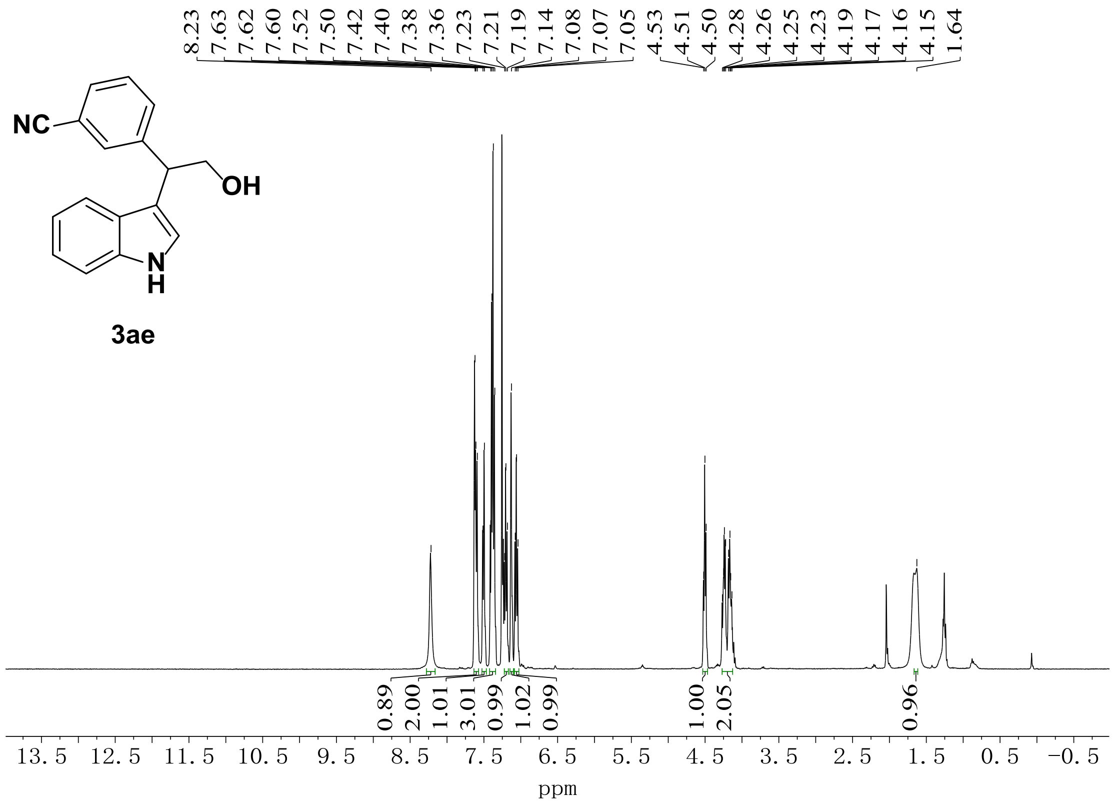

^{1}H NMR spectrum of compound **3ae** (400 MHz, $CDCl_3$, 298 K)

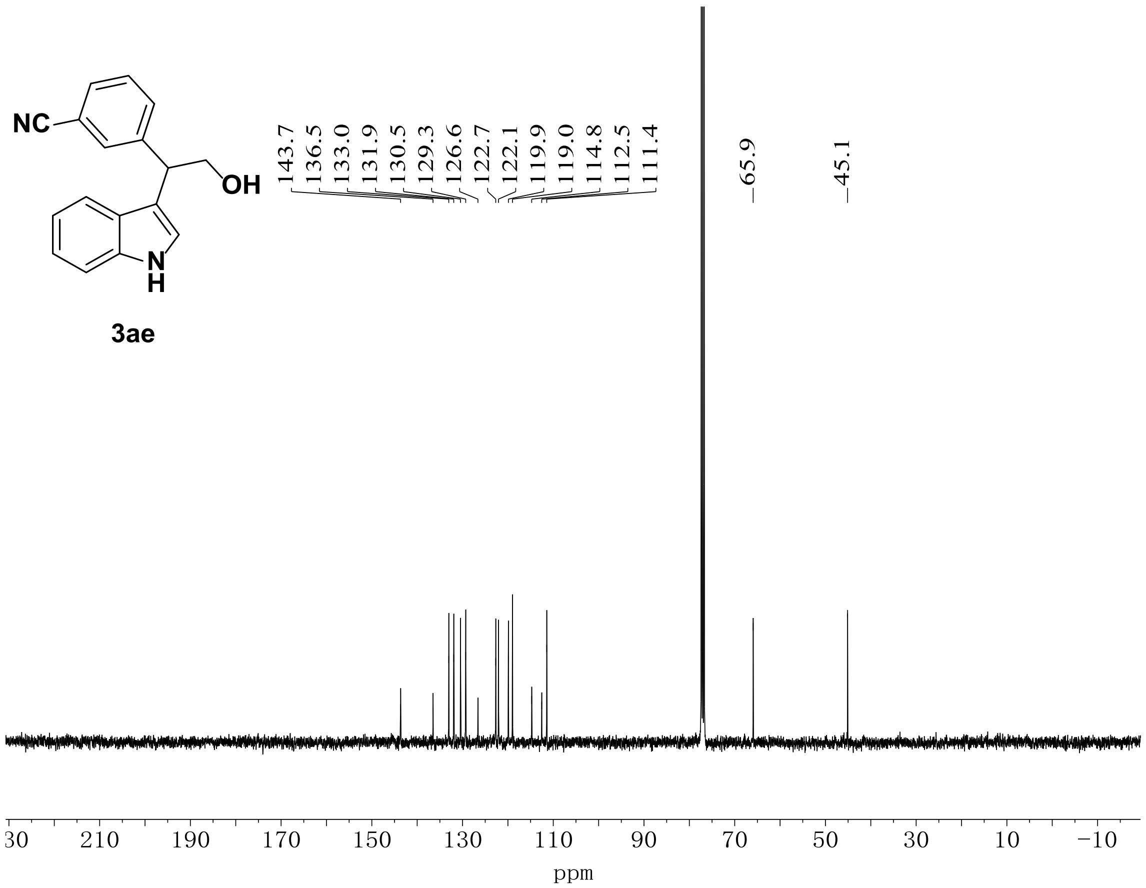

^{13}C NMR spectrum of compound **3ae** (100 MHz, $CDCl_3$, 298 K)

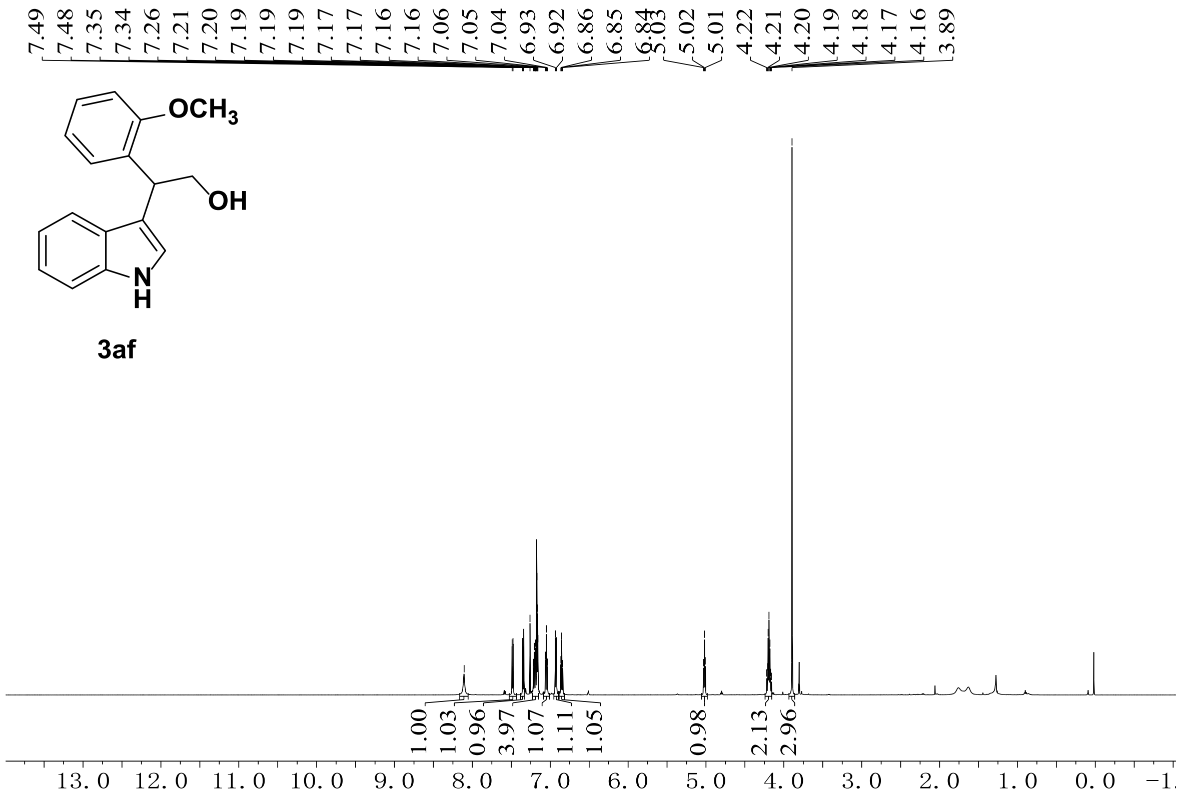

^{1}H NMR spectrum of compound **3af** (600 MHz, $CDCl_3$, 298 K)

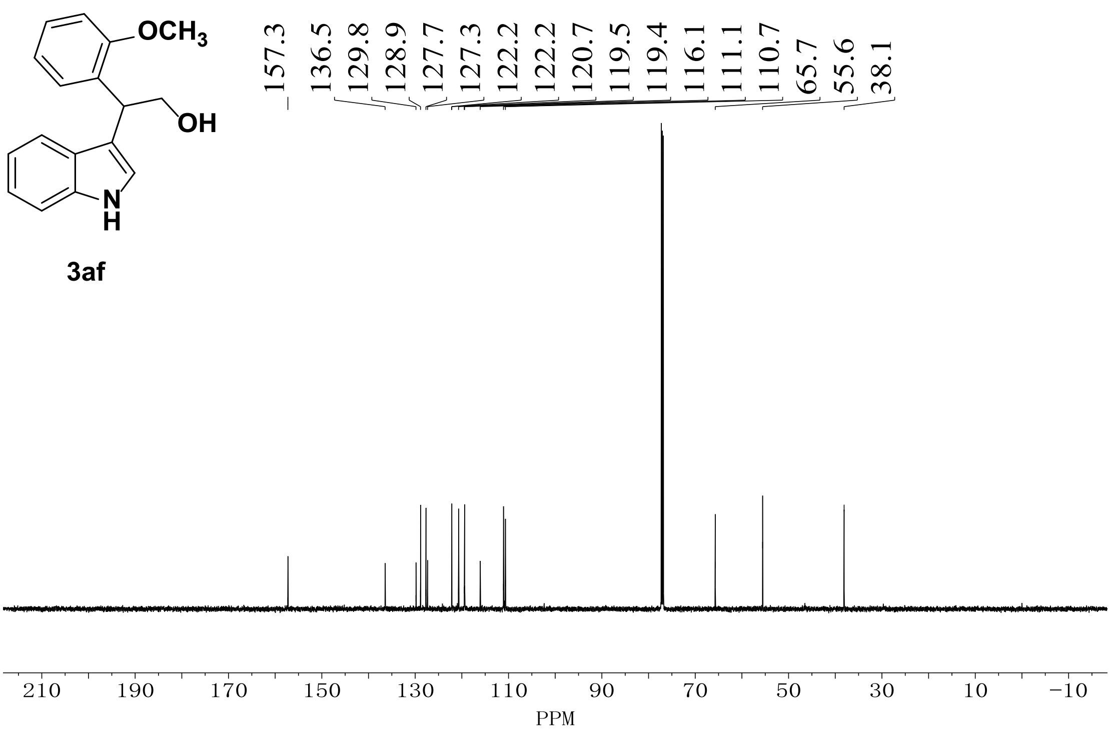

^{13}C NMR spectrum of compound **3af** (150 MHz, $CDCl_3$, 298 K)

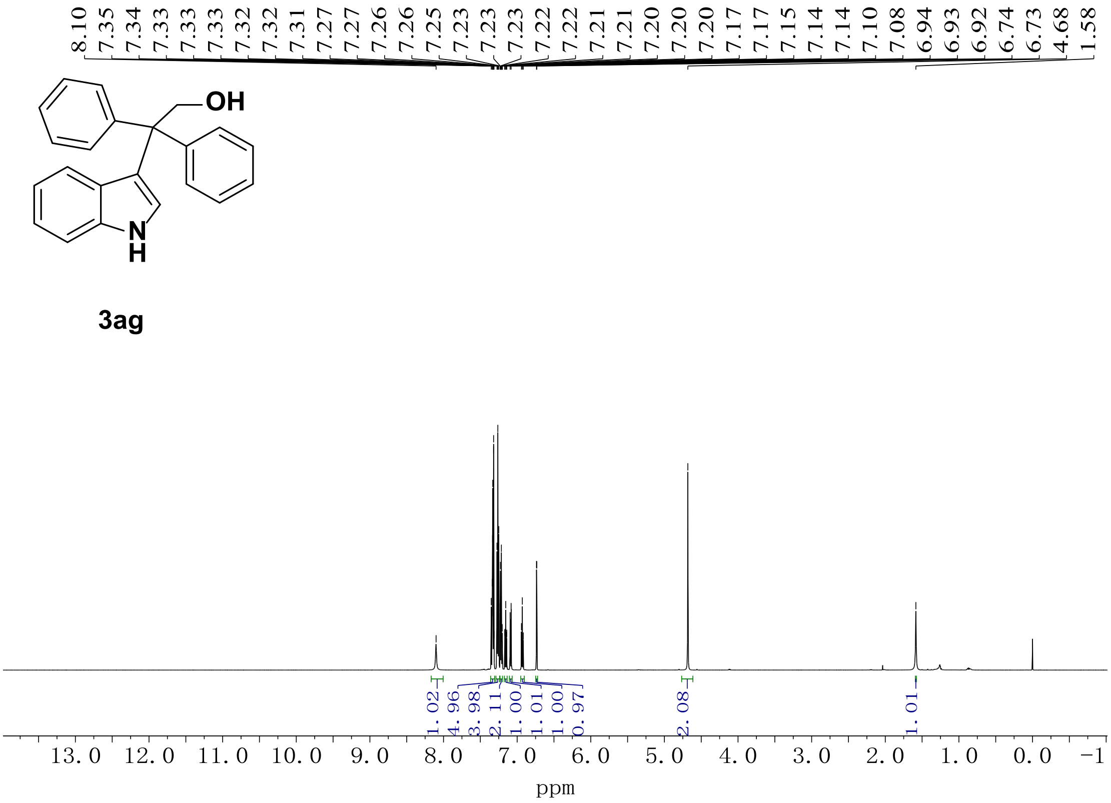

^{1}H NMR spectrum of compound **3ag** (600 MHz, $CDCl_3$, 298 K)

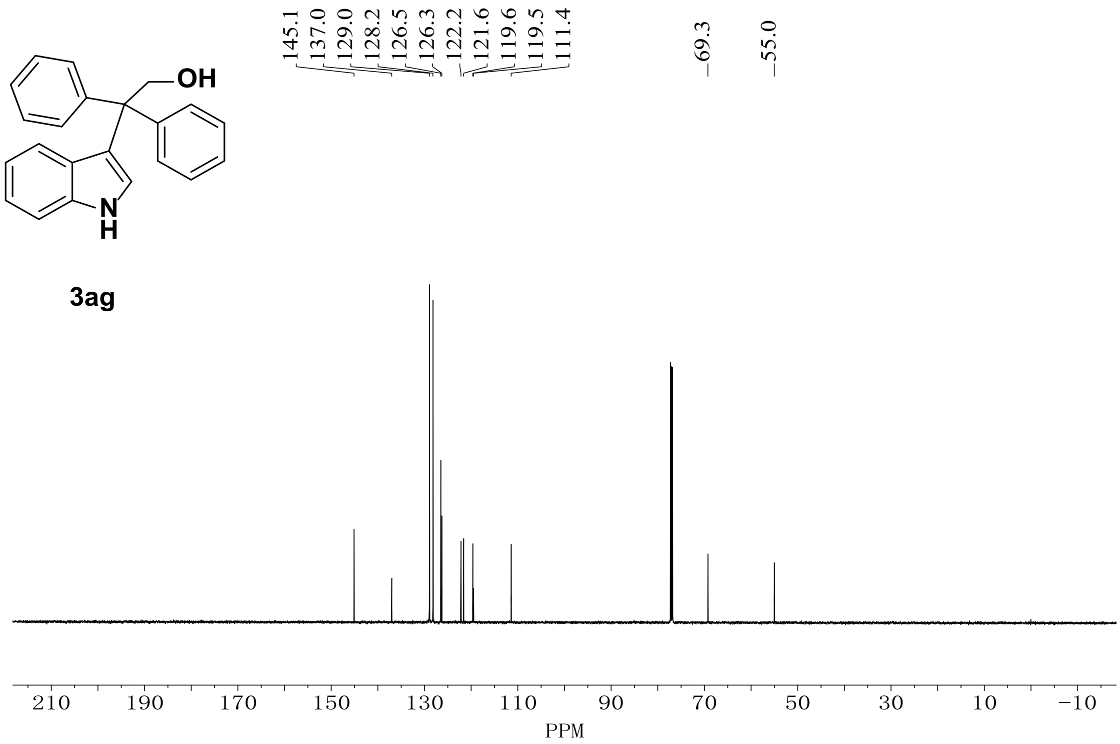

3ag

^{13}C NMR spectrum of compound **3ag** (150 MHz, $CDCl_3$, 298 K)

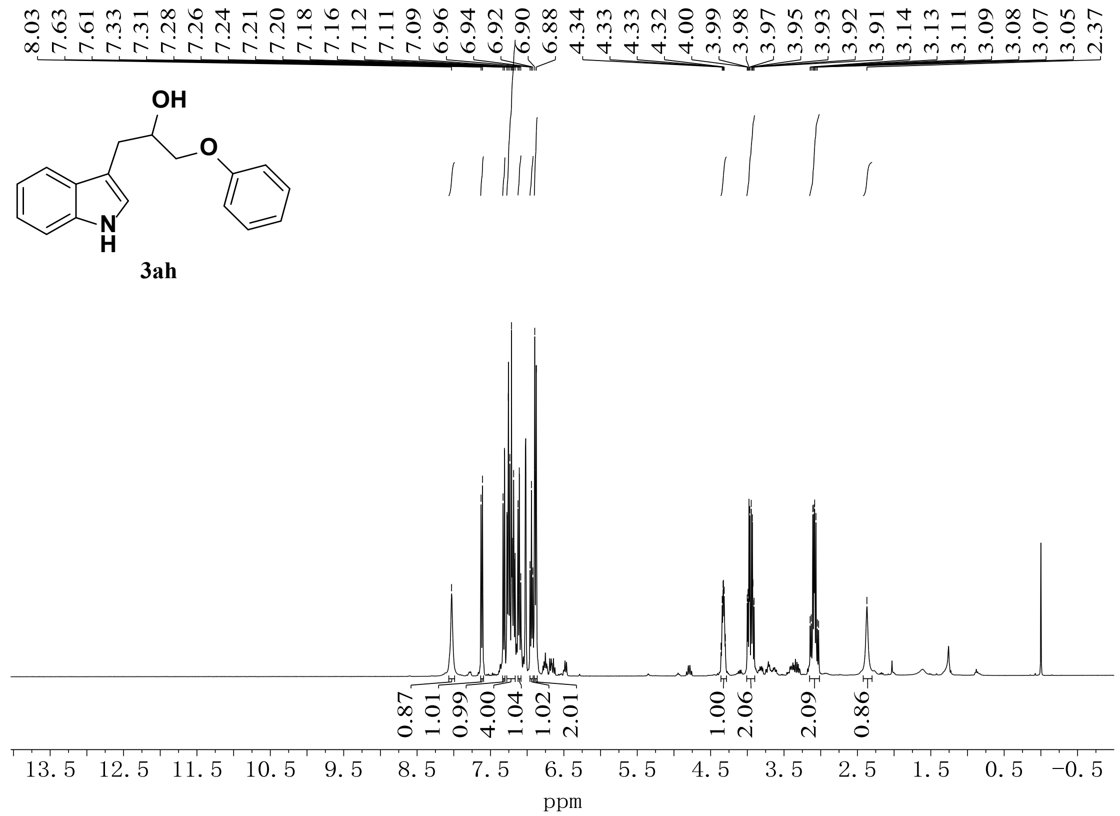

3ah

^{1}H NMR spectrum of compound **3ah** (400 MHz, $CDCl_3$, 298 K)

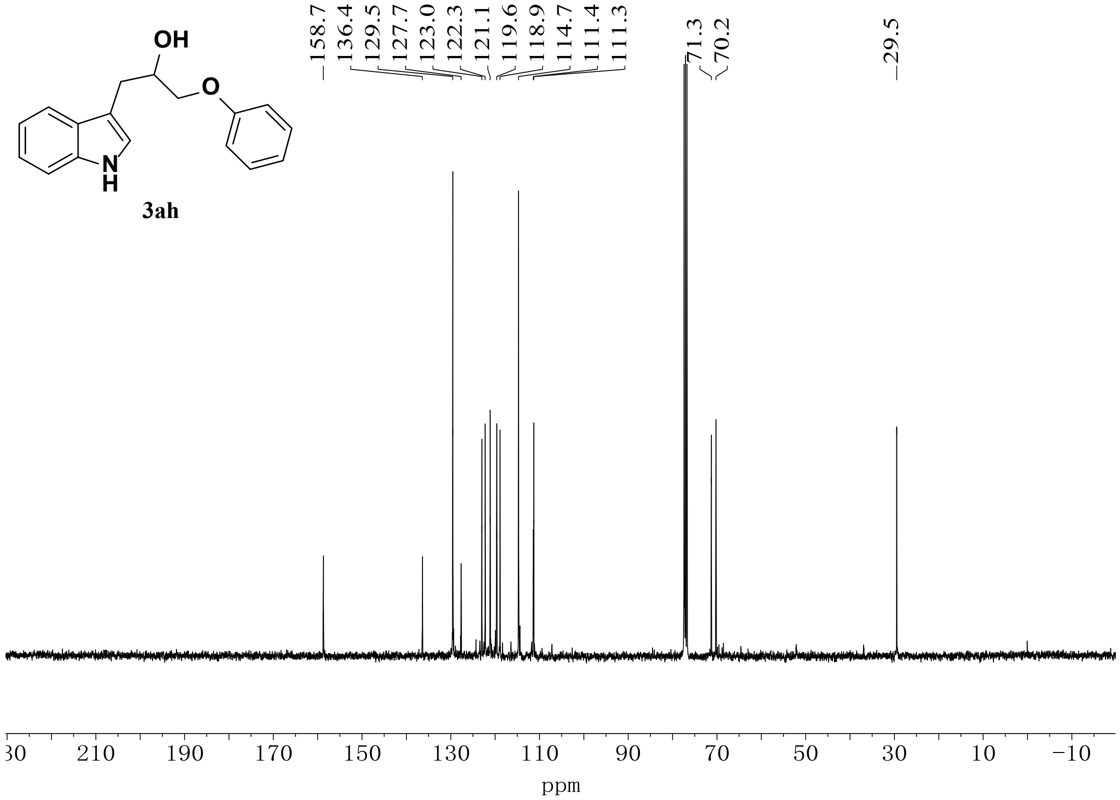

^{13}C NMR spectrum of compound **3ah** (100 MHz, $CDCl_3$, 298 K)

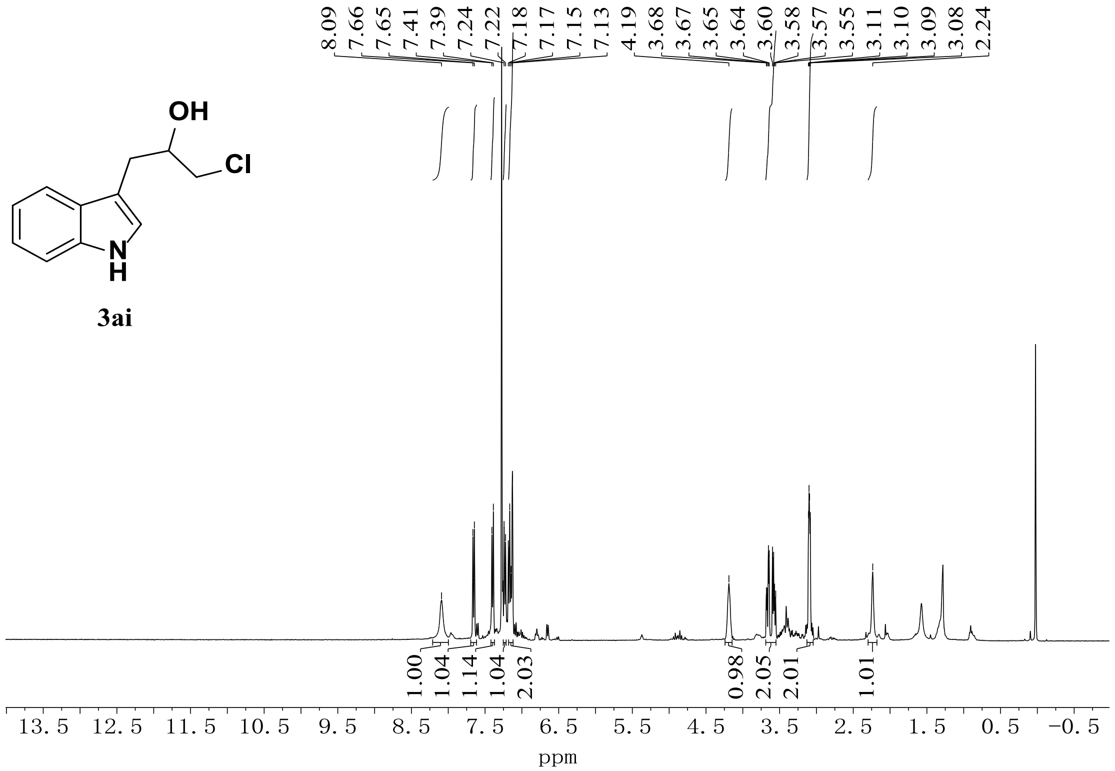

^{1}H NMR spectrum of compound **3ai** (100 MHz, $CDCl_3$, 298 K)

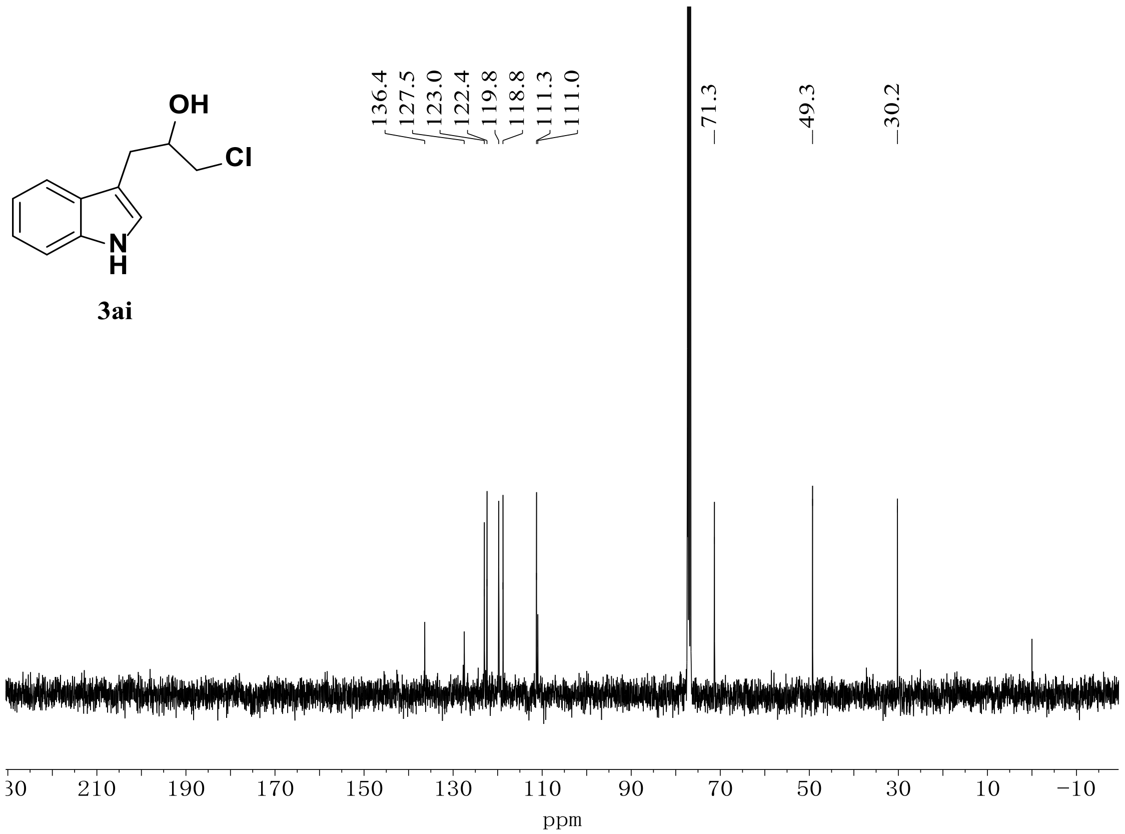

^{13}C NMR spectrum of compound **3ai** (100 MHz, $CDCl_3$, 298 K)

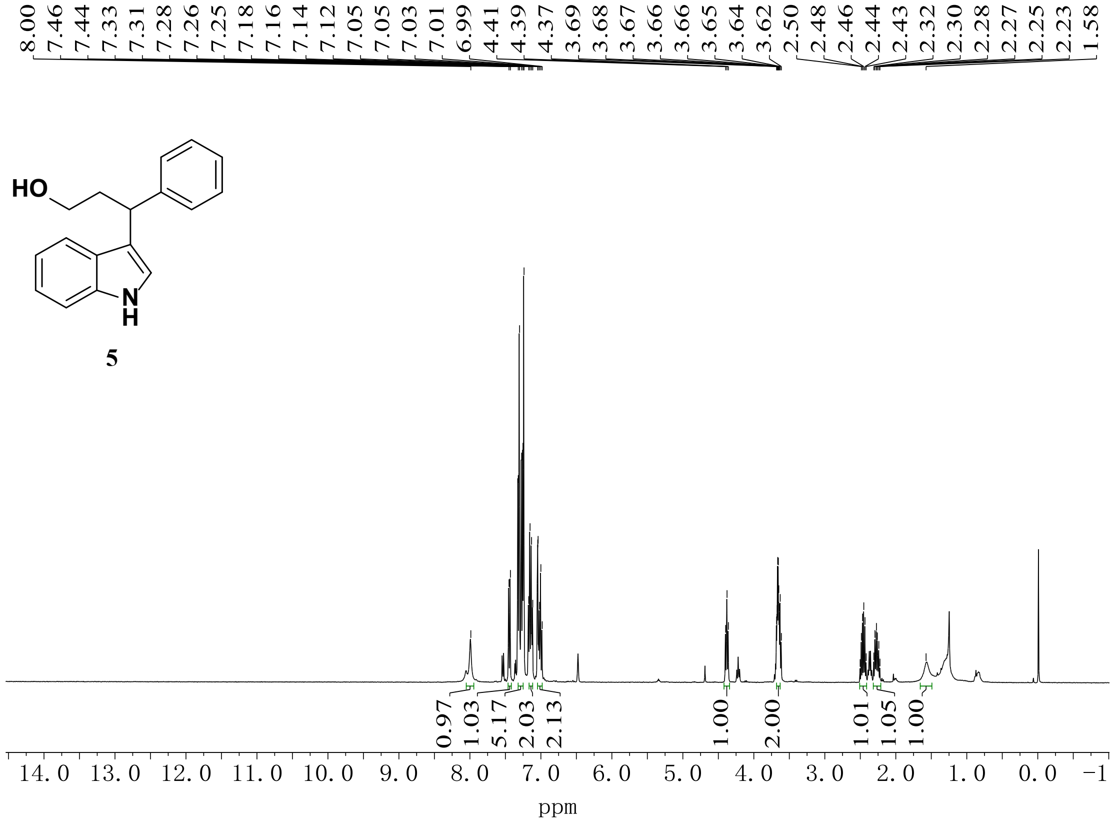

^{1}H NMR spectrum of compound **5** (400 MHz, $CDCl_3$, 298 K)

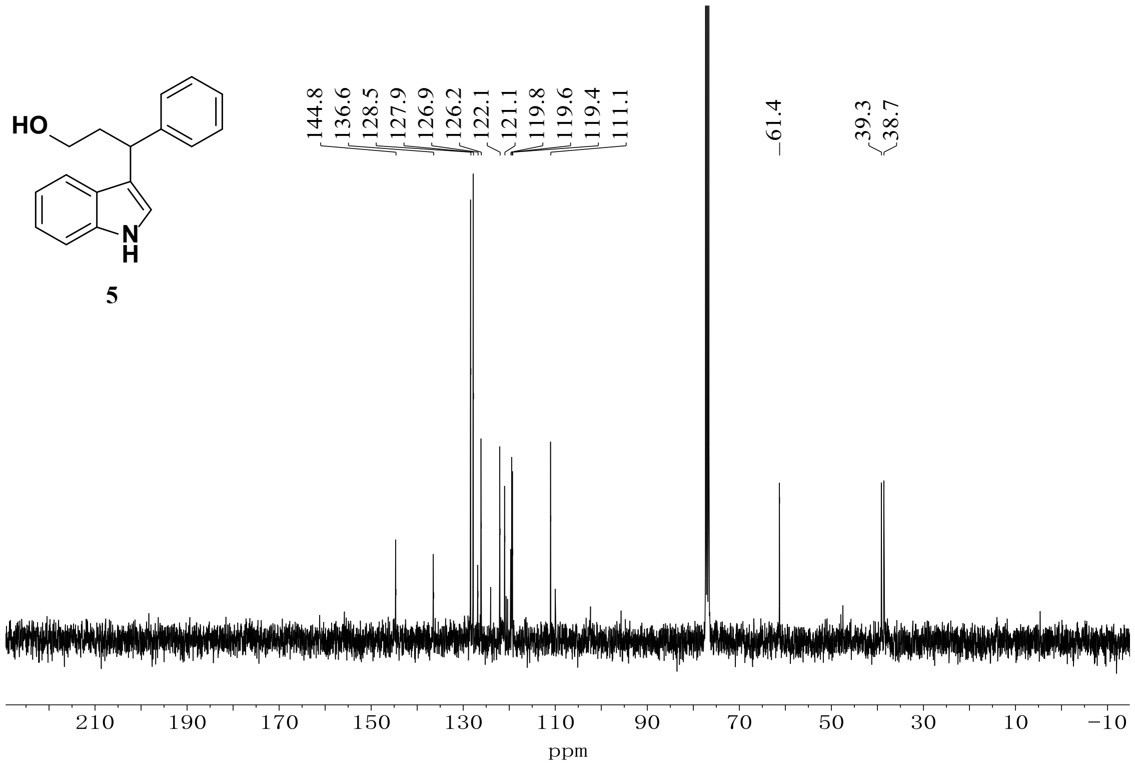

^{13}C NMR spectrum of compound **5** (400 MHz, $CDCl_3$, 298 K)

5. HPLC traces

2-(1H-indol-3-yl)-2-phenylethan-1-ol (3a)

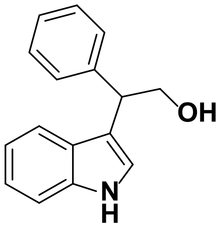

3a

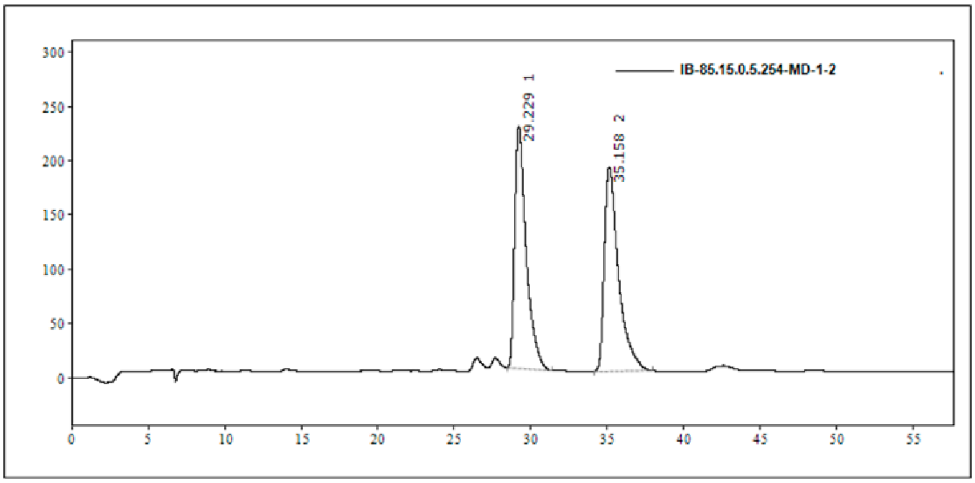

No	Retention Time (min)	Peak Height (mAu)	Peak Height (%)	Peak Area (mAu. s)	Peak Ratio (%)
1	29.229	224.377	54.4	11735.440	49.3
2	35.158	188.315	45.6	12089.146	50.7

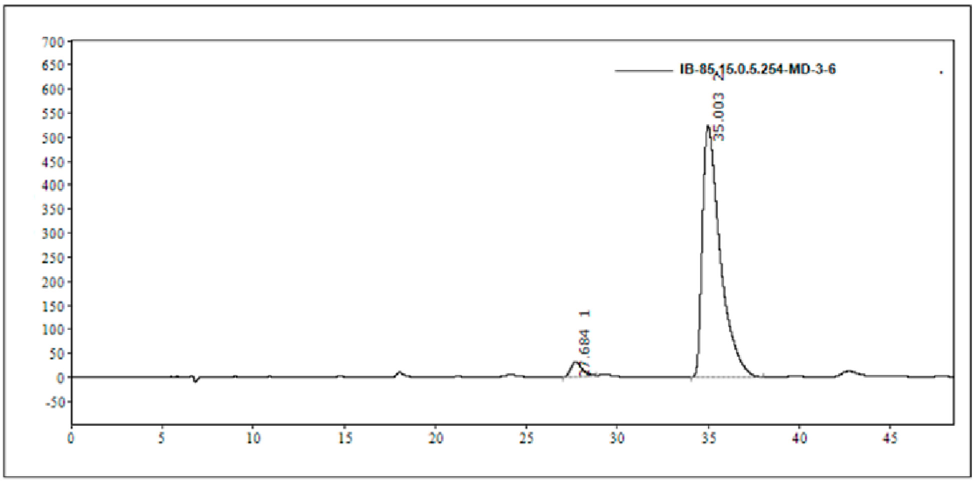

No	Retention Time (min)	Peak Height (mAu)	Peak Height (%)	Peak Area (mAu. s)	Peak Ratio (%)
1	27.684	30.814	5.6	1332.504	3.6
2	35.003	523.475	94.4	35717.840	96.4

2-(6-Methyl-1*H*-indol-3-yl)-2-phenylethan-1-ol (3h)

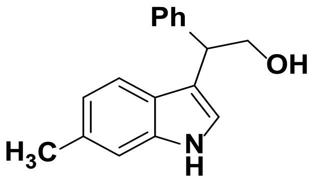

3h

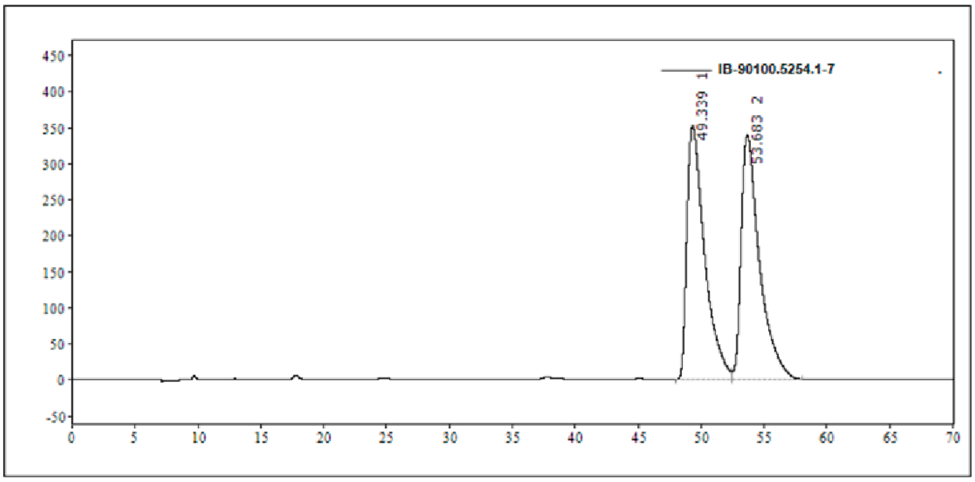

No	Retention Time (min)	Peak Height (mAu)	Peak Height (%)	Peak Area (mAu. s)	Peak Ratio (%)
1	49.339	352.686	51.0	34410.787	49.8
2	53.683	339.173	49.0	34725.146	50.2

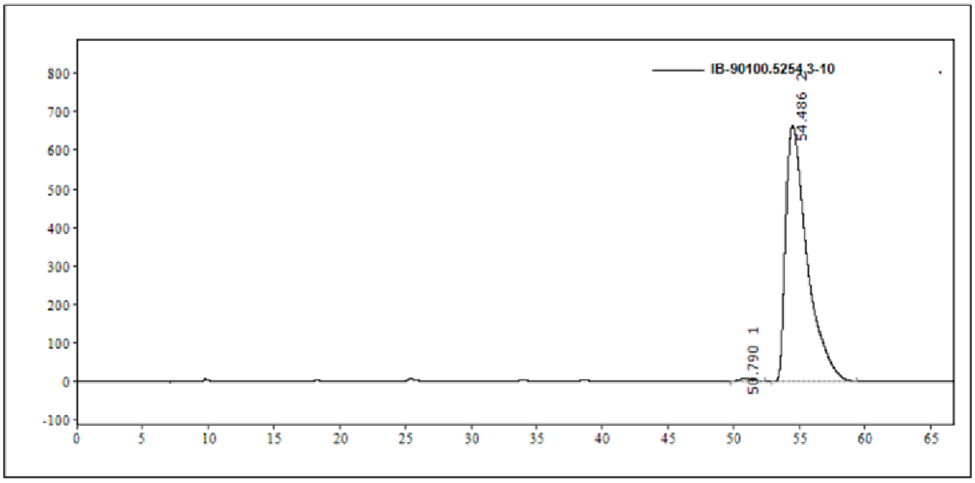

No	Retention Time (min)	Peak Height (mAu)	Peak Height (%)	Peak Area (mAu. s)	Peak Ratio (%)
1	50.790	6.286	0.9	430.728	0.6
2	54.486	664.353	99.1	75044.659	99.4

2-(5-Nitro-1*H*-indol-3-yl)-2-phenylethan-1-ol (3s)

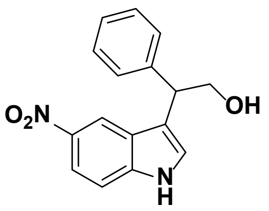

3s

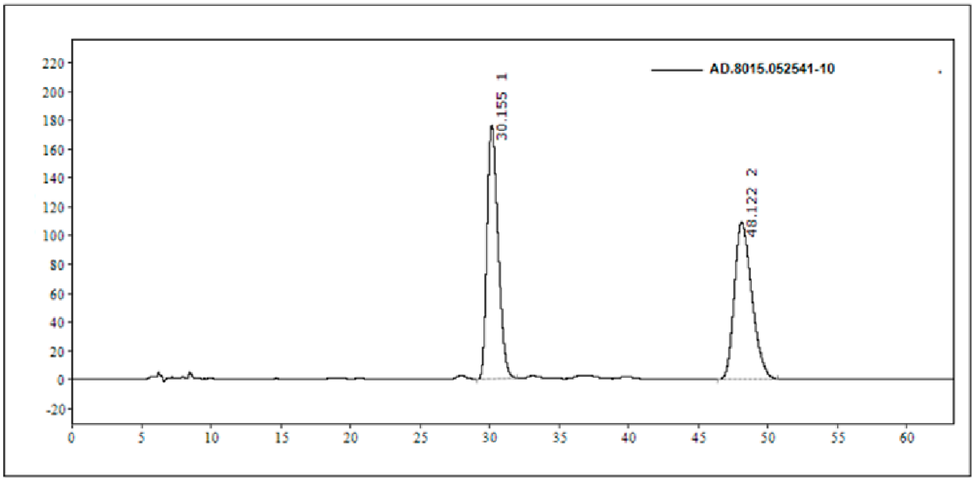

No	Retention Time (min)	Peak Height (mAu)	Peak Height (%)	Peak Area (mAu. s)	Peak Ratio (%)
1	30.155	176.108	61.7	9567.261	49.9
2	48.122	109.246	38.3	9606.871	50.1

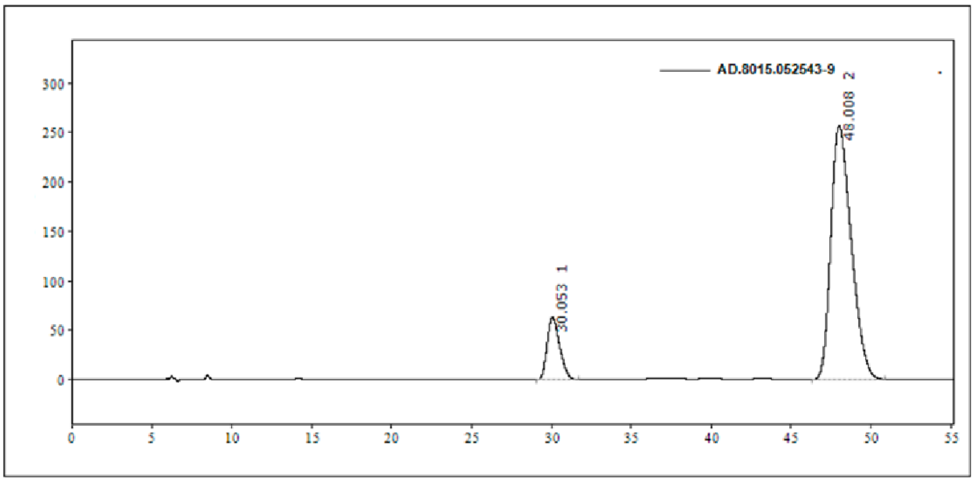

No	Retention Time (min)	Peak Height (mAu)	Peak Height (%)	Peak Area (mAu. s)	Peak Ratio (%)
1	30.053	63.340	19.8	3438.050	13.0
2	48.008	257.003	80.2	22997.380	87.0
